# Lewis base-induced formation of a Ga–Zn cation from a Ga–Zn–Fe–Zn–Ga heteropentanuclear complex

**DOI:** 10.1039/d6sc04662b

**Published:** 2026-07-31

**Authors:** Rajata Kumar Sahoo, Christoph Wölper, Gebhard Haberhauer, Stephan Schulz

**Affiliations:** a Institute of Inorganic Chemistry, University of Duisburg-Essen Universitätsstraße 5-7 45141 Essen Germany stephan.schulz@uni-due.de; b Institute of Organic Chemistry, University of Duisburg-Essen Universitätsstraße 5-7 45141 Essen Germany; c Center for Nanointegration Duisburg Essen (Cenide), University of Duisburg-Esssen Carl-Benz-Straße 199 47057 Duisburg Germany

## Abstract

Heterometallic complexes containing metal–metal bonds provide unique opportunities for exploring unusual bonding and reactivity patterns. Herein we report the synthesis and reactivity of a rare heteropentanuclear complex [L^1^(Et)GaZnFe(CO)_4_ZnGa(Et)L^1^] (2; L^1^ = {HC(C(Me)N(Dipp))_2_}, Dipp = 2,6-^*i*^Pr_2_-C_6_H_3_), containing a Ga–Zn–Fe–Zn–Ga framework, that was synthesized *via* salt metathesis reaction of [L^1^(Et)GaZn(THF)Cl]_2_ (1) with Na_2_Fe(CO)_4_·THF. Reaction of 1 with 2 equivalents of Na_2_Fe(CO)_4_·THF in the presence of 4-*tert*-butylpyridine (4-^*t*^BuPy) afforded the salt [L^1^(Et)GaZnFe(CO)_4_][Na(4-^*t*^BuPy)_3_] (3). The [L^1^(Et)GaZnFe(CO)_4_]^−^ anion was also formed in the reaction of 2 with 4-dimethylaminopyridine (DMAP) together with the [L^1^(Et)GaZn(DMAP)_3_]^+^ cation. Comparable reactions of 2 and 3 with Cp*K yielded L^1^(Et)GaZnCp* (5), which was also obtained from salt elimination reaction of 1 with Cp*K (Cp* = C_5_Me_5_), and the potassium salts [K(THF)_3_][L^1^(Et)GaZnFe(CO)_4_] (6) and [K(DMAP)_3_][L^1^(Et)GaZnFe(CO)_4_] (7), respectively. Combined structural, spectroscopic, and computational studies provide insight into the bonding nature and electronic structure of these heterometallic compounds.

## Abstract 2

A stepwise-synthesized acyclic heteropentanuclear [L^1^(Et)GaZn]_2_Fe(CO)_4_ complex undergoes Lewis base-controlled Zn–Fe bond cleavage to form the [L^1^(Et)GaZnFe(CO)_4_]^−^ anion together with either a cationic Ga–Zn complex or neutral L^1^(Et)GaZnCp*.

## Graphical abstract

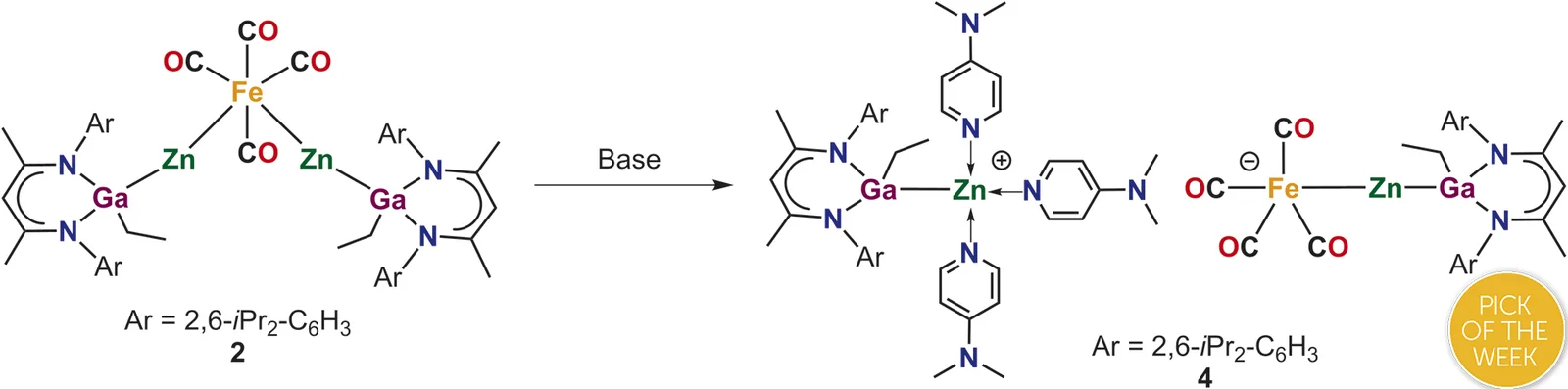

## Introduction

Multimetallic complexes have attracted significant attention due to their unique structures, cooperative reactivity, and potential applications in catalysis, small-molecule activation, materials science,^1^ and the mimicry of enzymatic active sites.^2^ In particular, heteronuclear systems containing different metal centres are of significant interest because the presence of different metals can enable synergistic reactivity that is not accessible in mononuclear or homonuclear complexes.^3^ Mixed-metal complexes incorporating both transition metals and main-group elements are especially attractive, as the combination of different electronic properties can lead to unusual bonding situations and novel reactivity patterns.^4^ For example, recent studies by Hadlington *et al.* demonstrated that a heterotrimetallic [Ga_2_Ni] system promotes alkene hydrogenation through the cooperative involvement of all three metal centres, highlighting the potential of heterometallic assemblies for catalytic transformations (Fig. 1, I).^5^

**Fig. 1:**
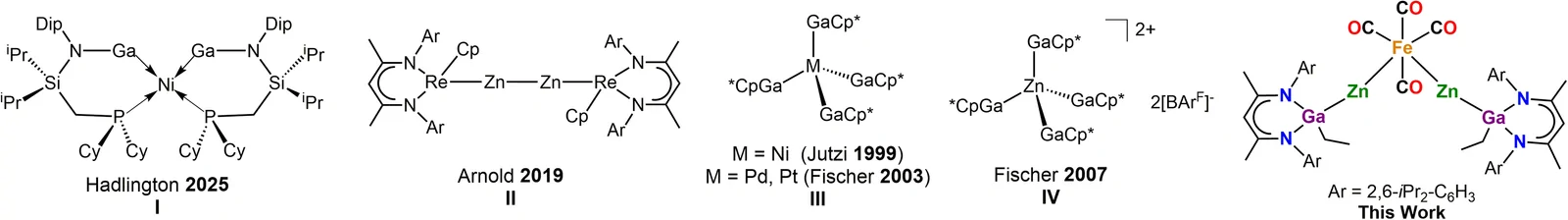
Selected multinuclear mixed transition-metal/main-group metal complexes.

Despite the recent advances made in this field, the controlled synthesis and characterization of heteromultimetallic complexes still remains challenging. While many di- and trinuclear metal complexes have been reported,^6^ acyclic polynuclear complexes containing more than four metal centres are still rare, most likely due to the synthetic challenges associated with controlling the assembly of multiple metal centres in a defined, linear arrangement.^1*f*,4*e*,7^ Representative examples of multinuclear acyclic mixed-metal assemblies include linear tetrametallic Re–Zn–Zn–Re frameworks, which demonstrate the feasibility of constructing extended mixed-metal chains; nevertheless, polynuclear acyclic systems remain rare (Fig. 1, II).^8^ Acyclic frameworks containing both transition and main-group metals are even less explored, despite their potential for cooperative reactivity and interesting structural motifs.^4*e*,*f*^ In this regard, the monovalent gallanediyl GaCp* (Cp* = C_5_Me_5_) was demonstrated to serve as effective metalloligands toward d^10^ metal centres, leading to homoleptic complexes of the type [M(GaCp*)_4_] (M = Ni, Pd, Pt) (Fig. 1, III)^9^ and the zinc analogue [Zn(GaCp*)_4_]^2+^ (Fig. 1, IV).^10^ These compounds prove the strong electron donor properties of GaCp* and its utility in the construction of unusual heterometallic architectures and multinuclear complexes.^9^

Beyond cyclopentadienyl-supported gallium compounds, β-diketiminate ligands have emerged as highly versatile frameworks for stabilizing low-valent main-group and transition-metal species due to their tunable steric and electronic properties.^6*d*^ In particular, ^Dipp^NacNac-stabilized monovalent gallanediyl L^1^Ga (L^1^ = {HC(C(Me)N(Dipp))_2_}, Dipp = 2,6-^*i*^Pr_2_-C_6_H_3_) has attracted considerable interest as reactive low-valent gallium platform, enabling the formation of metal–metal bonds and the synthesis of heterometallic complexes.^6*b*,*c*^ This combination of ligand tunability and the intrinsic reactivity of low-valent gallium centres makes β-diketiminate-stabilized gallium compounds promising candidates for the development of new multimetallic architectures.

The synthetic challenges motivated us to the develop new strategies for the controlled construction of multinuclear heterometallic complexes. Building on our recent studies on the synthesis of acyclic heterotrinuclear complexes, synthesised by reactions of L^1^M (M = Al, Ga) and L^2^Si (L^2^ = {N(Dipp))C(Me)CHC(CH_2_)(N(Dipp)}) with ZnEt_2_,^11^ we aimed to extend this strategy toward multinuclear heterometallic complexes. We here report the synthesis of [L^1^(Et)GaZnFe(CO)_4_ZnGa(Et)L^1^] (2), featuring a five-membered Ga–Zn–Fe–Zn–Ga metal chain. The pentanuclear metal chain in 2 can be selectively cleaved in reactions with Lewis bases, giving access to charge-separated ion pairs containing the [L^1^(Et)ZnFe(CO)_4_]^−^ anion and cations including the [L^1^(Et)GaZn]^+^ cation. Moreover, the synthesis and structural characterization of the neutral mixed-metal heterobinuclear complex [L^1^(Et)GaZnCp*] is reported.

## Results and discussion

Reaction of L^1^Ga with an equimolar amount of EtZnCl in THF solution rapidly gave the heterobimetallic complex [L^1^(Et)GaZn(THF)Cl]_2_ (1) in high yield (92%, Scheme 1). ^1^H and ^13^C{^1^H} NMR spectra confirmed the formation of 1 and show the expected resonances for the Et and L^1^ substituents as well as the coordinating THF molecule. The γ-CH proton in 1 (4.87 ppm) shows an upfield shift compared to L^1^Ga (5.19 ppm) as was previously observed in comparable compounds.^11^

**Scheme 1:**
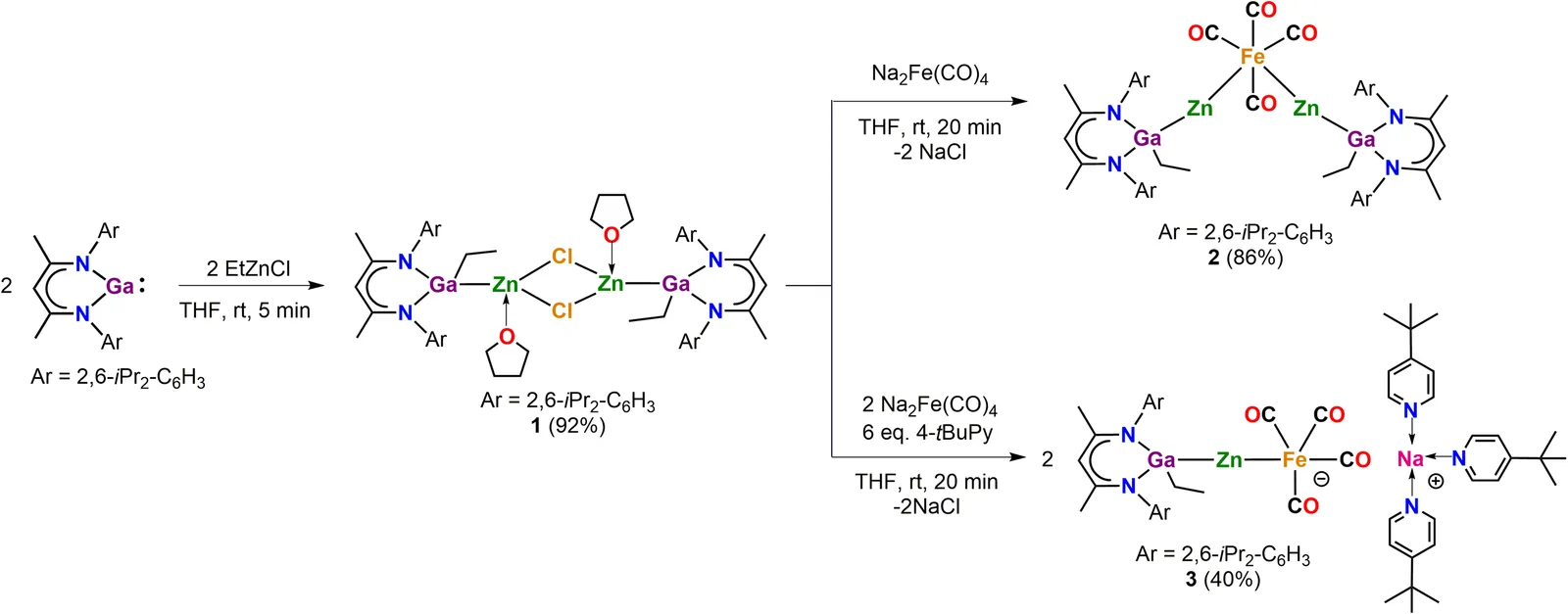
Synthesis of complexes 1–3.

Single crystals of compound 1 were obtained from THF solution upon storage at −3 °C for 24 h. 1 crystallizes in the triclinic space group *P*1̄ with one molecule in the unit cell. Its solid-state structure is depicted in Fig. 2. 1 forms a Cl-bridged dimer in the solid state, and the Ga and Zn atoms each adopt distorted tetrahedral coordination environments. The Ga1–Zn1 bond in 1 (2.4089(3) Å) is in between those reported for [L^1^(Cl)Ga-Zn(Cl)(THF)_2_] (2.3920(6) Å)^10^ and [L^1^(Et)GaZnEt] (2.4265(4) Å),^11^ respectively, whereas the Ga1–C30 bond length in 1 (2.0325(12) Å) is longer than the corresponding Ga–C bond in [L^1^(Et)GaZnEt] (1.986(2) Å).^11^ The Zn1–Cl1 bond in 1 (2.3570(4) Å) is far longer than that in [L^1^(Cl)Ga-Zn(Cl)(THF)_2_] (2.2016(14) Å),^10^ but comparable to that reported for the dimeric µ-Cl-bridged dication [{THF·Ga(L^1^)}Zn(THF)(µ-Cl)]_2_[BAr^F^]_2_ (2.3520(16) Å).^10^

**Fig. 2:**
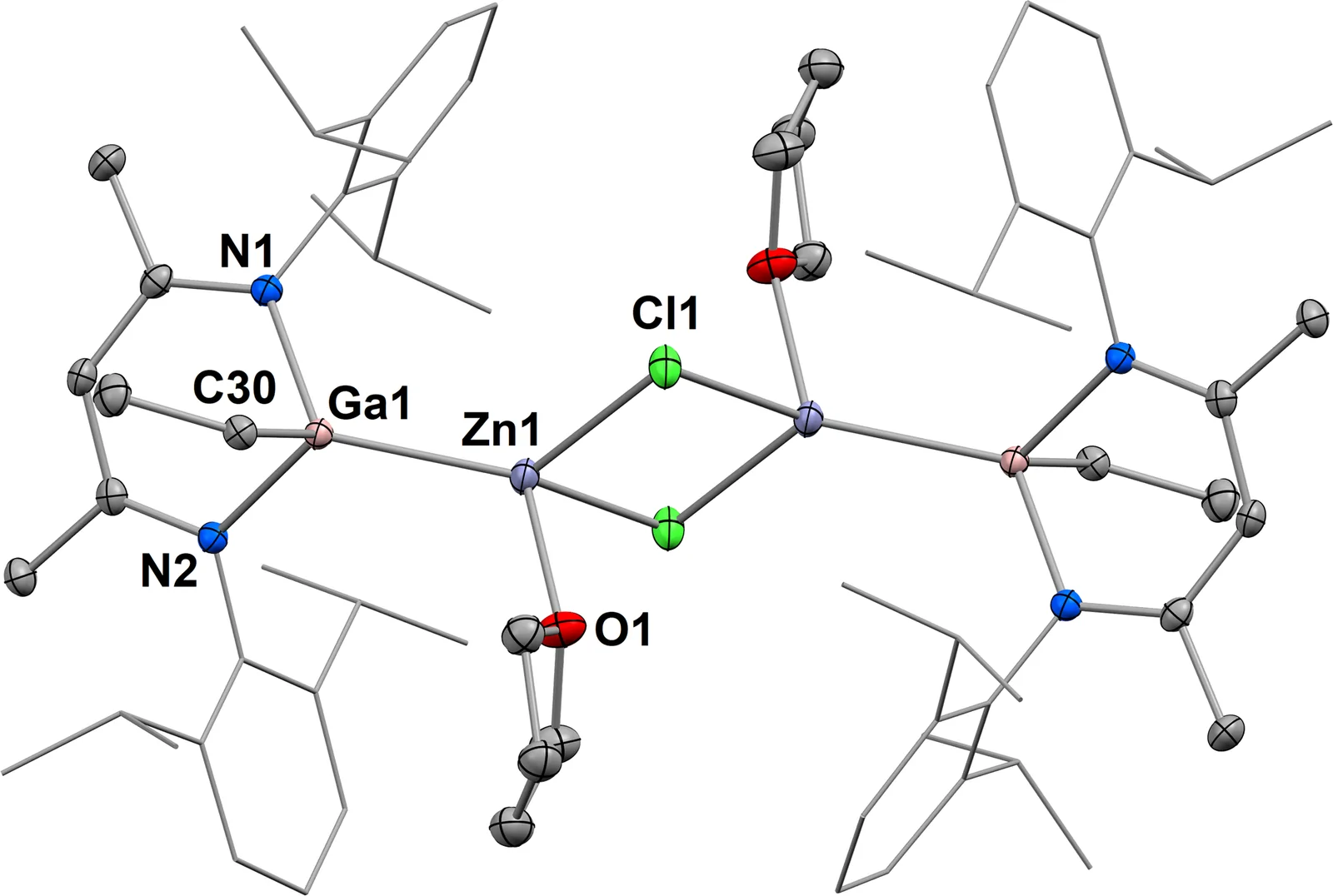
Molecular structure of 1. Displacement ellipsoids drawn at 50% probability level, and hydrogen atoms are omitted for clarity. Labels are omitted on symmetry generated atoms. Selected bond lengths (Å) and angles (°) for 1: Ga1–Zn1 2.4089(3), Ga1–N1 2.0013(10), Ga1–N2 2.0316(10), Zn1–Cl1 2.3570(4), Ga1–C30 2.0325(12), Zn1–O1 2.1571(10), N1–Ga1–Zn1 116.74(3), N2–Ga1–Zn1 117.21(3), N1–Ga1–N2 92.14(4), Cl1–Zn1–Ga1 138.26(2), Cl1–Zn1–O1 91.46(3).

Several attempts to reduce the heterobimetallic complex 1 by use of different reducing agents, including magnesium(i) complexes, Na(naphthalenide), and KC_8_, respectively, only produced complex product mixtures, from which no defined product could be isolated, to date. However, L^1^Ga was formed in all reactions as was proven by *in situ*^1^H NMR spectroscopy, demonstrating that the Ga–Zn bond in 1 is rather unstable under reductive reaction conditions. Since low-valent main-group species can be stabilized in the coordination sphere of transition metal carbonyl fragments, we became interested to react complex 1 with Collman′s reagent, Na_2_Fe(CO)_4_·THF.^12^ The equimolar reaction in THF solution at ambient temperature rapidly gave [L^1^(Et)GaZnFe(CO)_4_ZnGa(Et)L^1^] (2, Scheme 1), while the reaction with two equivalents of Na_2_Fe(CO)_4_·THF in the presence of six equivalents of 4-*tert*-butylpyridine (4-^*t*^BuPy) yielded [L^1^(Et)Ga-Zn-Fe(CO)_4_][Na(4-^*t*^BuPy)_3_] (3) containing the [L^1^(Et)Ga-Zn-Fe(CO)_4_]^−^ monoanion, respectively. ^1^H NMR spectra of 2 and 3 in THF-d_8_ solution display characteristic γ-CH resonances at 5.25 and 5.07 ppm, which are shifted downfield compared to that of compound 1 (4.95 ppm, THF-d_8_), while ^13^C{^1^H} NMR spectra show carbonyl resonances at 212.2 ppm (2) and 224.1 ppm (3), comparable to related complexes such as [NEt_4_][Fe(CO)_4_(AuIPr)] (226.4 ppm).^13^ Furthermore, the infrared (IR) spectra of 2 and 3 show adsorptions due to terminal CO stretching bands at 2004, 1944, and 1878 cm^−1^ (2) as well as 1950 and 1772 cm^−1^ (3), respectively, which are similar to values previously reported for zinc–iron carbonyl complexes.^14^

Single crystals suitable for X-ray diffraction analysis were obtained by slow diffusion of *n*-hexane into a toluene solution of 2, followed by storage at −30 °C for 24 h. Compound 2 crystallizes in the monoclinic space group *C*2/*c*, with the molecule placed on a two-fold rotational axis, and its solid-state structure is shown in Fig. 3. Complex 2 shows a pentanuclear Ga–Zn–Fe–Zn–Ga framework, in contrast to previously reported binuclear L_3_ZnFe(CO)_4_ (L = py, NH_3_) and dimeric tetranuclear [(IPr)ZnFe(CO)_4_]_2_ complexes.^14*b*,15^ The Fe centre adopts an octahedral coordination environment, whereas the Ga atoms are distorted tetrahedrally and the Zn atoms linearly coordinated. The Ga1–Zn1 bond length in 2 (2.4336(3) Å) is elongated compared to those in 1 (2.4089(3) Å) and [L^1^(Cl)GaZn(Cl)(THF)_2_] (2.3920(6) Å), but almost identical to that in [L^1^(Et)GaZn(Et)] (2.4265(4) Å), respectively. The Zn1–Fe1 bond in 2 (2.4386(3) Å) is shorter than those in [(IPr)ZnFe(CO)_4_]_2_ (2.5221(7) and 2.5293(8) Å)^14*b*^ and is comparable to the mean Zn–Fe bond distance reported in the Cambridge Structural Database (CSD, version 5.45; mean = 2.44(13) Å).^16^

**Fig. 3:**
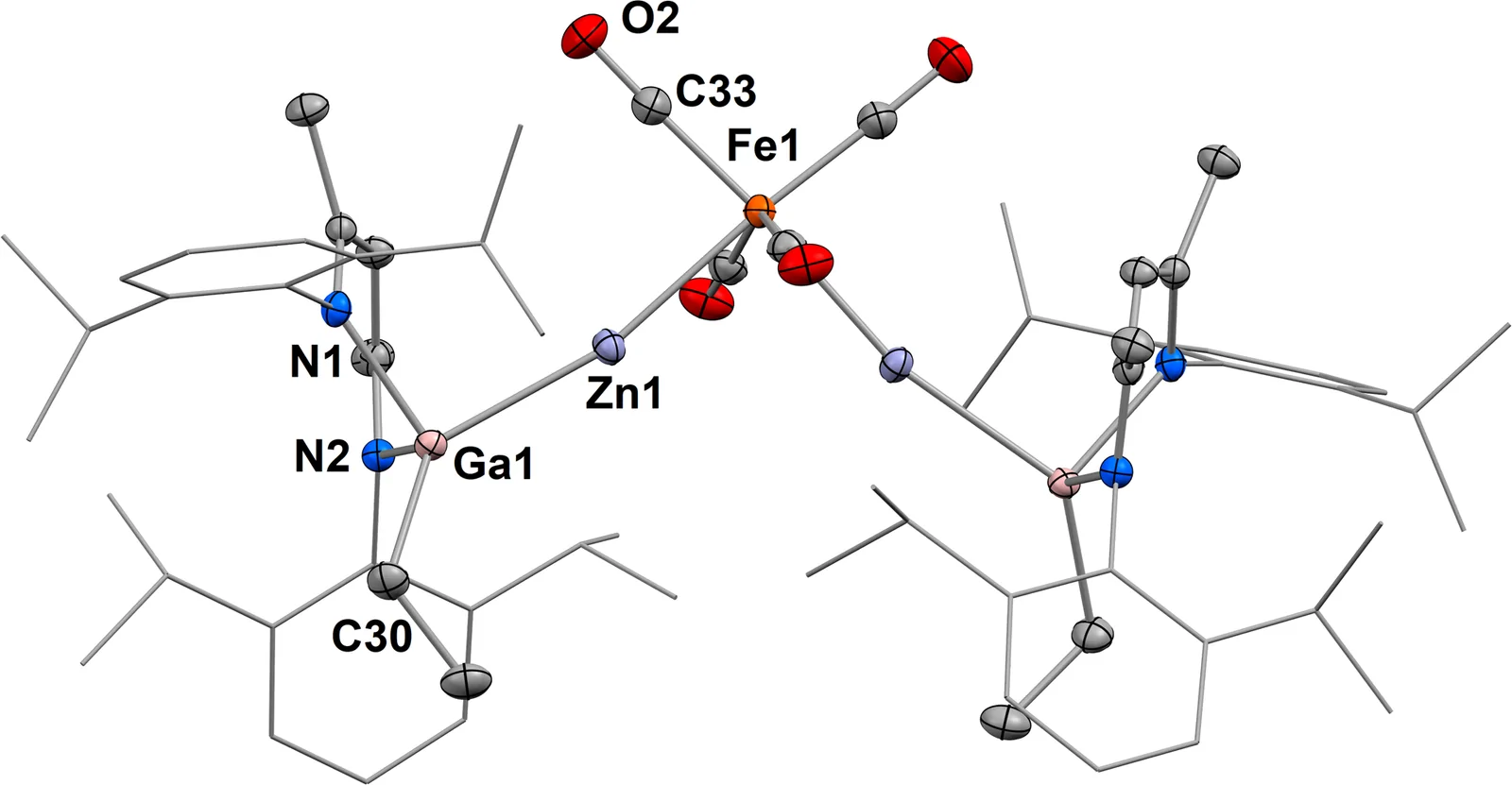
Molecular structure of 2. Displacement ellipsoids drawn at 50% probability level, and hydrogen atoms are omitted for clarity. Selected bond lengths (Å) and angles (°) for 2: Ga1–Zn1 2.4336(3), Zn1–Fe1 2.4386(3), Fe1–C33 1.7760(17), C33–O2 1.147(2), Ga1–N1 1.9944(13), Ga1–N2 1.9905(13), Ga1–C30 1.9768(17), Ga1–Zn1–Fe1 166.904(12), Zn1–Fe1–Zn1’ 90.287(16), N1–Ga1–Zn1 97.25(4), N2–Ga1–Zn1 99.27(4), N1–Ga1–N2 92.82(5).

Single crystals of 3 formed upon storage of a solution of 3 in THF/*n*-hexane mixture at ambient temperature for 5 days. 3 crystallizes in the triclinic space group *P*1̄ (Fig. 4). The Fe centre in the [L^1^(Et)Ga-Zn-Fe(CO)_4_]^−^ anion adopts a trigonal bipyramidal geometry, while the Zn atom is linearly and the Ga atom distorted tetrahedrally coordinated. The Na^+^ cation adopts a distorted trigonal bipyramidal geometry, consistent with a five-coordinate environment defined by three nitrogen donors from 4-*tert*-butylpyridine ligands and two oxygen atoms, one of which arises from a neighbouring unit, resulting in a polymeric structure. The Zn1–Fe1 bond in 3 (2.3723(2) Å) is much shorter than that in 2 (2.4386(3) Å), but far longer than that reported for [Na(THF)_2_]_2_^+^[Zn(Fe(CO)_4_)_2_]^2−^ (2.317(1) Å).^17^ The Ga1–Zn1 bond in 3 (2.4303(2) Å) is comparable to that observed in 2 (2.4336(3) Å). The shortest Na–O bond is observed for the oxygen atom of the CO(2) ligand (Na1–O2 2.3462(10) Å), which is slightly longer than that reported for Na_2_Fe(CO)_4_·1.5C_4_H_8_O_2_ [2.318(5), 2.324(5) Å].^12*b*^

**Fig. 4:**
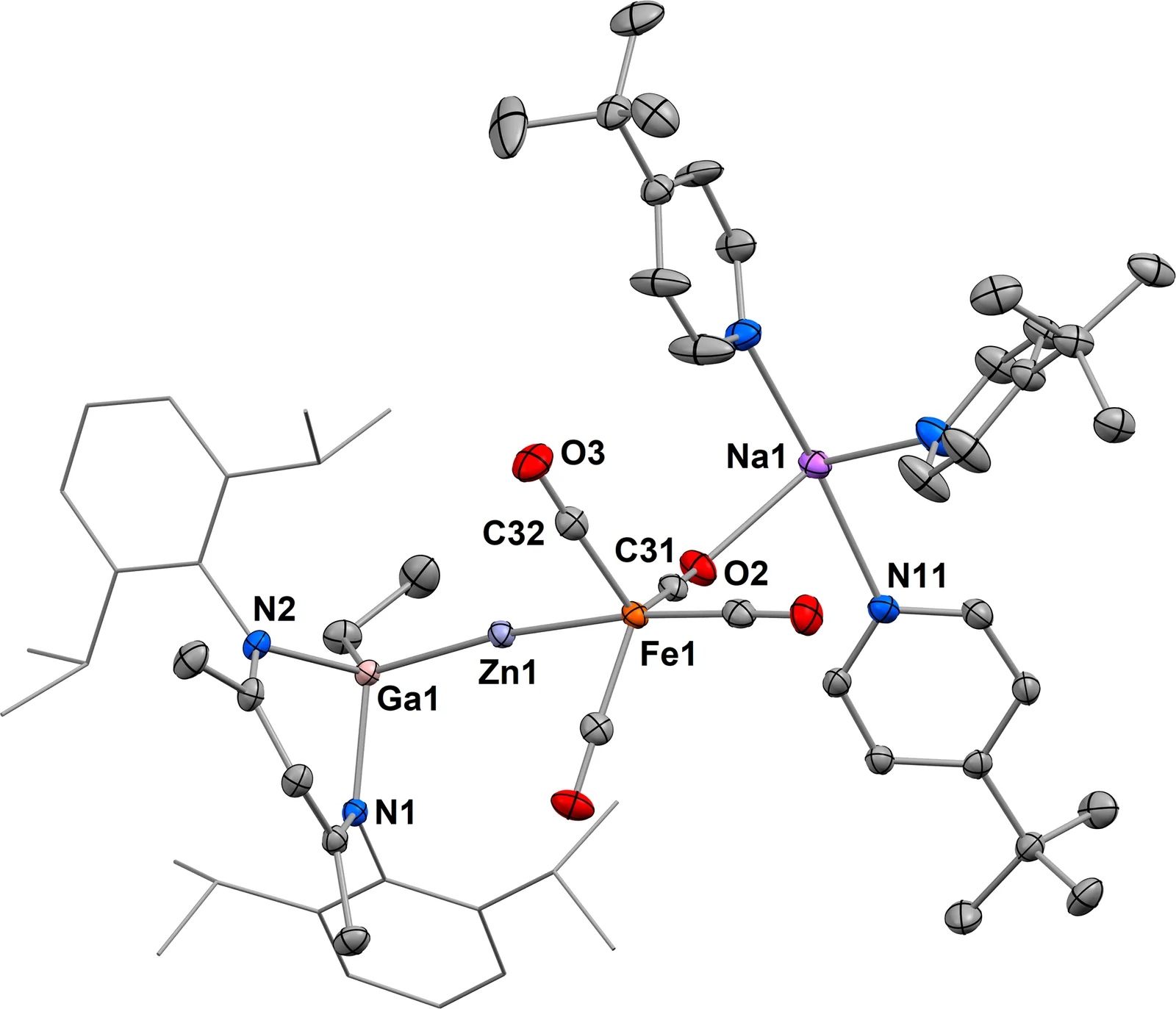
Molecular structure of 3. Displacement ellipsoids drawn at 50% probability level, and hydrogen atoms are omitted for clarity. Selected bond lengths (Å) and angles (°) for 3: Ga1–Zn1 2.4303(2), Zn1–Fe1 2.3723(2), Fe1–C31 1.7553(13), Fe1–C32 1.7729(14), C31–O2 1.1753(16), C32–O3 1.1609(17), Na1–O2 2.3462(10), Na1–N11 2.4265(12), Ga1–C34 1.9892(13), Ga1–N1 2.0074(10), Ga1–N2 2.0125(10), Ga1–Zn1–Fe1 173.535(9), Zn1–Fe1–C30 173.22(4), Zn1–Fe1–C31 74.42(4), Fe1–C31–O2 179.08(13), C31–O2–Na1 130.81(9), N1–Ga1–Zn1 103.85(3), N2–Ga1–Zn1 102.67(3), N1–Ga1–N2 92.77(4), C34–Ga1–Zn1 128.82(4).

The electronic structures of pentanuclear complex 2 and the anion of 3 were investigated by density functional theory (DFT) calculations at the PBE0-D3BJ level of theory (see SI for computational details). A CASSCF(8,8) calculation reveals that the ground state can be described mainly by a single determinant, which justifies the use of DFT methods for this system. While the HOMOs of these systems are mainly localized on the five metal centres in 2 and the three metal centres in the anionic fragment of 3, respectively, the LUMOs are primarily distributed over the L^1^ ligands (Fig. S54 and S55). According to the NBO analysis, the Ga centres show higher natural charges (+0.9*e*) than the Zn atoms (+0.7*e*; Table S3). In contrast, the Fe centres exhibit substantially negative natural charges of −2.8*e* in 2 and −2.6*e* in the anionic fragment of 3. The Mayer bond orders indicate slightly stronger Ga–Zn interactions (0.7–0.8) than Zn–Fe interactions (0.6–0.7) (Table S5).

To gain further insight into the nature of the Ga–Zn and Zn–Fe interactions (dative *versus* electron-sharing), bond energy decomposition analyses^18^ were performed at the B3LYP-D3BJ/TZ2P level using different fragmentation schemes (Tables S6 and S7). In this approach, the fragmentation, which results in an orbital interaction term closer to zero, gives the commonly accepted chemical interpretation (dative or electron-sharing, respectively).^19^ Comparison of the different fragmentation patterns indicates that both the Ga–Zn and Zn–Fe interactions in 2 and in the anionic fragment of 3 are best described as electron-sharing bonds. Nevertheless, for the Zn–Fe interaction in 2, the energy difference between an electron-sharing description and a dative interaction involving donation from a negatively charged iron centre to a zinc cation is negligible (Table S6). In this respect, the Zn–Fe bond in 2 can best be described as a mixture of dative and electron-sharing bonding. Analysis of the first two NOCV deformation densities together with the corresponding orbital interaction energy contributions (Fig. S44–S53) further shows that the Zn–Fe and Zn–Ga interactions are dominated by a single NOCV pair, indicating that the bonding is primarily governed by one principal orbital interaction.

With pentanuclear complex 2 in hand, we became interested in the reactivity of such mixed-metal complexes. Related heterometallic complexes such as [(IPr)ZnFe(CO)_4_]_2_, [(py)_3_ZnFe(CO)_4_], [(NH_3_)_3_ZnFe(CO)_4_], (IPr)(Cl)ZnFeCp(CO)_2_, and [(CO)_4_Fe{µ_2_-Zn(THF)_2_}_2_Fe(CO)_4_] have been previously structurally characterized, however, reactivity studies were not reported to the best of our knowledge.^14,15,20^ In marked contrast, L^1^ZnFeCp(CO)_2_ was used for C–H borylation of benzene (C_6_D_6_) with HBpin,^3*a*^ while L^2^ZnFeR(CO)_2_ (L^2^ = {HC(C(Me)N(Dep))_2_}, Dep = 2,6-Et_2_-C_6_H_3_; R = Cp = C_5_H_5_ or Cp′ = C_5_H_4_Me) showed H_2_ activation under light irradiation.^21^

To investigate the stability of the metal–metal bonds in 2, we initially studied reactions with strong Lewis bases. We initially chose 4-dimethylaminopyridine (DMAP) for this study, since DMAP is not only known to strongly coordinate to Lewis acidic metal centres but also to stabilize low-valent Zn cations. For instance, the reaction of decamethyldizincocene 
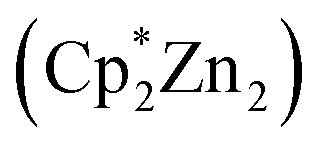
, the first structurally characterized compound containing a central Zn–Zn bond,^22^ with H(OEt)_2_BArF_4_ in the presence of six equivalents of DMAP yielded the first Lewis base-stabilized dicationic dizinc(i) complex, [Zn_2_(DMAP)_6_]^2+^ (Fig. 5, A).^23^2 selectively reacts with three equivalents of DMAP with heterolytic Zn–Fe bond cleavage and subsequent formation of a salt-type compound containing the [L^1^(Et)GaZnFe(CO)_4_]^−^ anion and the [L^1^(Et)GaZn(DMAP)_3_]^+^ cation, respectively (Scheme 2).

**Fig. 5:**
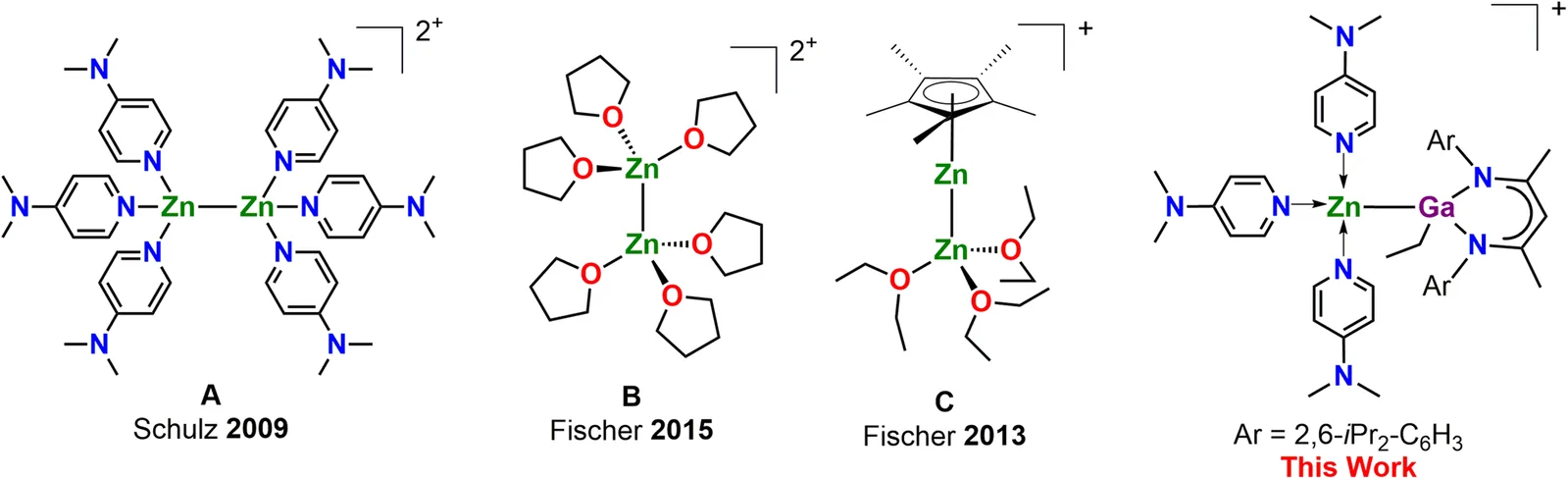
Selected base-stabilized cationic zinc complexes.

**Scheme 2:**
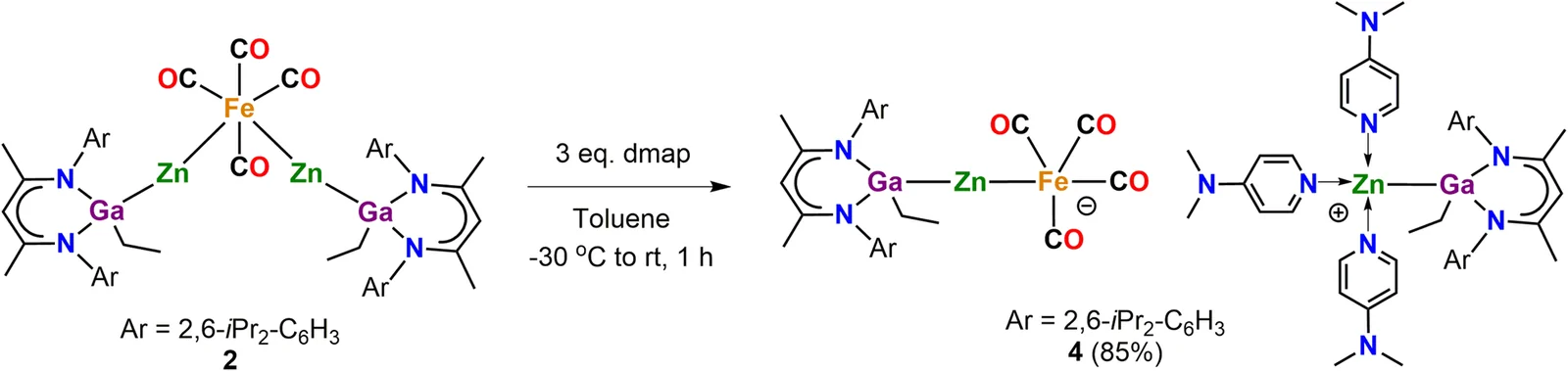
Synthesis of complex 4.

The [L^1^(Et)GaZn(DMAP)_3_]^+^ cation represents a unique structural feature. Although DMAP- and THF-coordinated homoleptic [Zn_2_]^2+^ dications^21–24^ and [Zn_2_]^+^ monocations^25^ have been reported (Fig. 5, B and C),^26^ complex 4 represents to the best of our knowledge the first structurally characterized DMAP-stabilized heteroleptic Ga–Zn^+^ cation.

In contrast to the symmetric complex 2, which shows a single γ-CH resonance (5.07 ppm), 4 exhibits two singlets (4.90, 4.99 ppm) due to magnetically inequivalent L^1^ ligands in the anion and cation. The IR spectrum of 4 shows a pronounced shift of the weakest carbonyl band from 1878 cm^−1^ in 2 to 1811 cm^−1^, consistent with enhanced π-backbonding contribution due to the increased electron density at the Fe centre in the [L^1^(Et)Ga-Zn-Fe(CO)_4_] anion.

Single crystals of 4 suitable for X-ray diffraction analysis were obtained by layering *n*-hexane onto a THF solution and storing the sample at −30 °C for 24 h. Compound 4 crystallizes in the triclinic space group *P*1̄ (Fig. 6).

**Fig. 6:**
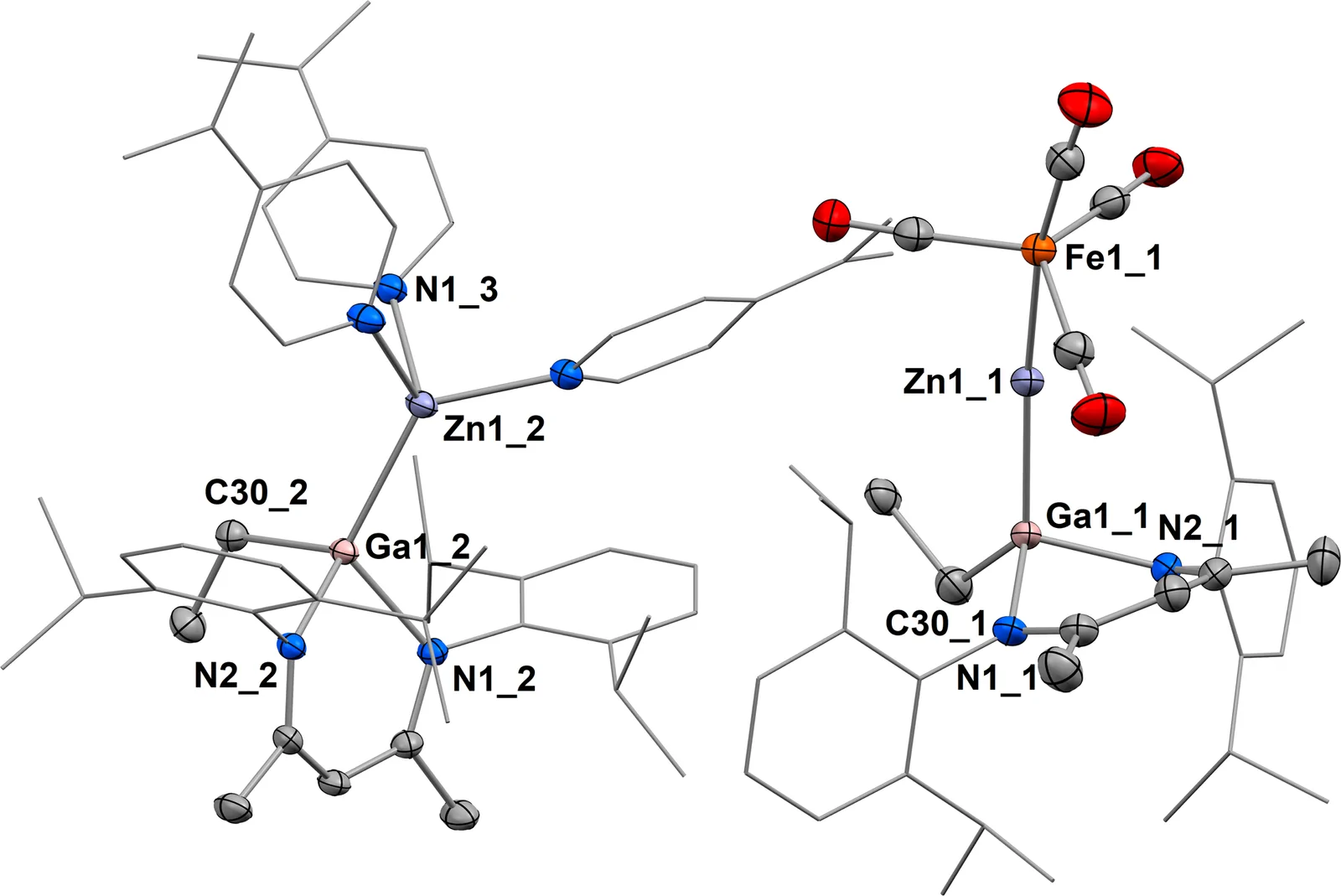
Molecular structure of 4. Displacement ellipsoids drawn at 50% probability level, and hydrogen atoms are omitted for clarity. Selected bond lengths (Å) and angles (°) for 4: Ga1_1–Zn1_1 2.4508(3), Zn1_1–Fe1_1 2.3682(3), Ga1_2–Zn1_2 2.4150(3), Zn1_2–N1_3 2.1646(12), Ga1_1–N1_1 2.0257(13), Ga1_1–N2_1 2.0106(13), Ga1_2–N1_2 2.0266(11), Ga1_2–N2_2 2.0383(12), Ga1_1–C30_1 1.9815(18), Ga1_2–C30_2 2.0259(15), Ga1_1–Zn1_1–Fe1_1 169.657(12), N1_1–Ga1_1–Zn1_1 101.85(4), N2_1–Ga1_1–Zn1_1 103.71(4), N2_1–Ga1_1–N1_1 91.27(5), C30_1–Ga1_1–Zn1_1 134.35(5), C30_1–Ga1_1–Zn1_1 134.35(5), N1_2–Ga1_2–N2_2 92.35(5), N1_2–Ga1_2–Zn1_2 115.31(3), N2_2–Ga1_2–Zn1_2 120.50(3), C30_2–Ga1_2–Zn1_2 115.86(4), N1_3–Zn1_2–Ga1_2 110.33(3).

The [L^1^(Et)Ga-Zn-Fe(CO)_4_] anion shows a coordination environment similar to that observed in compound 3. The Fe1–Zn1 bond length (2.3716(11) Å) in 4 is comparable to that in 3 (2.3723(2) Å), whereas the Ga1–Zn1 bond (2.4621(9) Å) is slightly longer than that in 3 (2.4303(2) Å). In the [L^1^(Et)Ga-Zn(DMAP)_3_] cation, both the Zn and Ga atoms adopt distorted tetrahedral coordination environments, and the Ga1–Zn1 bond length (2.4236(8) Å) is almost identical to that observed in 2 (2.4336(3) Å), but longer than that reported for the dication [{THF·Ga(L^1^)}Zn(THF)(µ-Cl)]_2_[BAr^F^]_2_ (2.3960(11) Å).^10^

We furthermore extended our studies to reactions with Cp*K to prove if the Fe–Zn bond cleavage as observed with the neutral base DMAP also occurs with anionic Cp* groups. Indeed, salt metathesis reactions of complex 2 with one equivalent of Cp*K occurred with selective Fe–Zn bond cleavage and formation of neutral heterobimetallic complex L^1^(Et)GaZnCp* (5) and the corresponding salt [L^1^(Et)GaZnFe(CO)_4_][K(THF)_3_] (6), respectively (Scheme 3).

**Scheme 3:**
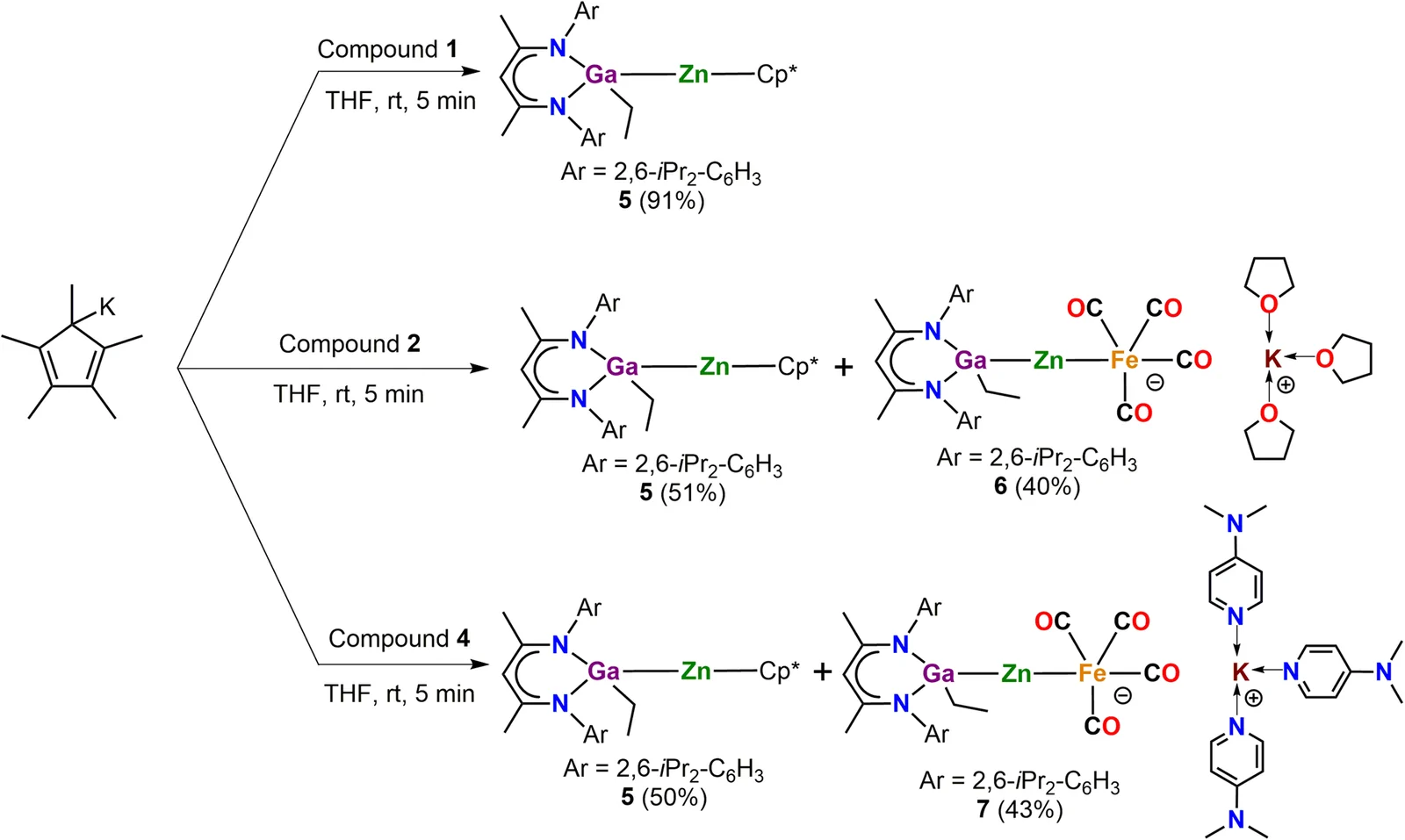
Synthesis of complexes 5–7.

The ^1^H NMR spectrum of complex 5 shows the expected resonances of the substituents, including the γ-CH resonance at 4.88 ppm, which is slightly upfield relative to complex 1 (4.95 ppm), and a singlet at 1.61 ppm due to the Cp* ligand (THF-d_8_). Complex 5 was furthermore independently formed by salt elimination reaction of complex 1 with two equivalents of Cp*K. Moreover, the reaction of complex 4 with one equivalent of Cp*K also gave complex 5 as well as salt [L^1^(Et)GaZnFe(CO)_4_][K(DMAP)_3_] (7), respectively. In this reaction, the anionic Cp* ligand coordinates to the cationic Zn centre, while the K^+^ cation associates with the anionic Fe centre, unambiguously confirming the ion-pair nature of complex 4. Notably, upon treatment of salt 6 with 0.5 equivalents of complex 1 the pentanuclear complex 2 is regenerated, demonstrating the reversible interconversion between complex 2 and salt-type complex 6, indicating that [L^1^(Et)Ga-Zn-Fe(CO)_4_]^−^ might serve as a general precursor for the synthesis of multinuclear heterometallic systems. In compounds 6 and 7, the ^13^C NMR signal for the carbonyl carbon appears at 224.06, and 224.02 ppm, respectively, consistent with related monoanionic complexes 3 and 4, which show resonances at 224.1, and 224.8 ppm, respectively.

Single crystals of complex 5 were obtained from an *n*-hexane solution upon storage at −30 °C for 24 h. 5 crystallizes in the orthorhombic space group *Pna*2_1_ and its solid-state structure is shown in Fig. 7. Superstructure reflections suggest a larger unit cell/modulation however a refinement fails (for details see SI). Consequently, results beyond the connectivity are unreliable but appear realistic.

**Fig. 7:**
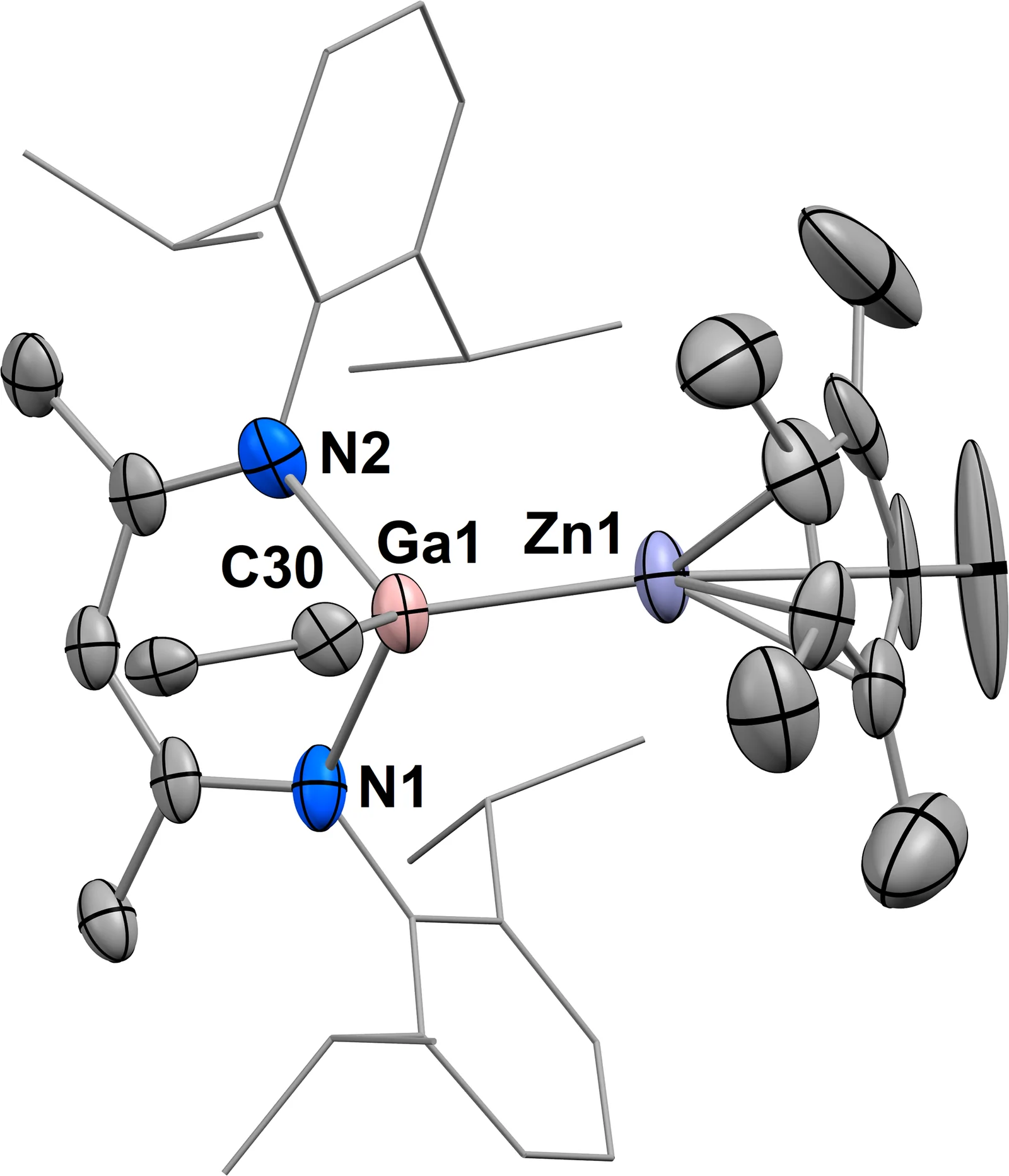
Molecular structure of 5. Displacement ellipsoids drawn at 50% probability level, and hydrogen atoms are omitted for clarity. Selected bond lengths (Å) and angles (°) for 5: Ga1–Zn1 2.3685(14), Ga1–C30 1.968(9), Ga1–N1 2.011(5), Ga1–N2 1.986(5), Zn1–C32 2.305(9), Zn1–C33 2.357(9), Zn1–C34 2.289(8), Zn1–C35 2.265(9), Zn1–C36 2.251(11), N1–Ga1–Zn1 113.79(16), N2–Ga1–Zn1 115.51(16), N1–Ga1–N2 94.1(2), C30–Ga1–Zn1 114.6(3), Cp*(centre)–Zn1–Ga1 171.0(3).

The Ga and Zn centres adopt approximately tetrahedral geometries, and the Cp* ligands are η^5^-bonded. The Ga1–Zn1 bond length (2.3685(14) Å) is slightly shorter than that in 1 (2.4089(3) Å), but comparable to the Ga–Zn distances reported for the L^1^Ga(ZnCp*)_2_ complex (2.3707(6) and 2.4576(6) Å).^27^

## Conclusions

[L^1^(Et)GaZnFe(CO)_4_ZnGa(Et)L^1^] (2) containing a rare heteropentanuclear metal chain was synthesized by salt metathesis reaction of [L^1^(Et)GaZn(THF)Cl]_2_ (1) with Na_2_Fe(CO)_4_·THF, whereas the reaction of 1 with 2 equivalents of Na_2_Fe(CO)_4_·THF in the presence of 4-^*t*^BuPy gave the salt [L^1^(Et)GaZnFe(CO)_4_][Na(4-^*t*^BuPy)_3_] (3) containing a heterotrinuclear metal chain, respectively. Notably, the heterometallic metal chain of 2 underwent selective Lewis base-induced Zn–Fe bond cleavage reactions. While reactions with the neutral Lewis base DMAP yielded salt [L^1^(Et)GaZnFe(CO)_4_][L^1^(Et)GaZn(DMAP)_3_] 4 containing the [L^1^(Et)GaZnFe(CO)_4_]^−^ anion and the [L^1^(Et)GaZn(DMAP)_3_]^+^ cation, the reaction of 2 with Cp*K, in which the Cp* anion serves as Lewis base, gave the neutral complex L^1^(Et)GaZnCp* (5) together with the salt [L^1^(Et)GaZnFe(CO)_4_][K(THF)_3_] (6). The structurally related salt [L^1^(Et)GaZnFe(CO)_4_][K(DMAP)_3_] (7) formed in the reaction of 4 with Cp*K together with complex 5. All new complexes were fully characterized by heteronuclear NMR and IR spectroscopy, single crystal X-ray diffraction (sc-XRD), and computational studies. We believe that this synthetic strategy may become generally applicable for the synthesis of well-defined heteromultimetallic complexes containing both main group and transition metals, and that the monoanionic species [L^1^(Et)Ga-Zn-Fe(CO)_4_]^−^ may serve as a useful precursor for multinuclear heterometallic complexes. Moreover, stoichiometric small molecule activation reactions as well as catalytic reactions of the heteronuclear metal complexes are currently investigated.

## Author contributions

R. K. Sahoo, conceptualization, investigation (synthesis, characterisation), writing – original draft; C. Wölper, investigation (sc-XRD); G. Haberhauer, quantum chemical calculations, supervision; S. Schulz, resources, supervision, writing – review and editing.

## Conflicts of interest

There are no conflicts to declare.

## Supplementary Material

SC-017-D6SC04662B-s001

SC-017-D6SC04662B-s002

## Data Availability

CCDC 2559142 (1), 2559143 (2), 2559144 (3), 2559145 (4) and 2559146 (5) contain the supplementary crystallographic data for this paper.^28*a*–*e*^ Supplementary information (SI): experimental procedures, NMR and IR spectra, crystallographic data and computational details. See DOI: https://doi.org/10.1039/d6sc04662b.
