# Supplementary material for: Lewis base-induced formation of a Ga–Zn cation from a Ga–Zn–Fe–Zn–Ga heteropentanuclear complex
Source: Chem Sci. 2026 Jul 31;17(39):18618–24. doi: 10.1039/d6sc04662b (PMC13507889; doi:10.1039/d6sc04662b)
Supplement: Supplementary file 1 [file SC-017-D6SC04662B-s001.pdf]

Supplementary Information (SI) for Chemical Science.
This journal is © The Royal Society of Chemistry 2026

Electronic Supplementary Information

Lewis Base-Induced Formation of a Ga–Zn Cation from a Ga–Zn–Fe–Zn–Ga Heteropentanuclear Complex

*Rajata Kumar Sahoo, Christoph Wölper, Gebhard Haberhauer, Stephan Schulz**

Contents

I. Experimental Section S3

II. Syntheses and Analytical Data of Complexes 1-7 S3-S11

III. Spectroscopic Data of Complexes 1-7 S12-S32

Figures S1-S5. ^{1}H, $^{13}C\{^{1}H\}$ NMR and IR spectra of **1** S12-S14

Figures S6-S12. ^{1}H, $^{13}C\{^{1}H\}$ NMR and IR spectra of **2** S14-S18

Figures S13-S15. ^{1}H, and $^{13}C\{^{1}H\}$ NMR spectra of **3** S19-S20

Figures S16-S22. ^{1}H, and $^{13}C\{^{1}H\}$ NMR spectra of **4** S21-S24

Figures S24-S25. ^{1}H, and $^{13}C\{^{1}H\}$ NMR spectra of **5** and **6** S25

Figures S26-S27. ^{1}H, and $^{13}C\{^{1}H\}$ NMR spectra of **5** and **7** S26

Figures S28-S32. ^{1}H, $^{13}C\{^{1}H\}$ NMR and IR spectra of **5** S27-S29

Figures S33-S35. ^{1}H, and $^{13}C\{^{1}H\}$ NMR spectra of **6** S29-S30

Figures S36-S38. ^{1}H, $^{13}C\{^{1}H\}$ NMR and IR spectra of **7** S31-S32

IV. Crystallographic Data of Complexes 1-5 S33-S41

Tables S1 and S2. Crystallographic details of compounds **1-5** S35-S36

Figures S39-S43. Molecular structures of **1-5** S37-S41

V. Computational Details S42-S61

Tables S3-S5. Calculated values of **2** and the anionic part of **3** S42-S43

Table S6. Analysis of the total bonding energy of **2** S43

Figures S44-S48. NOCV deformation densities of **2** S44-S48

Table S7. Analysis of the total bonding energy of the anionic part of **3** S49

Figures S49-S53. NOCV deformation densities of the anionic part of **3** S50-S54

Figures S54-S55. HOMO and LUMO of **2** and the anionic part of **3** S55-S56

VI. References S62-S63

I. Experimental Section

General procedure. All experiments and manipulations were carried out under a dry argon atmosphere using either standard *Schlenk* techniques or an MBraun LABmaster Pro glovebox. Toluene, and *n*-hexane were dried using an mBraun Solvent Purification System (SPS), degassed, and stored over 4Å molecular sieve. Benzene, THF, C_6D_6, Toluene-d_8 and THF-d_8 were dried by refluxing over Na/K alloy, distilled prior to use, and stored over 4Å molecular sieve under Ar atmosphere.

Commercial reagents were purchased from Aldrich, Acros, or Alfa-Aesar Chemical Co. and used as received. L^1Ga (L^1 = {HC(C(CH_3)N(Dipp))$_2$}, Dipp = 2,6-*i*Pr_2-C_6H_3),[1a] EtZnCl,[1b] $Na_2[Fe(CO)_4]$.*x*THF (*x* = ~1),[1c-d] and Cp*K[1e] were prepared according to literature procedures.

NMR spectra (^1H, and $^{13}C\{^1H\}$) were recorded on Jeol JNM-ECZL 400 MHz, Bruker Avance Neo 400 MHz and Bruker Avance III HD 600 MHz spectrometers. Chemical shifts δ are given in ppm and referenced to the solvent residual peak(s).[2] Elemental analyses were performed at the *Elementaranalyse Labor* of the University of Duisburg-Essen. IR spectra were recorded by a Bruker ALPHA-T FT-IR spectrometer equipped with a single-reflection ATR sampling module. Melting points were measured using a Thermo Scientific 9300 apparatus.

II. Syntheses and Analytical Data of Complexes 1-7

[L^1(Et)GaZn(THF)Cl]$_2$ (1). L^1Ga (1.00 g, 2.05 mmol) was dissolved in THF (10 mL) and cooled to –30 °C. EtZnCl (267 mg, 2.05 mmol) was dissolved in THF (5 mL), cooled to –30 °C, and added dropwise to the stirred L^1Ga solution. The reaction mixture was allowed to warm to room temperature and stirred for 5 min, yielding a pale-yellow solution. The solvent was then removed under vacuum, resulting in a pale yellow solid. Yield: 92% (1.30 g). M.p. 103 °C (decomp.). Single crystals suitable for X-ray diffraction analysis were grown by storing an THF solution of **1** at -3 °C for 24 hours, yielding pale yellow block-shaped crystals. Anal. calcd. (%) for $C_{70}H_{108}Cl_2Ga_2N_4O_2Zn_2$ (1372.49): C, 60.98; H, 7.90; N, 4.06. Found: C, 60.90; H, 8.07; N, 4.08.

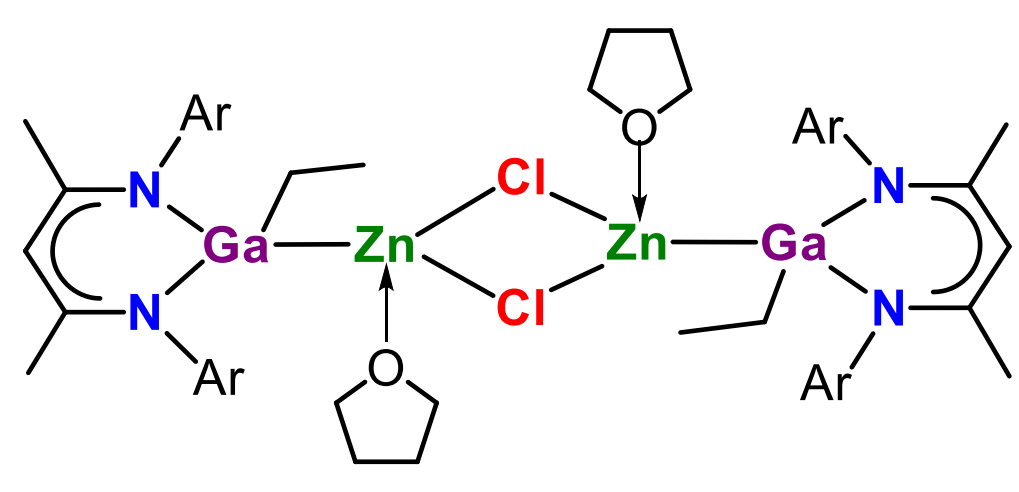

1

^1H NMR (400 MHz, C_6D_6, 298 K) δ = 0.41 (q, $^3J_{HH}$ = 8.0 Hz, 4H, CH_2CH$_3$), 0.84 (t, $^3J_{HH}$ = 8.0 Hz, 6H, CH$_2$CH_3), 1.12 (d, $^3J_{HH}$ = 6.8 Hz, 12H, CH(CH_3)$_2$), 1.36 (d, $^3J_{HH}$ = 6.9 Hz, 12H, CH(CH_3)$_2$), 1.39 – 1.44 (m, 20H, CH(CH_3)$_2$+THF-OCH$_2$CH_2), 1.55 (s, 12H, CCH_3), 1.60 (d, $^3J_{HH}$ = 6.9 Hz, 12H, CH(CH_3)$_2$), 3.43 – 3.54 (m, 8H, CH(CH$_3$)$_2$), 3.57 (t, $^3J_{HH}$ = 8.1 Hz, 8H, THF-OCH_2CH$_2$), 4.87 (s, 2H, CH), 7.09 – 7.13 (m, 12H, C$_6H_3$).

$^{13}C\{^1H\}$ NMR (100 MHz, C_6D_6, 298 K) δ = 5.0 (CH_2CH_3), 10.7 (CH_2CH_3), 22.8 ($CH(CH_3)_2$), 23.9 ($CH(CH_3)_2$), 24.0 ($CH(CH_3)_2$), 24.6 ($CH(CH_3)_2$), 25.4 (THF-CH_2), 27.5 (CCH_3), 28.5 ($CH(CH_3)_2$), 28.8 ($CH(CH_3)_2$), 67.5 (THF-OCH_2), 96.3 (CH), 123.7 (C_6H_3), 124.3 (C_6H_3), 126.4 (C_6H_3), 141.7 (C_6H_3), 143.0 (C_6H_3), 144.2 (C_6H_3), 168.3 (CN).

^1H NMR (400 MHz, THF-d_8, 298 K) δ = 0.25 (q, $^3J_{HH}$ = 8.1 Hz, 4H, CH_2CH_3), 0.84 (t, $^3J_{HH}$ = 8.0 Hz, 6H, CH_2CH_3), 1.21 – 1.19 (m, 24H, $CH(CH_3)_2$), 1.30 (d, $^3J_{HH}$ = 6.9 Hz, 12H, $CH(CH_3)_2$), 1.37 (d, $^3J_{HH}$ = 6.9 Hz, 12H, $CH(CH_3)_2$), 1.73 (s, 12H, CCH_3), 1.77 – 1.81 (m, 8H, THF-CH_2), 3.39 – 3.48 (m, 8H, $CH(CH_3)_2$), 3.62 – 3.65 (m, 8H, THF-OCH_2), 4.95 (s, 2H, CH), 7.15 – 7.18 (m, 12H, C_6H_3).

$^{13}C\{^1H\}$ NMR (100 MHz, THF-d_8, 298 K) δ = 9.4 (CH_2CH_3), 10.7 (CH_2CH_3), 22.7 ($CH(CH_3)_2$), 23.8 ($CH(CH_3)_2$), 24.0 ($CH(CH_3)_2$), 24.2 ($CH(CH_3)_2$), 25.3 (THF-CH_2), 25.5 (CCH_3), 27.6 ($CH(CH_3)_2$), 28.4 ($CH(CH_3)_2$), 67.2 (THF-OCH_2), 95.5 (CH), 123.4 (C_6H_3), 124.9 (C_6H_3), 125.7 (C_6H_3), 142.9 (C_6H_3), 143.6 (C_6H_3), 143.7 (C_6H_3), 167.3 (CN).

ATR-IR: ν 2976, 2926, 2868, 1551, 1518, 1433, 1394, 1313, 1259, 1174, 1019, 881, 792, 757, 481 cm^{-1}.

[L^1(Et)GaZn(THF)Fe(CO)$_4$Zn(THF)Ga(Et)L^1] (2). A mixture of [L^1(Et)GaZn(THF)Cl]$_2$ (**1**) (1.0 g, 0.73 mmol) and $Na_2[Fe(CO)_4]$.*x*THF (*x* = ~1) (207 mg, 0.73 mmol) was dissolved in ~15 mL of THF at –30 °C. The reaction mixture was warmed to room temperature and stirred for 20 min, affording a yellow solution. The solvent was removed under vacuum to give a yellow solid. This solid was dissolved in 15 mL of toluene and filtered, and the filtrate was concentrated under vacuum to yield the product as a yellow solid (86%, 836 mg). M.p. 170 °C (decomp.). Single crystals suitable for X-ray diffraction analysis were obtained by slow diffusion of *n*-hexane into a toluene solution of **2**, followed by storage at −30 °C for 24 h, giving pale yellow block-shaped crystals. Anal. calcd. (%) for $C_{66}H_{92}FeGa_2N_4O_4Zn_2$ (1326.36): C, 59.53; H, 6.96; N, 4.21. Found: C, 59.64; H, 7.05; N, 4.10.

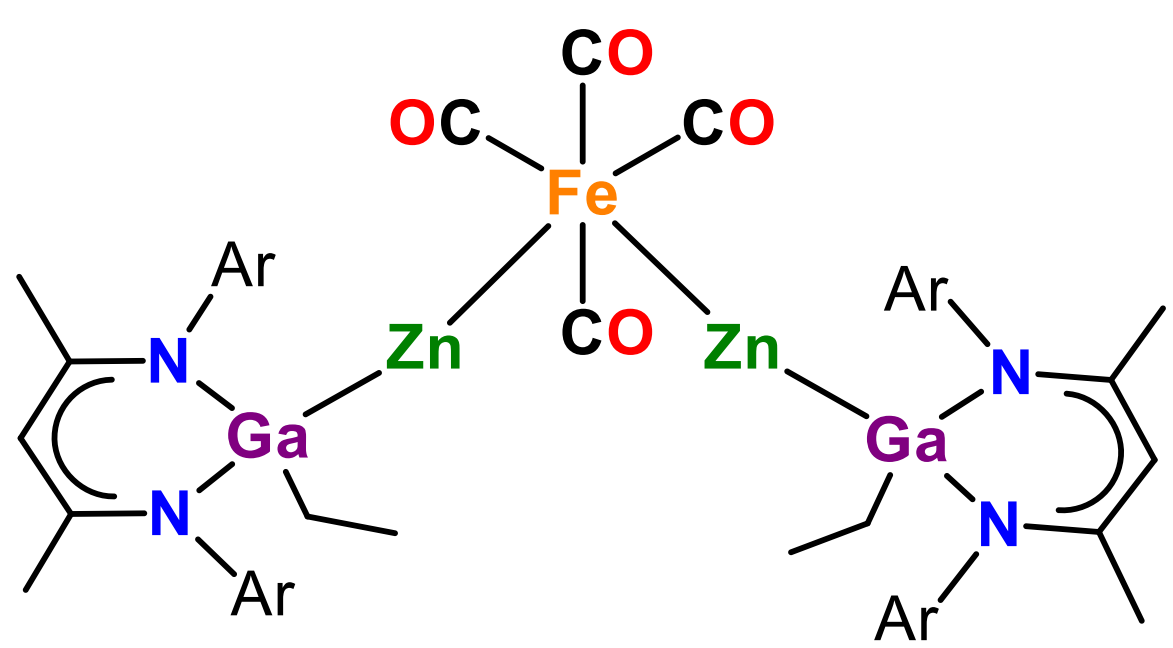

Ar = 2,6-*i*Pr$_2$-C_6H_3
2

^1H NMR (400 MHz, C_6D_6, 298 K) δ = 0.45 (q, $^3J_{HH}$ = 8.0 Hz, 4H, CH_2CH_3), 0.93 (t, $^3J_{HH}$ = 7.9 Hz, 6H, CH_2CH_3), 1.10 (d, $^3J_{HH}$ = 6.9 Hz, 12H, $CH(CH_3)_2$), 1.35 (d, $^3J_{HH}$ = 6.9 Hz, 6H, $CH(CH_3)_2$), 1.48 (d, $^3J_{HH}$ = 6.8 Hz, 12H, $CH(CH_3)_2$), 1.61 – 1.63 (m, 24H, $CH(CH_3)_2$ + CCH_3), 3.45 (sept, $^3J_{HH}$ = 7.0 Hz, 4H, $CH(CH_3)_2$), 3.56 (sept, $^3J_{HH}$ = 6.9 Hz, 4H, $CH(CH_3)_2$), 5.00 (s, 2H, CH), 7.08 – 7.12 (m, 12H, C_6H_3).

$^{13}C\{^1H\}$ NMR (100 MHz, C_6D_6, 298 K) δ = 4.86 (CH_2CH_3), 10.7 (CH_2CH_3), 22.7 ($CH(CH_3)_2$), 23.7 ($CH(CH_3)_2$), 24.0 ($CH(CH_3)_2$), 24.5 ($CH(CH_3)_2$), 27.0 (CCH_3), 28.4 ($CH(CH_3)_2$), 29.5 ($CH(CH_3)_2$), 95.8

(CH), 123.7 (C_6H_3), 124.3 (C_6H_3), 126.4 (C_6H_3), 141.6 (C_6H_3), 142.7 (C_6H_3), 144.1 (C_6H_3), 169.6 (CN), 212.6 (CO).

^{1}H NMR (400 MHz, toluene-d$_8$, 298 K) δ = 0.38 (q, $^3J_{HH}$ = 8.0 Hz, 4H, CH_2CH$_3$), 0.87 (t, $^3J_{HH}$ = 8.0 Hz, 6H, CH$_2$CH_3), 1.09 (d, $^3J_{HH}$ = 6.9 Hz, 12H, CH(CH_3)$_2$), 1.33 (d, $^3J_{HH}$ = 6.9 Hz, 12H, CH(CH_3)$_2$), 1.44 (d, $^3J_{HH}$ = 6.9 Hz, 12H, CH(CH_3)$_2$), 1.58 (d, $^3J_{HH}$ = 6.9 Hz, 12H, CH(CH_3)$_2$), 1.63 (s, 6H, CCH_3), 3.42 (sept, $^3J_{HH}$ = 7.0 Hz, 4H, CH(CH$_3$)$_2$), 3.51 (sept, $^3J_{HH}$ = 6.9 Hz, 4H, CH(CH$_3$)$_2$), 5.00 (s, 1H, CH), 7.06 – 7.08 (m, 10H, C$_6H_3$), 7.13 (s, 2H, C$_6H_3$).

^{13}C{^{1}H} NMR (100 MHz, toluene-d$_8$, 298 K) δ = 4.7 (CH$_2$CH$_3$), 10.6 (CH$_2$$CH_3$), 22.6 (CH($CH_3$)$_2$), 23.6 (CH($CH_3$)$_2$), 23.9 (CH($CH_3$)$_2$), 24.4 (CH($CH_3$)$_2$), 26.9 (CC$H_3$), 28.4 ($C$H(CH$_3$)$_2$), 29.4 ($C$H(CH$_3$)$_2$), 95.7 ($C$H), 123.6 ($C_6H_3$), 124.2 ($C_6H_3$), 126.3 ($C_6H_3$), 128.1 ($C_6H_3$), 141.5 ($C_6H_3$), 142.6 ($C_6H_3$), 144.0 ($C_6H_3$), 169.6 ($C$N), 212.5 ($C$O).

^{1}H NMR (400 MHz, THF-d$_8$, 298 K) δ = 0.14 (q, $^3J_{HH}$ = 8.0 Hz, 4H, CH_2CH$_3$), 0.62 (t, $^3J_{HH}$ = 8.0 Hz, 6H, CH$_2$CH_3), 1.13 (d, $^3J_{HH}$ = 6.9 Hz, 12H, CH(CH_3)$_2$), 1.31 (d, $^3J_{HH}$ = 6.9 Hz, 12H, CH(CH_3)$_2$), 1.34 (d, $^3J_{HH}$ = 6.9 Hz, 12H, CH(CH_3)$_2$), 1.41 (d, $^3J_{HH}$ = 6.9 Hz, 12H, CH(CH_3)$_2$), 1.77 (s, 12H, CCH_3), 3.29 – 3.37 (m, 8H, CH(CH$_3$)$_2$), 5.25 (s, 2H, CH), 7.17 – 7.21 (m, 10H, C$_6H_3$), 7.30 (s, 2H, C$_6H_3$).

^{13}C{^{1}H} NMR (100 MHz, THF-d$_8$, 298 K) δ = 4.4 (CH$_2$CH$_3$), 10.0 (CH$_2$$CH_3$), 22.3 (CH($CH_3$)$_2$), 23.1 (CH($CH_3$)$_2$), 23.5 (CH($CH_3$)$_2$), 24.0 (CH($CH_3$)$_2$), 26.4 (CC$H_3$), 28.3 ($C$H(CH$_3$)$_2$), 29.2 ($C$H(CH$_3$)$_2$), 95.6 ($C$H), 123.5 ($C_6H_3$), 124.0 ($C_6H_3$), 126.1 ($C_6H_3$), 127.9 ($C_6H_3$), 141.3 ($C_6H_3$), 142.6 ($C_6H_3$), 143.8 ($C_6H_3$), 169.5 ($C$N), 212.2 ($C$O).

ATR-IR: ν 2961, 2860, 2004, 1944, 1878, 1543, 1437, 1383, 1311, 1172, 1017, 937, 794, 670, 610, 535 cm^{-1}.

[L^1(Et)GaZnFe(CO)$_4$]$^-$ [(^{t}Bu-Py)$_3$Na]$^+$ (3). A mixture of [L^1(Et)GaZn(THF)Cl]$_2$ (**1**) (100 mg, 0.072 mmol), and Na$_2$[Fe(CO)$_4$].*x*THF (*x* = ~1) (41 mg, 0.145 mmol, 2 equivalent) was dissolved in THF (7 mL) at –30 °C, followed by the dropwise addition of a solution of 4-tert-butylpyridine (63.8 µL, 0.435 mmol, 6 equivalent) in THF (3 mL) at the same temperature. The reaction mixture was allowed to warm to room temperature and stirred for 20 min, affording a pale orange solution. Removal of the solvent under vacuum yielded a pale orange solid, which was subsequently redissolved in THF and filtered. The filtrate was layered with *n*-hexane and stored at room temperature. Yellow block-shaped crystals of **3** suitable for X-ray diffraction were obtained within 5 days. Yield: 40% (68 mg). M.p. 240 °C (decomp.).

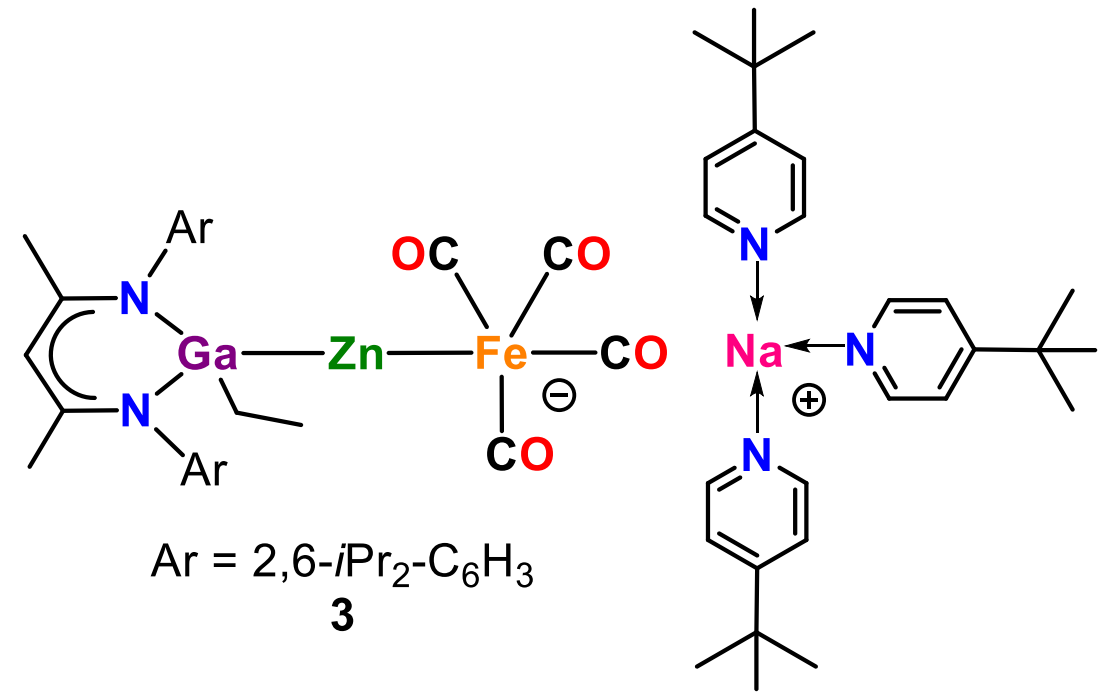

3

Anal. calcd. (%) for $C_{62}H_{85}FeGaN_5NaO_4Zn$ (1175.44): C, 63.20; H, 7.27; N, 5.94. Found: C, 64.70; H, 7.64; N, 5.48.

^{1}H NMR (400 MHz, THF-d$_8$, 298 K) δ = -0.01 (q, $^3J_{HH}$ = 8.0 Hz, 2H, CH_2CH$_3$), 0.57 (t, $^3J_{HH}$ = 8.0 Hz, 3H, CH$_2$CH_3), 1.16 (d, $^3J_{HH}$ = 7.0 Hz, 6H, CH(CH_3)$_2$), 1.34 (d, $^3J_{HH}$ = 6.9 Hz, 6H, CH(CH_3)$_2$), 1.34 – 1.36 (m, 33H, C(CH_3)$_3$ + CH(CH_3)$_2$), 1.42 (d, $^3J_{HH}$ = 6.9 Hz, 6H, CH(CH_3)$_2$), 1.73 (s, 6H, CCH_3), 3.48 – 3.54 (m, 2H, CH(CH$_3$)$_2$), 3.55 – 3.61 (m, 2H, CH(CH$_3$)$_2$), 5.07 (s, 1H, CH), 7.13 (s, 1H, C$_6H_3$), 7.15 (d, $^3J_{HH}$ = 6.9 Hz, 3H, C$_6H_3$), 7.17 – 7.20 (m, 2H, C$_6H_3$), 7.34 (d, $^3J_{HH}$ = 6.9 Hz, 6H, N(CHCH)$_2$CC(CH$_3$)$_3$), 8.48 (d, $^3J_{HH}$ = 6.9 Hz, 6H, N(CHCH)$_2$CC(CH$_3$)$_3$).

^{13}C{^{1}H} NMR (100 MHz, THF-d$_8$, 298 K) δ = 5.0 (CH$_2$CH$_3$), 10.4 (CH$_2$$CH_3$), 22.4 (CH($CH_3$)$_2$), 23.0 (CH($CH_3$)$_2$), 23.7 (CH($CH_3$)$_2$), 24.2 (CH($CH_3$)$_2$), 26.4 (CC$H_3$), 28.1 ($C$H(CH$_3$)$_2$), 28.6 ($C$H(CH$_3$)$_2$), 29.8 C($CH_3$)$_3$, 34.2 C(CH$_3$)$_3$, 95.8 (CH), 120.2 N(CHCH)$_2$CC(CH$_3$)$_3$), 122.8 (C_6H$_3$), 123.8 (C_6H$_3$), 125.2 (C_6H$_3$), 142.6 N(CHCH)$_2$CC(CH$_3$)$_3$), 145.1 (C_6H$_3$), 149.6 (C_6H$_3$), 159.0 N(CHCH)$_2$$C$C(CH$_3$)$_3$), 167.9 ($C$N), 222.9 ($C$O), 224.1 ($C$O).

ATR-IR: ν 2959, 2866, 1950, 1772, 1607, 1551, 1385, 1313, 1174, 1007, 825, 759, 624, 568, 531 cm^{-1}.

[L^1(Et)GaZnFe(CO)$_4$]$^-$ [(DMAP)$_3$ZnGa(Et)L^1]$^+$ (4). DMAP (206 mg, 1.69 mmol) was dissolved in toluene (5 mL) and added to a stirred solution of **2** (750 mg, 0.56 mmol) in toluene (10 mL) at –30 °C in a Schlenk tube, immediately affording a pale orange solution. The reaction mixture was allowed to warm to room temperature and stirred for 1 h, followed by the solvent was removed under vacuum to yield the product as a pale orange solid (85%, 813 mg). M.p. 175 °C (decomp.). Single crystals of **4** suitable for X-ray diffraction analysis were grown by layering *n*-hexane onto a THF solution and keeping the sample at −30 °C for 24 h, giving yellowish orange block-shaped crystals. Anal. calcd. (%) for $C_{87}H_{122}FeGa_2N_{10}O_4Zn_2$ (1692.61): C, 61.54; H, 7.24; N, 8.25. Found: C, 61.34; H, 7.14; N, 8.04.

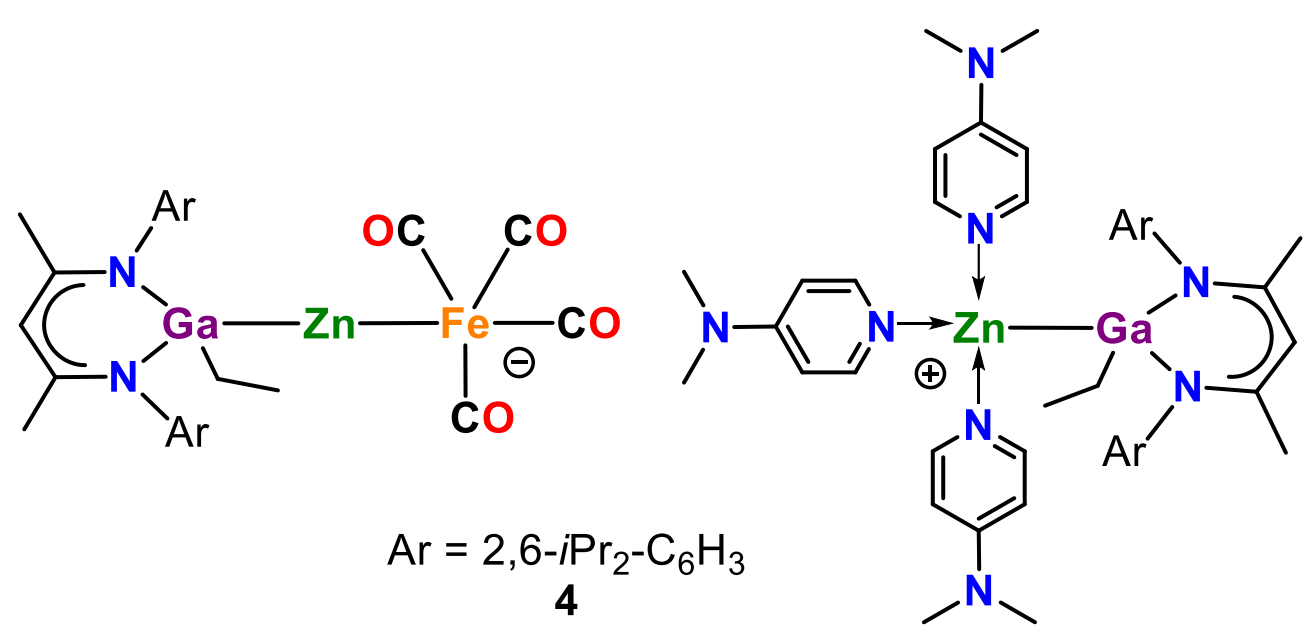

Ar = 2,6-*i*Pr$_2$-C$_6$H$_3$
4

^{1}H NMR (400 MHz, C$_6$D$_6$, 298 K) δ = 0.42 (q, $^3J_{HH}$ = 8.0 Hz, 2H, CH_2CH$_3$), 0.98 – 1.01 (m, 9H, CH(CH_3)$_2$ + CH$_2$CH_3), 1.13 (q, $^3J_{HH}$ = 8.1 Hz, 2H, CH_2CH$_3$), 1.20 (d, $^3J_{HH}$ = 6.9 Hz, 12H, CH(CH_3)$_2$), 1.31 (d, $^3J_{HH}$ = 6.8 Hz, 6H, CH(CH_3)$_2$), 1.36 (d, $^3J_{HH}$ = 6.7 Hz, 6H, CH(CH_3)$_2$), 1.43 (d, $^3J_{HH}$ = 6.9 Hz, 6H, CH(CH_3)$_2$), 1.63 (d, $^3J_{HH}$ = 6.9 Hz, 6H, CH(CH_3)$_2$), 1.67 (s, 6H, CCH_3), 1.69 (s, 6H, CCH_3), 1.77 (d, $^3J_{HH}$ = 6.9 Hz, 6H, CH(CH_3)$_2$), 1.96 (t, $^3J_{HH}$ = 8.0 Hz, 3H, CH$_2$CH_3), 2.33 (s, 18H, N(CHCH)$_2$CN(CH_3)$_2$), 3.59 (sept, $^3J_{HH}$ = 7.1 Hz, 2H, CH(CH$_3$)$_2$), 3.71 (sept, $^3J_{HH}$ = 7.0 Hz, 2H, CH(CH$_3$)$_2$), 3.85 (sept, $^3J_{HH}$ = 7.0 Hz, 2H, CH(CH$_3$)$_2$),

3.99 (sept, $^3J_{HH}$ = 7.1 Hz, 2H, C*H*(CH_3)$_2$), 4.90 (s, 1H, C*H*), 4.99 (s, 1H, C*H*), 6.21 (d, $^3J_{HH}$ = 6.8 Hz, 6H, N(CHC*H*)$_2$CN(CH_3)$_2$), 7.12 – 7.15 (m, 6H, C_6*H*$_3$), 7.21 – 7.25 (m, 6H, C_6*H*$_3$), 7.46 (s, 6H, N(C*H*CH)$_2$CN(CH_3)$_2$).

^{13}C{^{1}H} NMR (100 MHz, C_6D_6, 298 K) δ = 5.90 (*C*H_2CH_3), 11.60 (*C*H_2CH_3), 14.36 (CH_2*C*H_3), 19.82 (CH_2*C*H_3), 23.2 (CH(*C*H_3)$_2$), 24.1 (CH(*C*H_3)$_2$), 24.2 (CH(*C*H_3)$_2$), 24.6 (CH(*C*H_3)$_2$), 24.7 (CH(*C*H_3)$_2$), 24.7 (CH(*C*H_3)$_2$), 24.8 (CH(*C*H_3)$_2$), 25.0 (CH(*C*H_3)$_2$), 25.5 (CC*H*$_3$), 26.3 (CC*H*$_3$), 27.5 (*C*H(CH_3)$_2$), 28.6 (*C*H(CH_3)$_2$), 28.7 (*C*H(CH_3)$_2$), 29.4 (*C*H(CH_3)$_2$), 38.4 N(CHCH)$_2$CN(*C*H_3)$_2$), 96.7 (*C*H), 99.0 (*C*H), 107.5 N(CH*C*H)$_2$CN(CH_3)$_2$), 123.5 (*C*$_6H_3$), 124.4 (*C*$_6H_3$), 124.5 (*C*$_6H_3$), 124.9 (*C*$_6H_3$), 125.9 (*C*$_6H_3$), 126.1 (*C*$_6H_3$), 128.3 (*C*$_6H_3$), 128.5 (*C*$_6H_3$), 143.2 (*C*$_6H_3$), 143.3 (*C*$_6H_3$), 143.7 (*C*$_6H_3$), 145.6 (*C*$_6H_3$), 145.8 (*C*$_6H_3$), 146.1 (*C*$_6H_3$), 148.6 N(*C*HCH)$_2$CN(CH_3)$_2$), 154.8 N(CHCH)$_2$*C*N(CH_3)$_2$), 167.9 (*C*N), 168.4 (*C*N), 225.0 (*C*O).

^{1}H NMR (400 MHz, toluene-d$_8$, 298 K) δ = 0.32 (q, $^3J_{HH}$ = 8.0 Hz, 2H, C*H*$_2CH_3$), 0.90 (t, $^3J_{HH}$ = 8.1 Hz, 3H, CH_2C*H*$_3$), 0.96 (d, $^3J_{HH}$ = 7.0 Hz, 6H, CH(C*H*$_3$)$_2$), 1.04 (q, $^3J_{HH}$ = 8.1 Hz, 2H, C*H*$_2CH_3$), 1.15 – 1.18 (m, 12H, CH(C*H*$_3$)$_2$), 1.27 (d, $^3J_{HH}$ = 6.9 Hz, 6H, CH(C*H*$_3$)$_2$), 1.35 (d, $^3J_{HH}$ = 6.9 Hz, 6H, CH(C*H*$_3$)$_2$), 1.41 (d, $^3J_{HH}$ = 7.0 Hz, 6H, CH(C*H*$_3$)$_2$), 1.65 – 1.68 (m, 18H, CH(C*H*$_3$)$_2$ + 2 CC*H*$_3$), 1.86 (t, $^3J_{HH}$ = 8.0 Hz, 3H, CH_2C*H*$_3$), 2.38 (s, 18H, N(CHCH)$_2$CN(C*H*$_3$)$_2$), 3.56 (sept, $^3J_{HH}$ = 7.0 Hz, 2H, C*H*(CH_3)$_2$), 3.68 (sept, $^3J_{HH}$ = 7.1 Hz, 2H, C*H*(CH_3)$_2$), 3.81 (sept, $^3J_{HH}$ = 7.0 Hz, 2H, C*H*(CH_3)$_2$), 3.90 (sept, $^3J_{HH}$ = 7.1 Hz, 2H, C*H*(CH_3)$_2$), 4.86 (s, 1H, C*H*), 4.94 (s, 1H, C*H*), 6.17 (d, $^3J_{HH}$ = 6.6 Hz, 6H, N(CHC*H*)$_2$CN(CH_3)$_2$), 7.10 – 7.12 (m, 6H, C_6*H*$_3$), 7.17 – 7.19 (m, 2H, C_6*H*$_3$), 7.21 – 7.24 (m, 2H, C_6*H*$_3$), 7.55 (s, 6H, N(C*H*CH)$_2$CN(CH_3)$_2$).

^{13}C{^{1}H} NMR (100 MHz, toluene-d$_8$, 298 K) δ = 5.5 (*C*H_2CH_3), 11.1 (*C*H_2CH_3), 13.9 (CH_2*C*H_3), 13.9 (CH_2*C*H_3), 22.8 (CH(*C*H_3)$_2$), 23.7 (CH(*C*H_3)$_2$), 23.8 (CH(*C*H_3)$_2$), 24.2 (CH(*C*H_3)$_2$), 24.3 (CH(*C*H_3)$_2$), 24.3 (CH(*C*H_3)$_2$), 24.5 (CH(*C*H_3)$_2$), 24.7 (CH(*C*H_3)$_2$), 26.0 (CC*H*$_3$), 27.0 (CC*H*$_3$), 27.1 (*C*H(CH_3)$_2$), 28.3 (*C*H(CH_3)$_2$), 28.4 (*C*H(CH_3)$_2$), 29.0 (*C*H(CH_3)$_2$), 38.1 N(CHCH)$_2$CN(*C*H_3)$_2$), 96.2 (*C*H), 98.6 (*C*H), 107.2 N(CH*C*H)$_2$CN(CH_3)$_2$), 123.1 (*C*$_6H_3$), 124.0 (*C*$_6H_3$), 124.1 (*C*$_6H_3$), 124.5 (*C*$_6H_3$), 125.5 (*C*$_6H_3$), 125.7 (*C*$_6H_3$), 142.8 (*C*$_6H_3$), 142.9 (*C*$_6H_3$), 143.3 (*C*$_6H_3$), 145.3 (*C*$_6H_3$), 145.4 (*C*$_6H_3$), 145.7 (*C*$_6H_3$), 148.0 N(*C*HCH)$_2$CN(CH_3)$_2$), 154.7 N(CHCH)$_2$*C*N(CH_3)$_2$), 167.6 (*C*N), 168.0 (*C*N), 224.5 (*C*O).

^{1}H NMR (400 MHz, THF-d$_8$, 298 K) δ = 0.00 (q, $^3J_{HH}$ = 7.9 Hz, 2H, C*H*$_2CH_3$), 0.60 (t, $^3J_{HH}$ = 8.0 Hz, 3H, CH_2C*H*$_3$), 0.71 (q, $^3J_{HH}$ = 7.8 Hz, 2H, C*H*$_2CH_3$), 0.91 (d, $^3J_{HH}$ = 7.0 Hz, 6H, CH(C*H*$_3$)$_2$), 1.09 (d, $^3J_{HH}$ = 7.1 Hz, 6H, CH(C*H*$_3$)$_2$), 1.18 (d, $^3J_{HH}$ = 7.0 Hz, 12H, CH(C*H*$_3$)$_2$), 1.33 (d, $^3J_{HH}$ = 7.1 Hz, 6H, CH(C*H*$_3$)$_2$), 1.35 (d, $^3J_{HH}$ = 6.9 Hz, 6H, CH(C*H*$_3$)$_2$), 1.36 (d, $^3J_{HH}$ = 7.0 Hz, 6H, CH(C*H*$_3$)$_2$), 1.43 (d, $^3J_{HH}$ = 6.9 Hz, 6H, CH(C*H*$_3$)$_2$), 1.50 (t, $^3J_{HH}$ = 8.1 Hz, 3H, CH_2C*H*$_3$), 1.73 (s, 6H, CC*H*$_3$), 1.79 (s, 6H, CC*H*$_3$), 3.05 (s, 18H, N(CHCH)$_2$CN(C*H*$_3$)$_2$), 3.50 (sept, $^3J_{HH}$ = 7.0 Hz, 2H, C*H*(CH_3)$_2$), 3.57 (sept, $^3J_{HH}$ = 7.1 Hz, 2H, C*H*(CH_3)$_2$),

3.63 (sept, $^3J_{HH}$ = 7.0 Hz, 4H, C*H*(CH$_3$)$_2$), 5.03 (s, 1H, C*H*), 5.06 (s, 1H, C*H*), 6.52 (d, $^3J_{HH}$ = 6.9 Hz, 6H, N(CHC*H*)$_2$CN(CH$_3$)$_2$), 7.14 – 7.20 (m, 6H, C$_6$*H*$_3$), 7.22 – 7.24 (m, 2H, C$_6$*H*$_3$), 7.33 – 7.35 (m, 2H, C$_6$*H*$_3$), 7.38 – 7.40 (m, 2H, C$_6$*H*$_3$), 7.61 (s, 6H, N(C*H*CH)$_2$CN(CH$_3$)$_2$).

^{13}C{^{1}H} NMR (100 MHz, THF-d$_8$, 298 K) δ = 6.1 (*C*H$_2$CH$_3$), 11.4 (*C*H$_2$CH$_3$), 14.4 (CH$_2$*C*H$_3$), 19.6 CH$_2$*C*H$_3$), 23.4 (CH(*C*H$_3$)$_2$), 23.5 (CH(*C*H$_3$)$_2$), 24.1 (CH(*C*H$_3$)$_2$), 24.3 (CH(*C*H$_3$)$_2$), 24.8 (CH(*C*H$_3$)$_2$), 24.8 (CH(*C*H$_3$)$_2$), 24.9 (CH(*C*H$_3$)$_2$), 25.5 (CH(*C*H$_3$)$_2$), 25.6 (CH(*C*H$_3$)$_2$), 25.8 (CH(*C*H$_3$)$_2$), 26.4 (CC*H*$_3$), 27.4 (CC*H*$_3$), 27.9 (*C*H(CH$_3$)$_2$), 29.1 (*C*H(CH$_3$)$_2$), 29.6 (*C*H(CH$_3$)$_2$), 32.5 (*C*H(CH$_3$)$_2$), 39.1 N(CHCH)$_2$CN(*C*H$_3$)$_2$), 96.8 (*C*H), 99.3 (*C*H), 107.9 N(CH*C*H)$_2$CN(CH$_3$)$_2$), 123.7 (*C*$_6$H$_3$), 124.8 (*C*$_6$H$_3$), 125.0 (*C*$_6$H$_3$), 125.6 (*C*$_6$H$_3$), 126.1 (*C*$_6$H$_3$), 126.8 (*C*$_6$H$_3$), 143.6 (*C*$_6$H$_3$), 143.8 (*C*$_6$H$_3$), 144.1 (*C*$_6$H$_3$), 146.1 (*C*$_6$H$_3$), 146.3 (*C*$_6$H$_3$), 146.4 (*C*$_6$H$_3$), 148.8 N(*C*HCH)$_2$CN(CH$_3$)$_2$), 155.9 N(CHCH)$_2$*C*N(CH$_3$)$_2$), 168.6 (*C*N), 168.7 (*C*N), 224.8 (*C*O).

ATR-IR: ν 2955, 2862, 1946, 1811, 1615, 1534, 1439, 1385, 1315, 1226, 1172, 1061, 1009, 794, 624, 527 cm^{-1}.

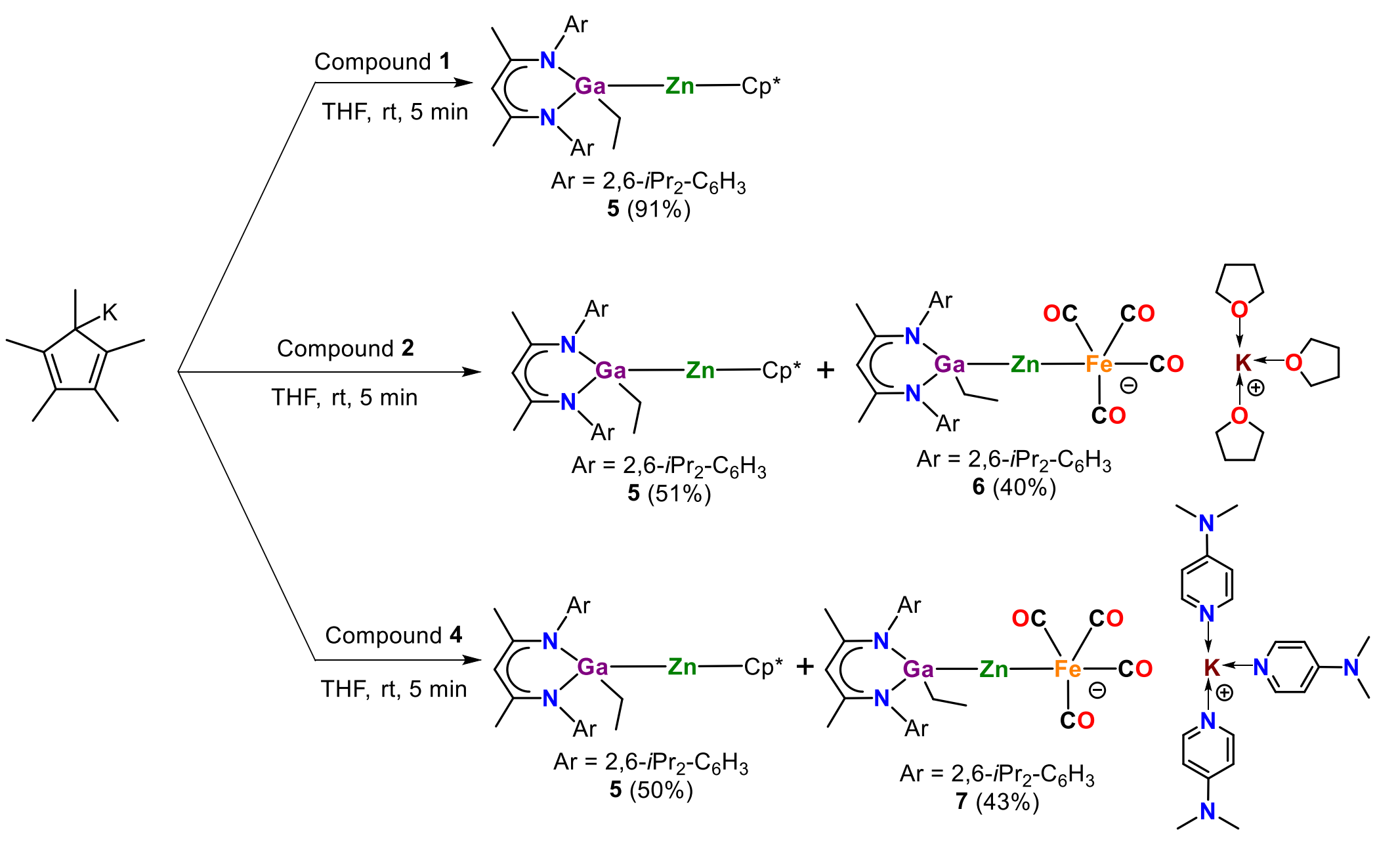

Synthesis of compounds 5 and 6: A mixture of compound **2** (200 mg, 0.15 mmol) and Cp*K (26 mg, 0.15 mmol) was dissolved in ~10 mL of THF at –30 °C. The reaction mixture was warmed to room temperature and stirred for 5 min, affording a pale-yellow solution. The solvent was removed under vacuum to yield a pale-yellow solid. The resulting solid was washed three times with *n*-hexane (~8 mL each) and dried under vacuum to afford compound **6** as a yellow solid in 40% yield (60 mg). M.p. 235 °C (decomp.). Anal. calcd. (%) for $C_{47}H_{70}FeGaKN_2O_7Zn$ (1002.27): C, 56.16; H, 7.02; N, 2.79. Found: C, 55.49; H, 7.42; N, 2.83.

The combined mother liquor was subsequently dried and extracted with n-hexane to give compound **5** as a pale-yellow solid in 51% yield (55 mg). Single crystals suitable for X-ray diffraction analysis were obtained by storing an *n*-hexane solution of **5** at −30 °C for 24 h, yielding colourless block-shaped crystals.

Synthesis of compounds 5 and 7: A mixture of compound **4** (200 mg, 0.12 mmol) and Cp*K (21 mg, 0.12 mmol) was dissolved in ~10 mL of THF at –30 °C. The reaction mixture was warmed to room temperature and stirred for 5 min, affording a pale-yellow solution. The solvent was removed under vacuum to yield a pale-yellow solid. The resulting solid was washed three times with *n*-hexane (~8 mL each) and dried under vacuum to afford compound **7** as a yellow solid in 43% yield (75 mg). M.p. 237 °C (decomp.). Anal. calcd. (%) for $C_{56}H_{76}FeGaKN_8O_4Zn$ (1152.35): C, 58.22; H, 6.63; N, 9.70. Found: C, 59.79; H, 6.87; N, 8.17.

The combined mother liquor was subsequently dried and extracted with *n*-hexane to give compound **5** as a pale-yellow solid in 50% yield (53 mg).

Alternative way to synthesis of **[L^1(Et)GaZnCp*] (5):** A mixture of [L^1(Et)GaZn(THF)Cl]$_2$ (**1**) (500 mg, 0.36 mmol) and Cp*K (126 mg, 0.73 mmol) was dissolved in ~15 mL of THF at –30 °C. The reaction mixture was warmed to room temperature and stirred for 5 min, affording a pale-yellow solution. The solvent was removed under vacuum to give a pale-yellow solid. The residue was dissolved in n-hexane (20 mL) and filtered. The filtrate was concentrated under reduced pressure to afford compound **5** as a pale-yellow solid in 91% yield (475 mg). M.p. 165 °C (decomp.). Single crystals suitable for X-ray diffraction analysis were obtained by storing an *n*-hexane solution of **5** at −30 °C for 24 h, giving colourless block-shaped crystals. Anal. calcd. (%) for $C_{41}H_{61}GaN_2Zn$ (714.34): C, 68.68; H, 8.58; N, 3.91. Found: C, 68.56; H, 8.65; N, 3.78.

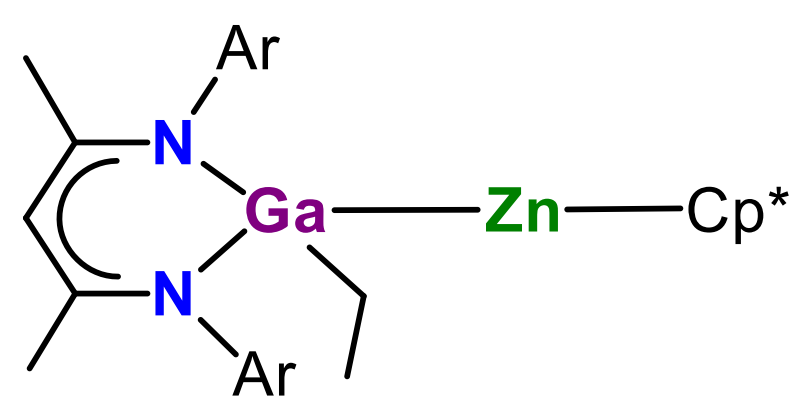

Ar = 2,6-*i*Pr$_2$-C$_6$H$_3$
5

^{1}H NMR (400 MHz, C_6D_6, 298 K) δ = 0.79 (q, $^3J_{HH}$ = 8.0 Hz, 2H, CH_2CH$_3$), 1.14 (d, $^3J_{HH}$ = 6.9 Hz, 6H, CH(CH_3)$_2$), 1.23 (d, $^3J_{HH}$ = 6.9 Hz, 6H, CH(CH_3)$_2$), 1.36 (d, $^3J_{HH}$ = 6.8 Hz, 6H, CH(CH_3)$_2$), 1.40 (d, $^3J_{HH}$ = 6.8 Hz, 6H, CH(CH_3)$_2$), 1.54 (s, 6H, CCH_3), 1.58 (t, $^3J_{HH}$ = 7.9 Hz, 3H, CH$_2$CH_3), 1.85 (s, 15H, C$_5$*Me*$_5$), 3.36 (sept, $^3J_{HH}$ = 7.0 Hz, 2H, C*H*(CH$_3$)$_2$), 3.46 (sept, $^3J_{HH}$ = 6.9 Hz, 2H, C*H*(CH$_3$)$_2$), 4.67 (s, 1H, C*H*), 7.12 – 7.14 (m, 6H, C$_6$*H*$_3$).

^{13}C{^{1}H} NMR (100 MHz, C_6D_6, 298 K) δ = 10.0 (C$_5$*Me*$_5$), 12.4 (*C*H$_2$CH$_3$), 14.3 (CH$_2$*C*H$_3$), 23.5 (CH(*C*H$_3$)$_2$), 24.2 (CH(*C*H$_3$)$_2$), 24.4 (CH(*C*H$_3$)$_2$), 24.9 (CH(*C*H$_3$)$_2$), 25.9 (CC*H*$_3$), 27.1 (*C*H(CH$_3$)$_2$), 28.6 (*C*H(CH$_3$)$_2$), 96.5 (*C*H), 108.1 (C$_5$*Me*$_5$), 123.8 (*C*$_6$H$_3$), 124.0 (*C*$_6$H$_3$), 126.1 (*C*$_6$H$_3$), 142.3 (*C*$_6$H$_3$), 143.0 (*C*$_6$H$_3$), 144.8 (*C*$_6$H$_3$), 167.8 (*C*N).

^{1}H NMR (400 MHz, THF-d$_8$, 298 K) δ = 0.44 (q, $^{3}J_{HH}$ = 7.9 Hz, 2H, C*H*$_2$CH$_3$), 1.12 (d, $^{3}J_{HH}$ = 6.9 Hz, 6H, CH(C*H*$_3$)$_2$), 1.21 – 1.23 (m, 15H, CH(C*H*$_3$)$_2$ + CH$_2$C*H*$_3$), 1.34 (d, $^{3}J_{HH}$ = 6.9 Hz, 6H, CH(C*H*$_3$)$_2$), 1.61 (s, 15H, C$_5$*Me*$_5$), 1.71 (s, 6H, CC*H*$_3$), 3.32 (sept, $^{3}J_{HH}$ = 7.0 Hz, 4H, C*H*(CH$_3$)$_2$), 4.88 (s, 1H, C*H*), 7.17 – 7.20 (m, 6H, C$_6$*H*$_3$).

$^{13}C\{^{1}H\}$ NMR (100 MHz, THF-d$_8$, 298 K) δ = 9.4 (C$_5$*Me*$_5$), 11.7 (*C*H$_2$CH$_3$), 14.0 (CH$_2$*C*H$_3$), 23.2 (CH(*C*H$_3$)$_2$), 23.8 (CH(*C*H$_3$)$_2$), 24.0 (CH(*C*H$_3$)$_2$), 24.7 (CH(*C*H$_3$)$_2$), 25.5 (CC*H*$_3$), 27.0 (*C*H(CH$_3$)$_2$), 28.5 (*C*H(CH$_3$)$_2$), 96.4 (*C*H), 107.7 (C$_5$*Me*$_5$), 123.8 (*C*$_6$H$_3$), 123.8 (*C*$_6$H$_3$), 125.9 (*C*$_6$H$_3$), 142.3 (*C*$_6$H$_3$), 143.0 (*C*$_6$H$_3$), 144.7 (*C*$_6$H$_3$), 168.0 (*C*N).

ATR-IR: ν 2964, 2926, 2856, 1551, 1518, 1437, 1385, 1323, 1313, 1174, 1100, 1019, 934, 858, 794, 757, 635, 502 cm^{-1}.

[L^1(Et)GaZnFe(CO)$_4$]$^-$ [(THF)$_3$K]$^+$ (6). ^{1}H NMR (400 MHz, THF-d$_8$, 298 K) δ = 0.00 (q, $^{3}J_{HH}$ = 8.0 Hz, 2H, C*H*$_2$CH$_3$), 0.50 (t, $^{3}J_{HH}$ = 8.0 Hz, 3H, CH$_2$C*H*$_3$), 1.16 (d, $^{3}J_{HH}$ = 6.9 Hz, 6H, CH(C*H*$_3$)$_2$), 1.34 (d, $^{3}J_{HH}$ = 6.9 Hz, 6H, CH(C*H*$_3$)$_2$), 1.36 (d, $^{3}J_{HH}$ = 6.9 Hz, 6H, CH(C*H*$_3$)$_2$), 1.42 (d, $^{3}J_{HH}$ = 6.9 Hz, 6H, CH(C*H*$_3$)$_2$), 1.73 (s, 6H, CC*H*$_3$), 1.77 (brs, 12H, THF-OCH$_2$C*H*$_2$), 3.48 – 3.54 (m, 2H, C*H*(CH$_3$)$_2$), 3.55 – 3.60 (m, 2H, C*H*(CH$_3$)$_2$), 3.63 (brs, 12H, THF-OC*H*$_2$CH$_2$), 5.07 (s, 1H, C*H*), 7.15 (d, $^{3}J_{HH}$ = 7.0 Hz, 3H, C$_6$*H*$_3$), 7.18 – 7.20 (m, 3H, C$_6$*H*$_3$).

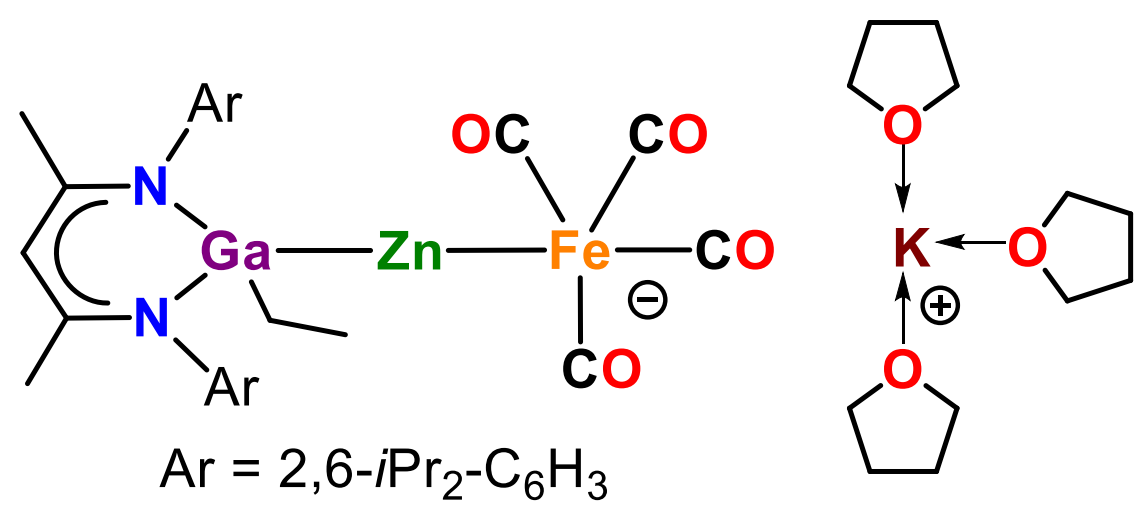

Ar = 2,6-*i*Pr$_2$-C$_6$H$_3$
6

$^{13}C\{^{1}H\}$ NMR (100 MHz, THF-d$_8$, 298 K) δ = 4.9 (*C*H$_2$CH$_3$), 10.4 (CH$_2$*C*H$_3$), 22.4 (CH(*C*H$_3$)$_2$), 23.0 (CH(*C*H$_3$)$_2$), 23.7 (CH(*C*H$_3$)$_2$), 25.4 (THF-OCH$_2$*C*H$_2$), 26.4 (CC*H*$_3$), 28.1 (*C*H(CH$_3$)$_2$), 28.6 (*C*H(CH$_3$)$_2$), 66.9 (THF-O*C*H$_2$CH$_2$), 95.8 (*C*H), 122.8 (*C*$_6$H$_3$), 123.8 (*C*$_6$H$_3$), 125.2 (*C*$_6$H$_3$), 142.6 (*C*$_6$H$_3$), 142.6 (*C*$_6$H$_3$), 145.1 (*C*$_6$H$_3$), 167.9 (*C*N), 224.0 (*C*O).

ATR-IR: ν 2961, 2862, 1944, 1855, 1799, 1549, 1435, 1389, 1315, 1253, 1172, 1015, 934, 794, 626, 529 cm^{-1}.

[L^1(Et)GaZnFe(CO)$_4$]$^-$ [(DMAP)$_3$K]$^+$ (7). ^{1}H NMR (400 MHz, THF-d$_8$, 298 K) δ = -0.05 (q, $^3J_{HH}$ = 8.0 Hz, 2H, CH_2CH$_3$), 0.54 (t, $^3J_{HH}$ = 8.0 Hz, 3H, CH$_2$CH_3), 1.11 (d, $^3J_{HH}$ = 6.9 Hz, 6H, CH(CH_3)$_2$), 1.29 (d, $^3J_{HH}$ = 6.9 Hz, 6H, CH(CH_3)$_2$), 1.31 (d, $^3J_{HH}$ = 6.9 Hz, 6H, CH(CH_3)$_2$), 1.37 (d, $^3J_{HH}$ = 6.9 Hz, 6H, CH(CH_3)$_2$), 1.68 (s, 6H, CCH_3), 2.96 (s, 18H, N(CHCH)$_2$CN(CH_3)$_2$), 3.44 – 3.49 (m, 2H, CH(CH$_3$)$_2$), 3.51 – 3.54 (m, 2H, CH(CH$_3$)$_2$), 5.02 (s, 1H, CH), 6.48 (d, $^3J_{HH}$ = 6.9 Hz, 6H, N(CHCH)$_2$CN(CH$_3$)$_2$), 7.10 (d, $^3J_{HH}$ = 7.0 Hz, 3H, C$_6H_3$), 7.14 (d, $^3J_{HH}$ = 7.1 Hz, 2H, C$_6H_3$), 7.19 (d, $^3J_{HH}$ = 6.9 Hz, 1H, C$_6H_3$), 7.99 (s, 6H, N(CHCH)$_2$CN(CH$_3$)$_2$).

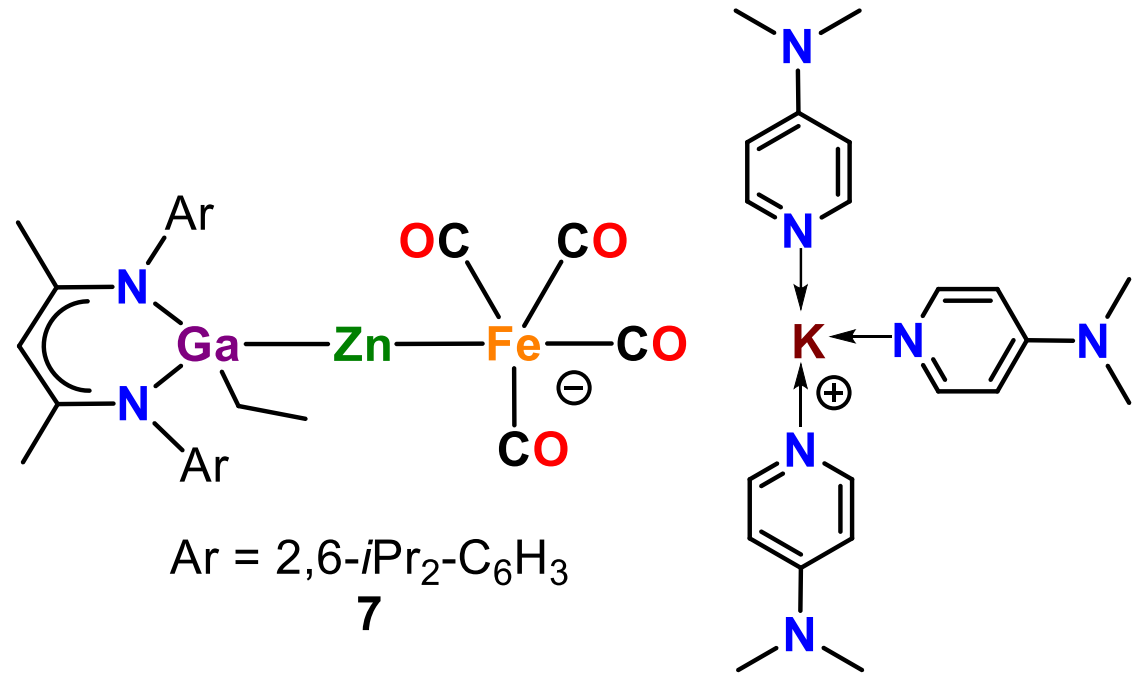

Ar = 2,6-iPr$_2$-C$_6$H$_3$
7

^{13}C{^{1}H} NMR (100 MHz, THF-d$_8$, 298 K) δ = 5.0 (CH$_2$CH$_3$), 9.3 (CH$_2$$CH_3$), 10.4 (CH$_2$$CH_3$), 22.4 (CH($CH_3$)$_2$), 23.0 (CH($CH_3$)$_2$), 23.7 (CH($CH_3$)$_2$), 24.2 (CH($CH_3$)$_2$), 26.4 (CC$H_3$), 28.1 ($C$H(CH$_3$)$_2$), 28.6 ($C$H(CH$_3$)$_2$), 38.0 N(CHCH)$_2$CN($CH_3$)$_2$), 95.8 ($C$H), 106.4 N(CH$C$H)$_2$CN(CH$_3$)$_2$), 122.8 ($C_6H_3$), 123.8 ($C_6H_3$), 125.2 ($C_6H_3$), 142.6 ($C_6H_3$), 142.6 ($C_6H_3$), 145.1 ($C_6H_3$), 149.3 N($C$HCH)$_2$CN(CH$_3$)$_2$), 154.2 N(CHCH)$_2$$C$N(CH$_3$)$_2$), 167.9 ($C$N), 224.0 ($C$O).

ATR-IR: ν 2957, 2924, 2864, 1946, 1820, 1758, 1615, 1536, 1437, 1385, 1313, 1226, 1172, 1071, 1007, 934, 796, 757, 620, 525 cm^{-1}.

III. Spectroscopic Data of Complexes 1-7.

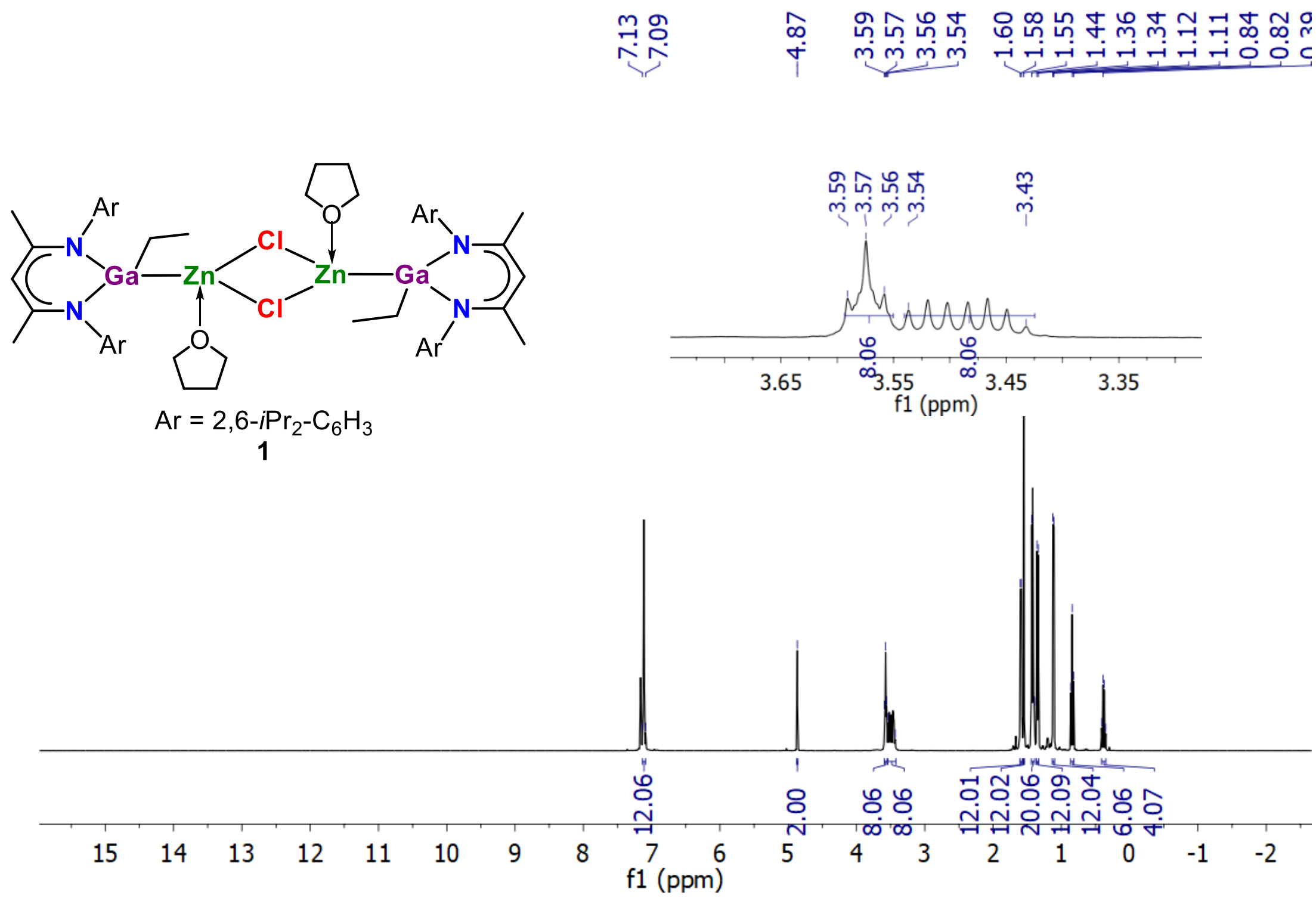

Figure S1. ^{1}H NMR (400 MHz, C_6D_6, 298 K) spectrum of compound **1**.

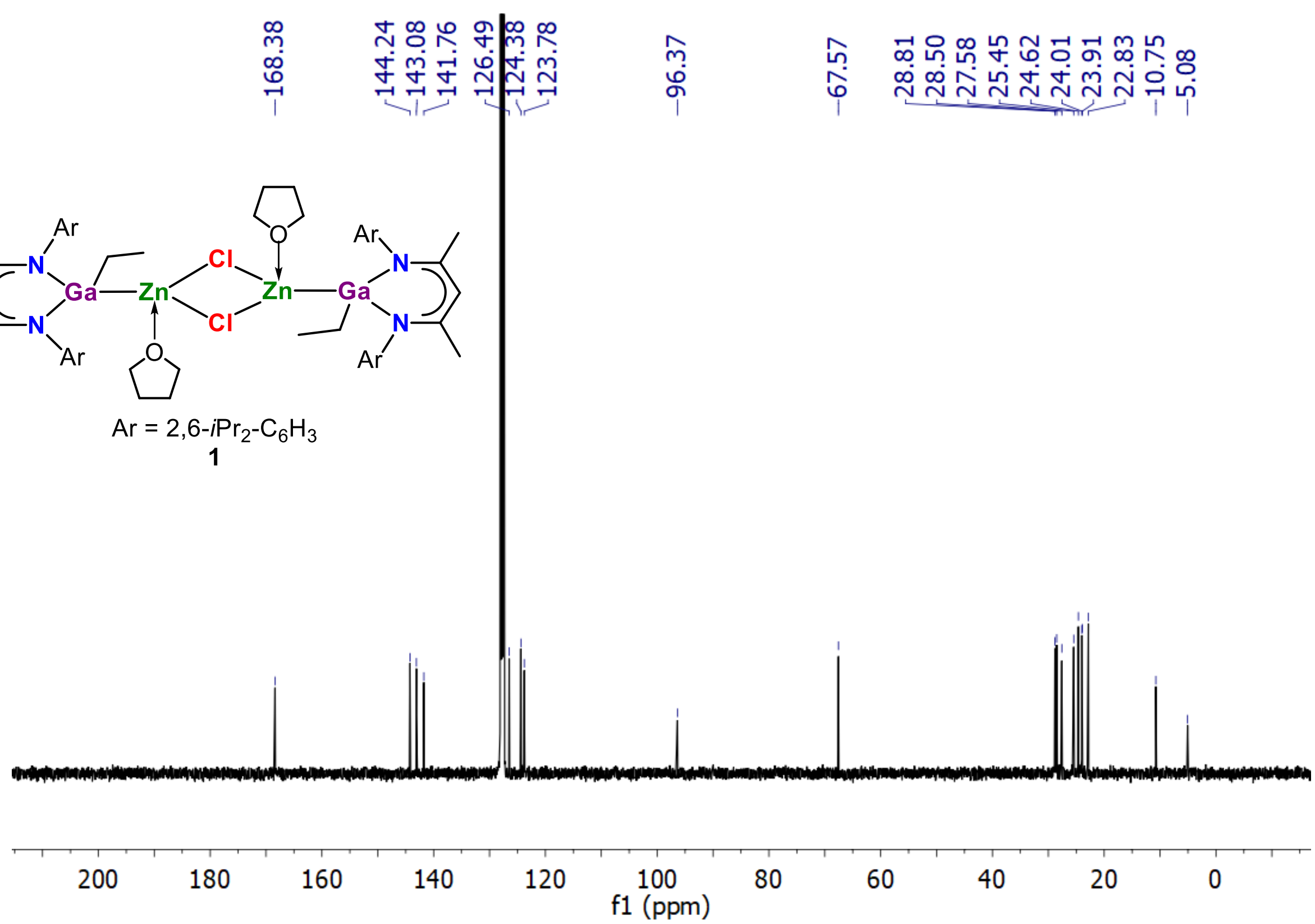

Figure S2. $^{13}C\{^{1}H\}$ NMR (100 MHz, C_6D_6, 298 K) spectrum of compound **1**.

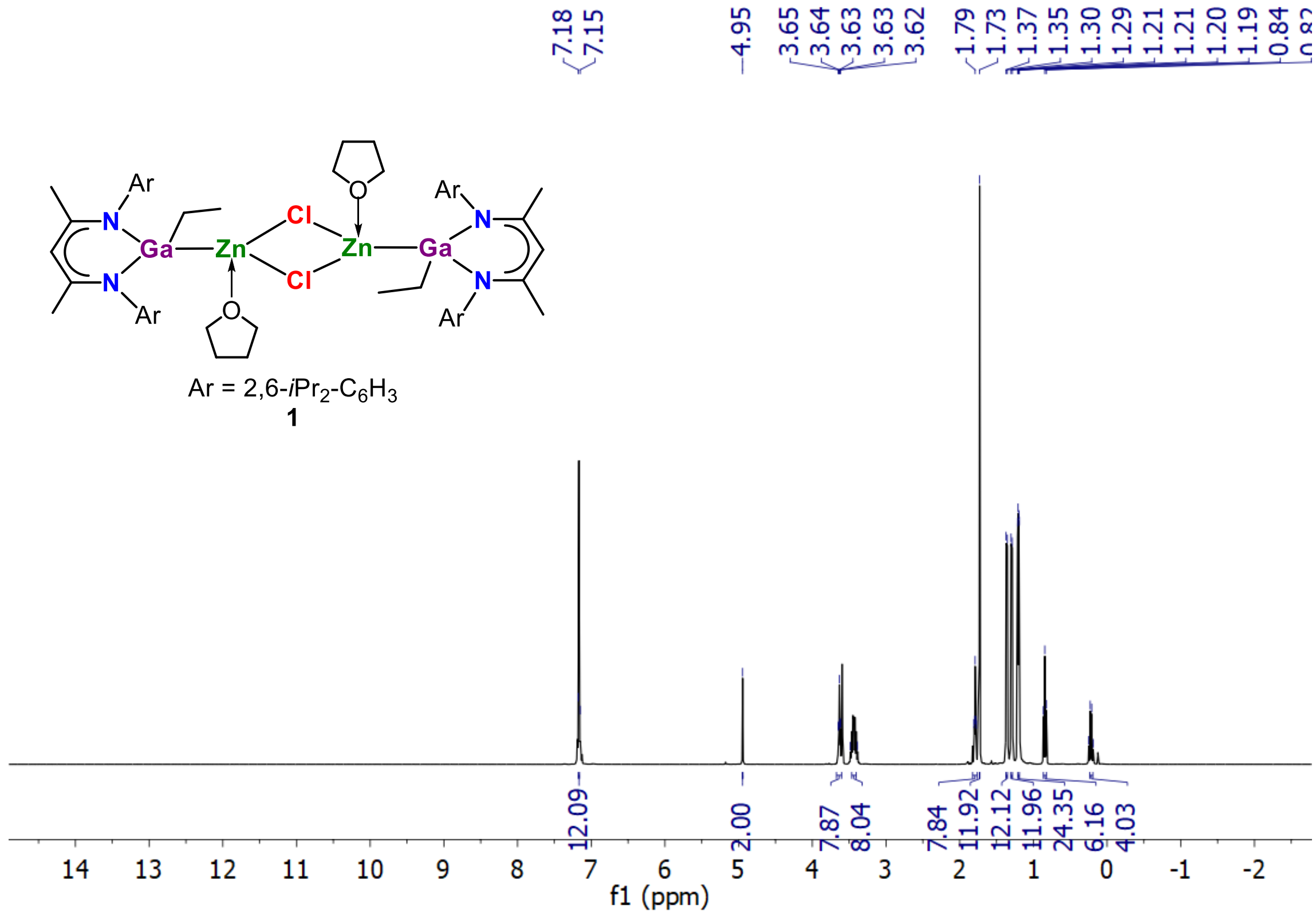

Figure S3. ^{1}H NMR (400 MHz, THF-d_8, 298 K) spectrum of compound **1**.

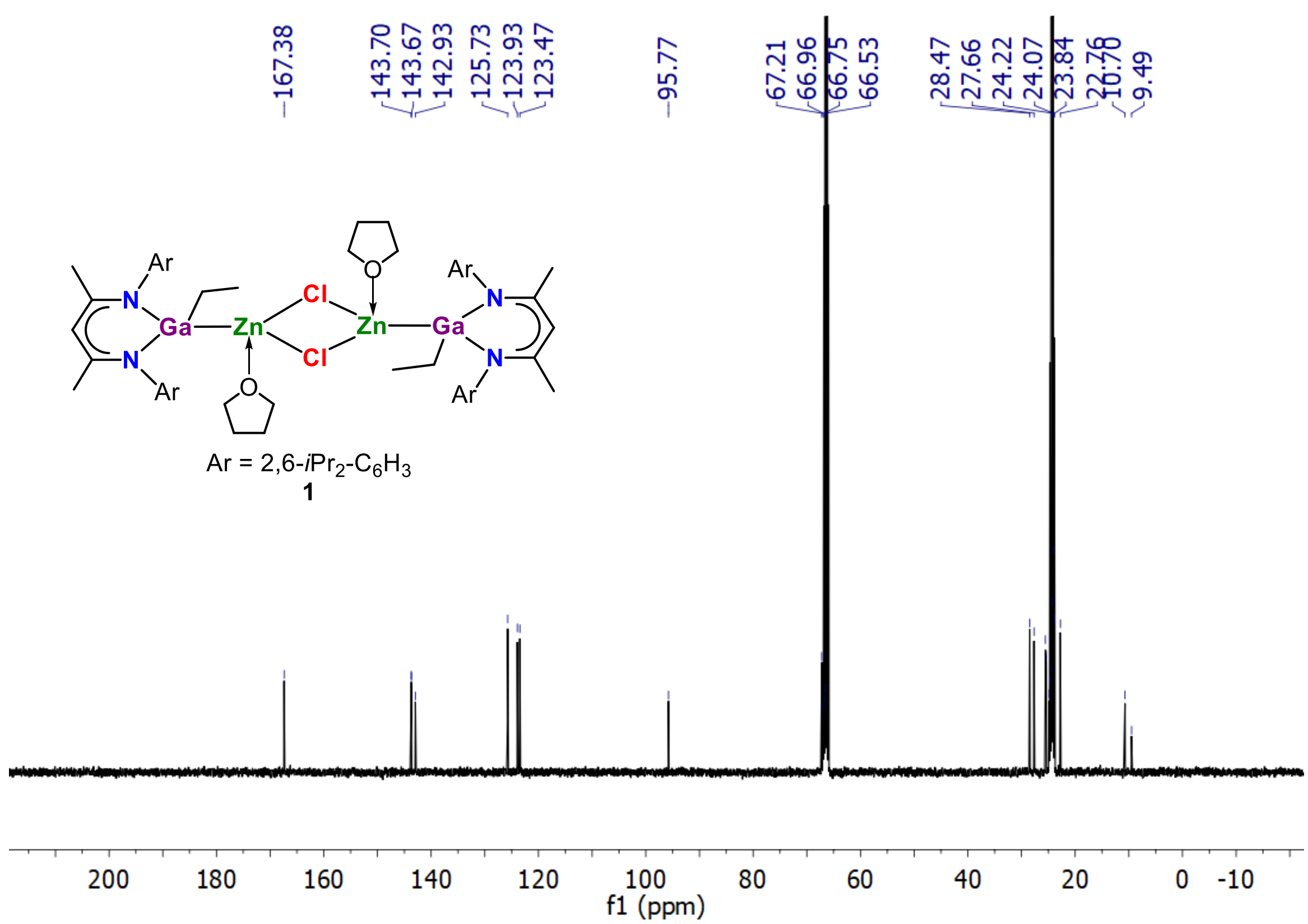

Figure S4. ^{13}C{^{1}H} NMR (100 MHz, THF-d_8, 298 K) spectrum of compound **1**.

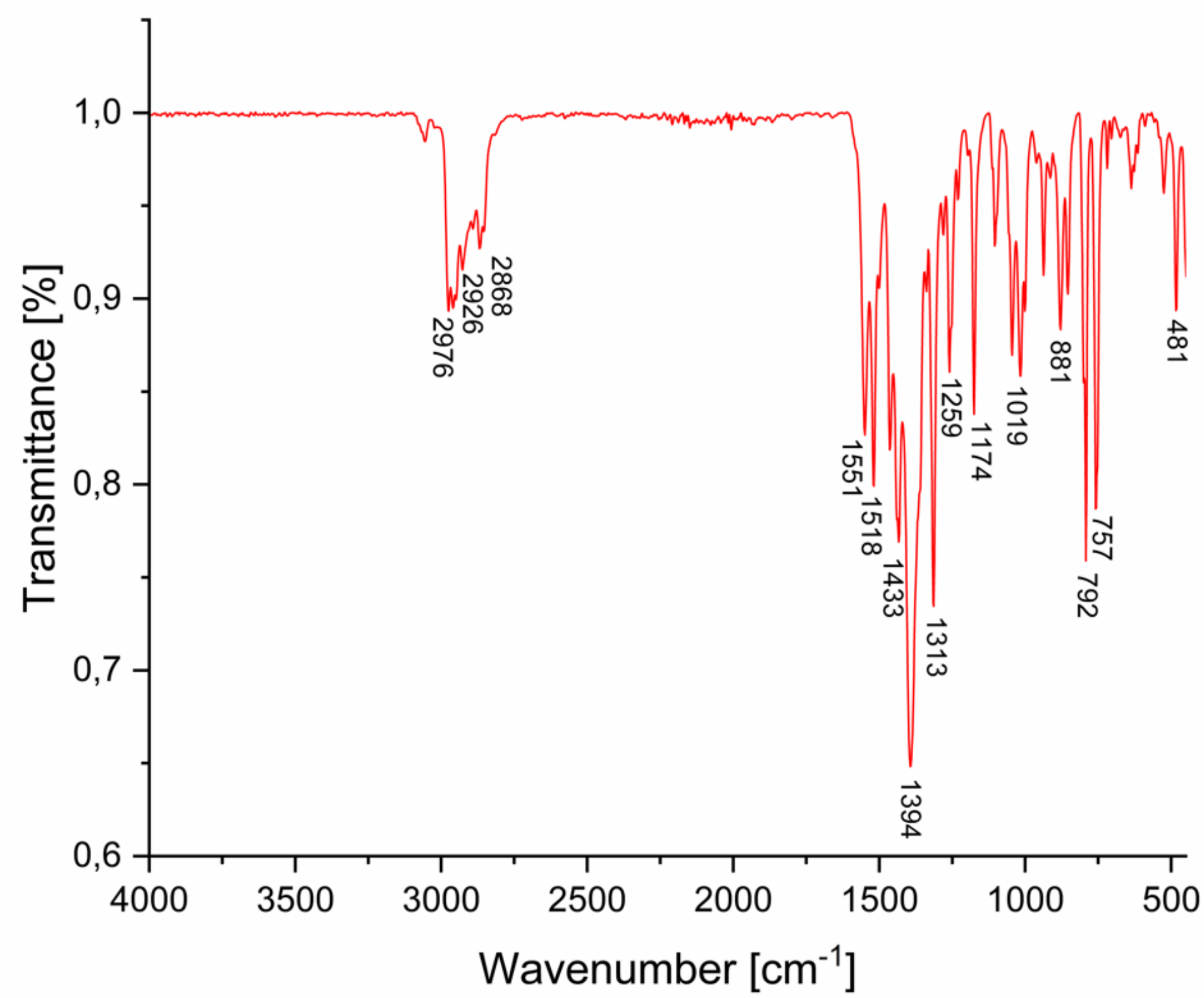

Figure S5. ATR-IR spectrum of compound **1**.

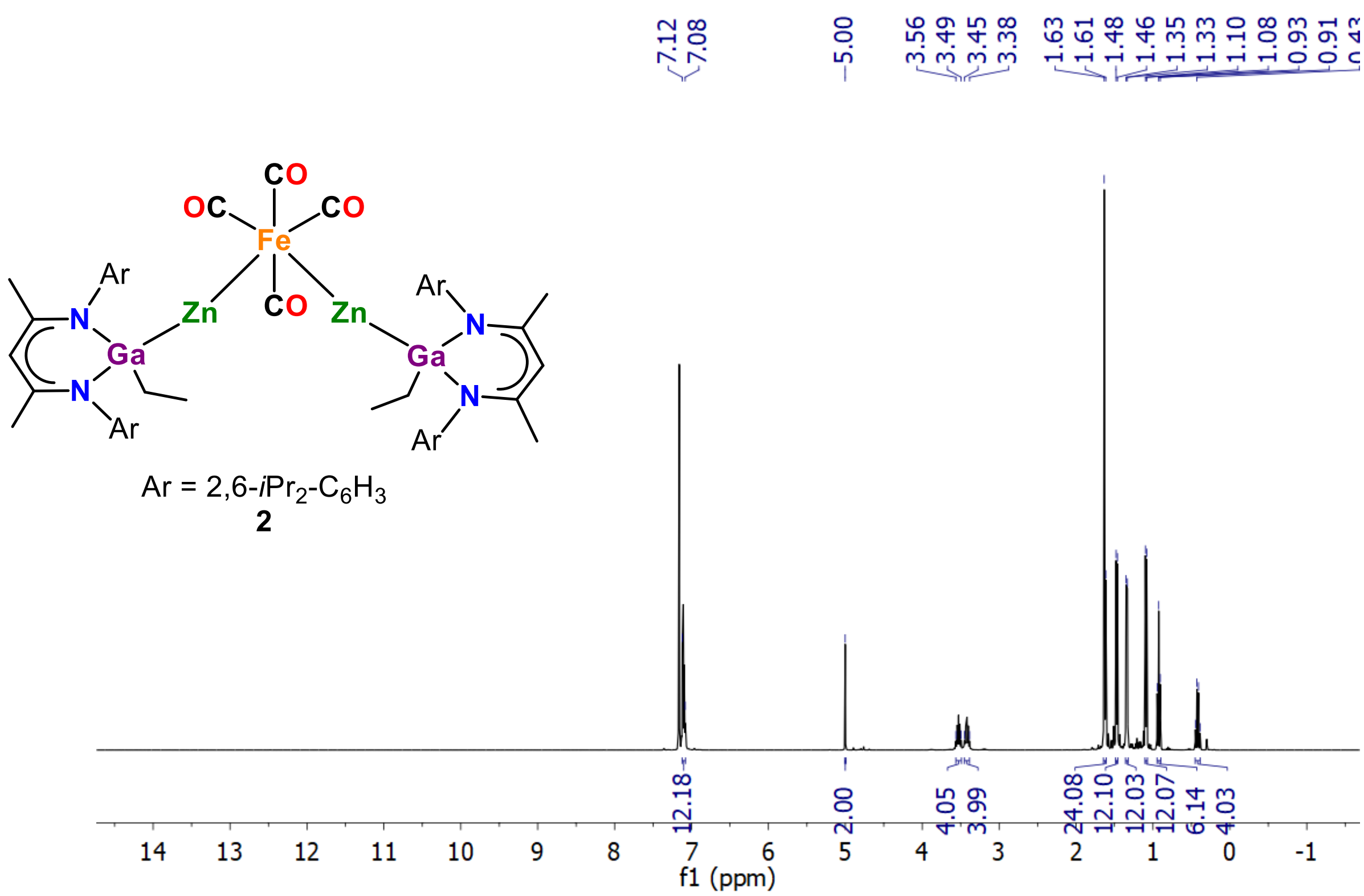

Figure S6. ^{1}H NMR (400 MHz, C_6D_6, 298 K) spectrum of compound **2**.

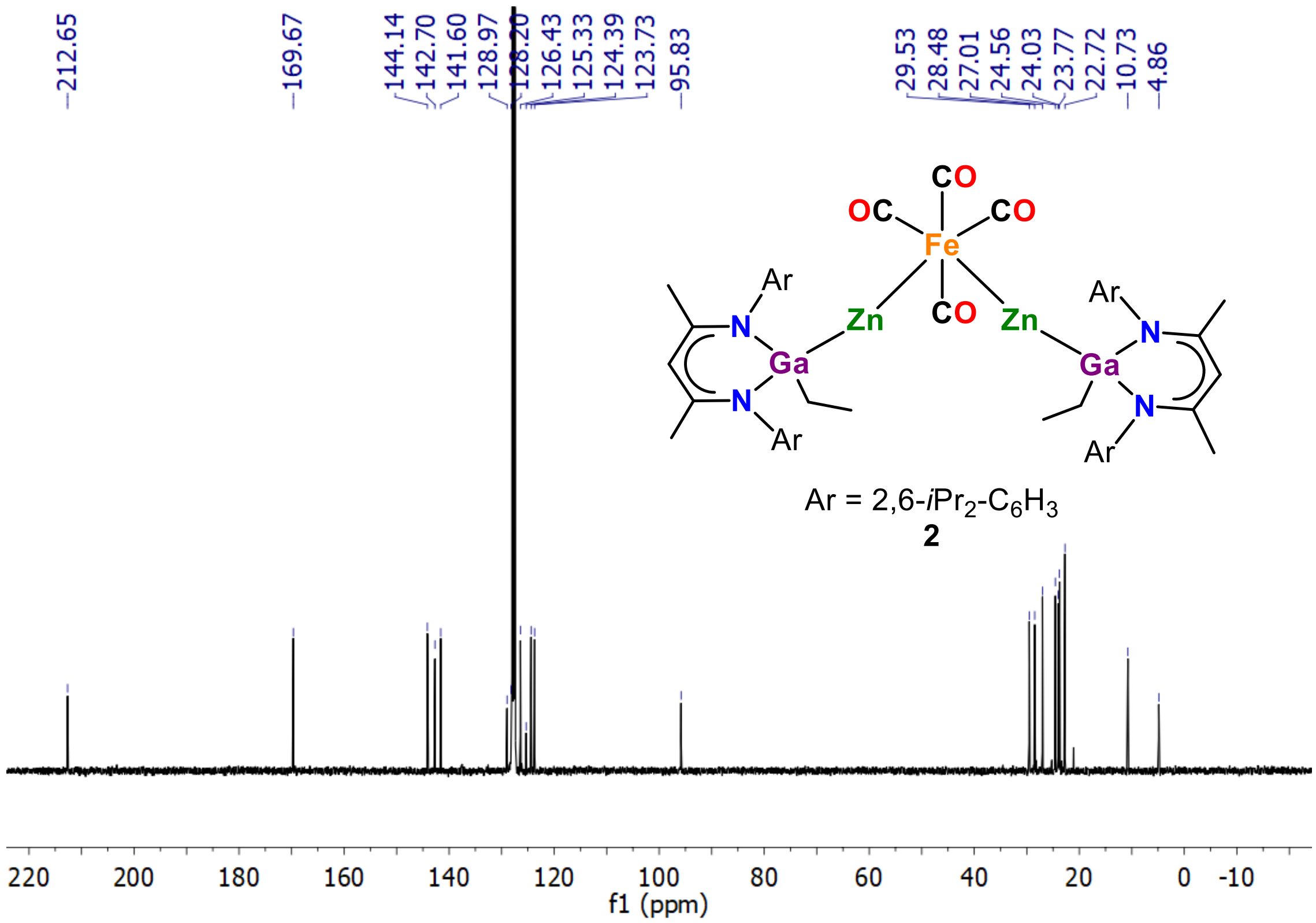

Figure S7. $^{13}C\{^1H\}$ NMR (100 MHz, C_6D_6, 298 K) spectrum of compound **2**.

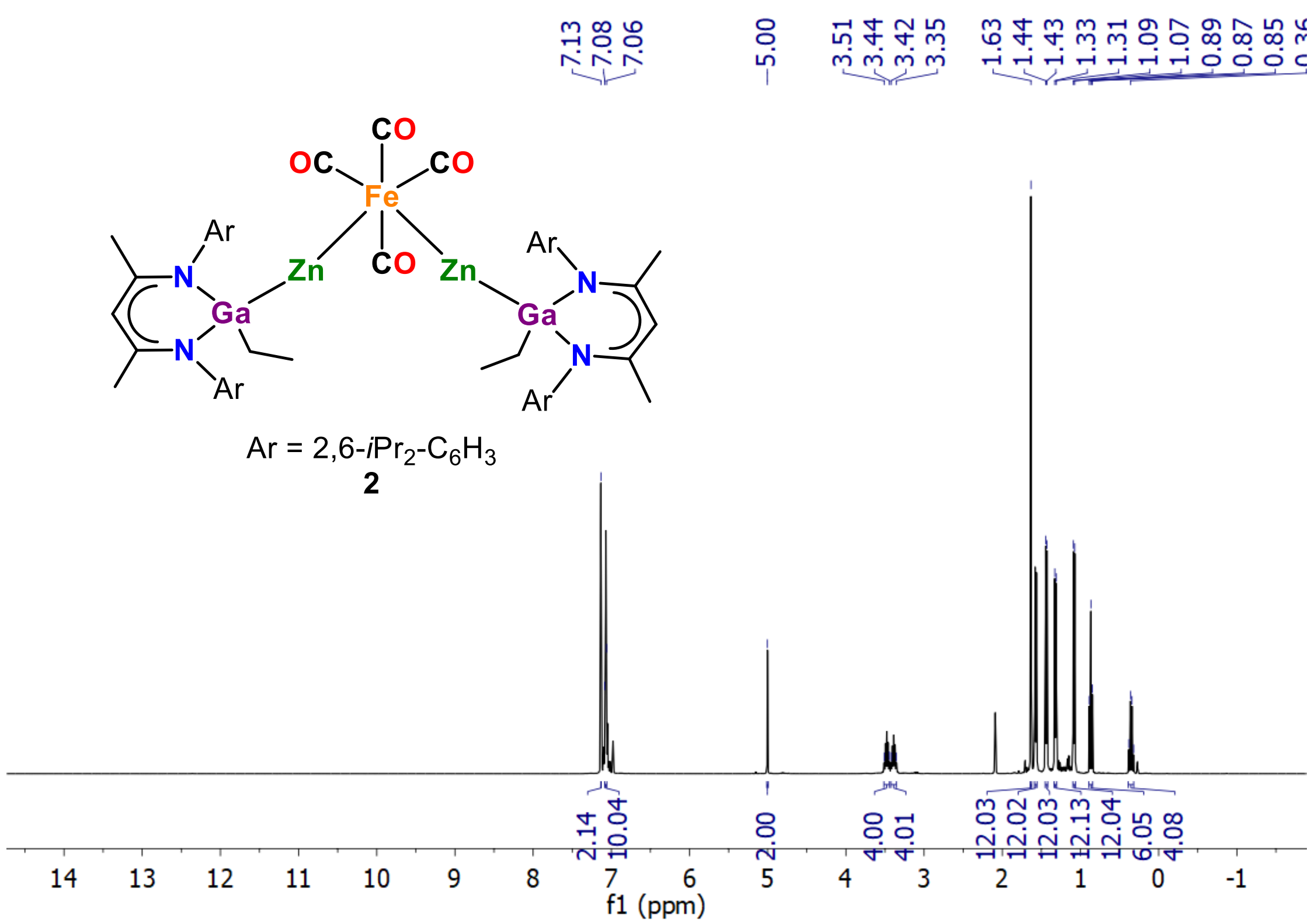

Figure S8. ^1H NMR (400 MHz, toluene-d_8, 298 K) spectrum of compound **2**.

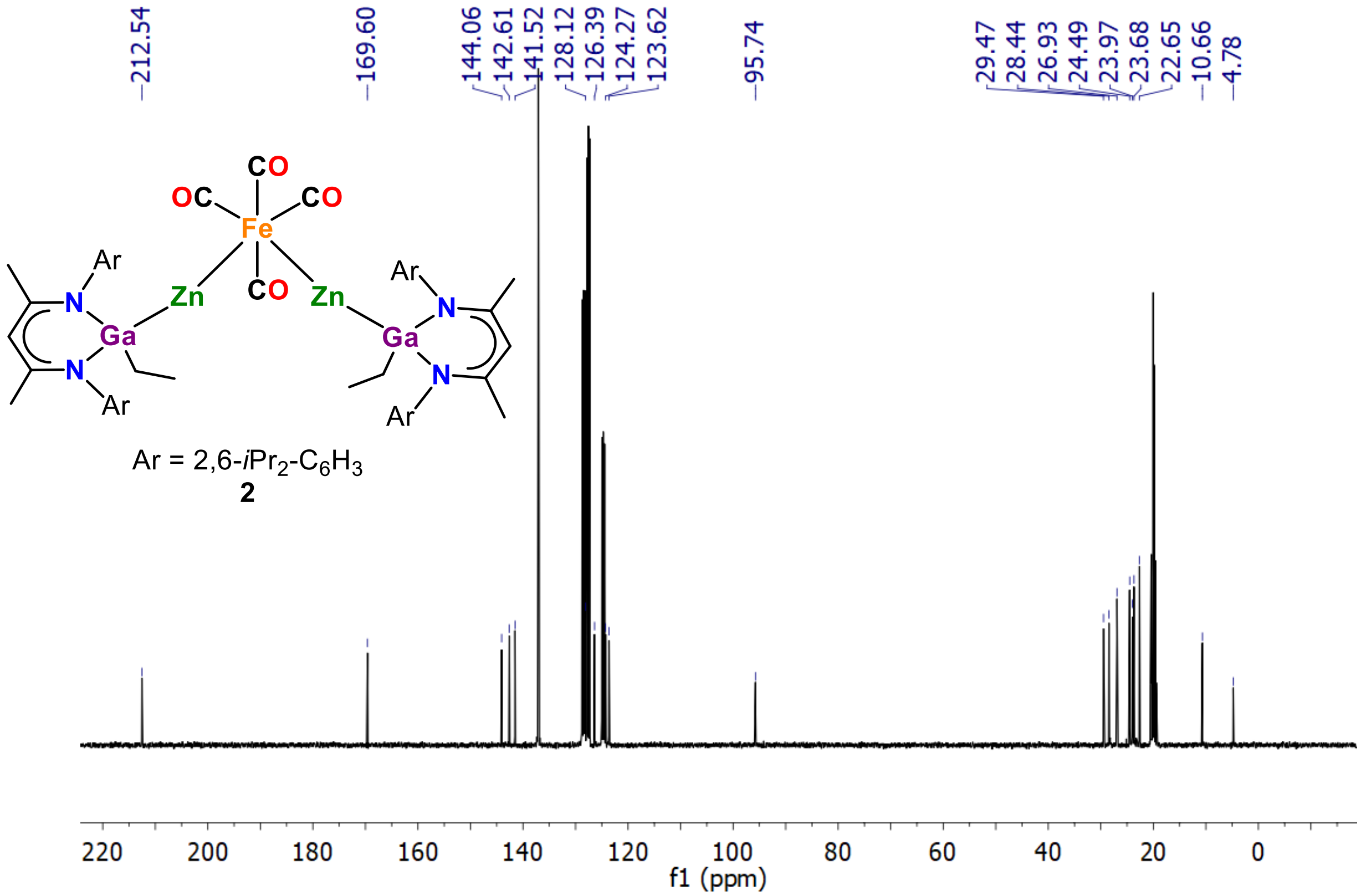

Figure S9. $^{13}C\{^{1}H\}$ NMR (100 MHz, toluene-d$_8$, 298 K) spectrum of compound **2**.

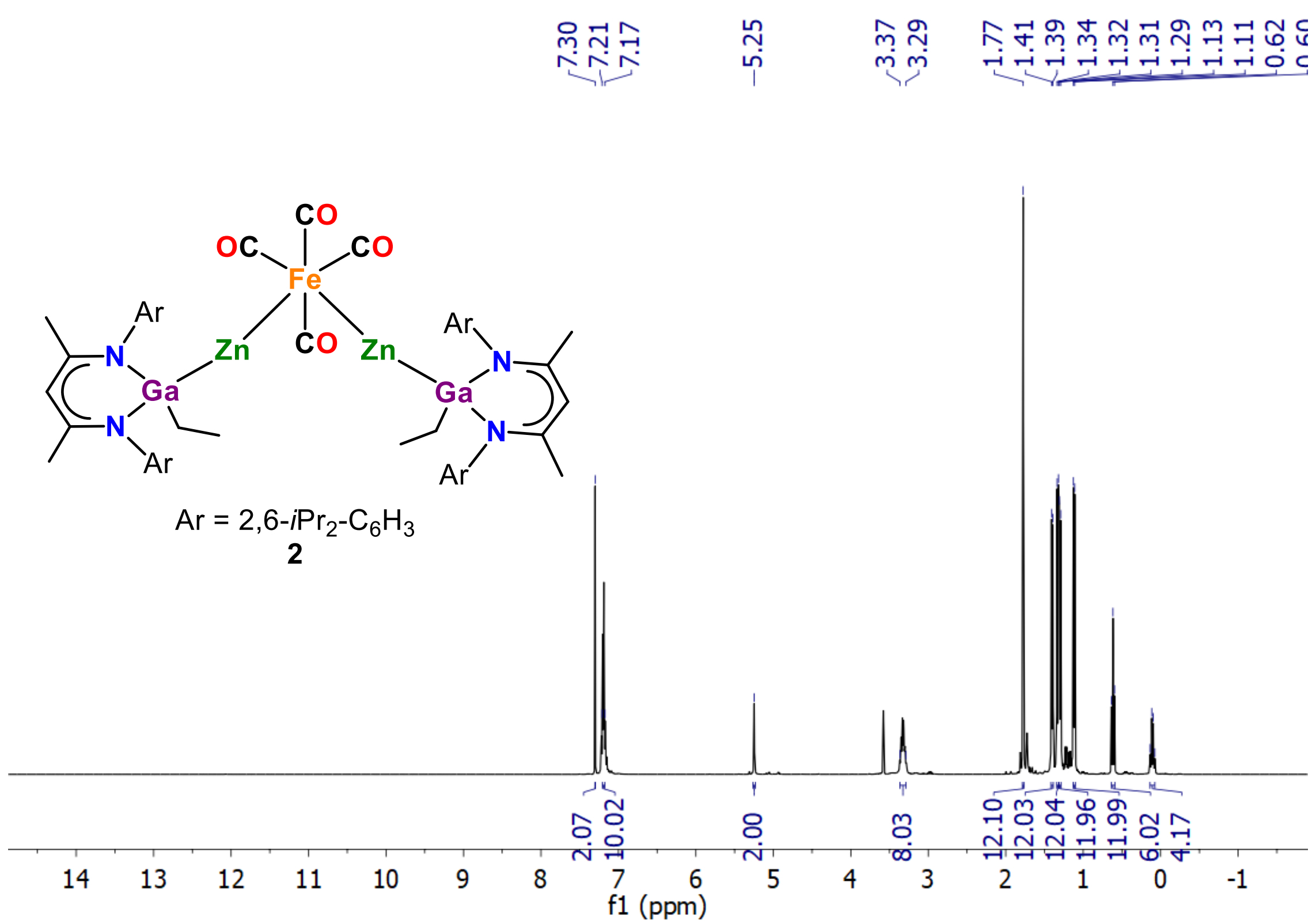

Figure S10. ^{1}H NMR (400 MHz, THF-d$_8$, 298 K) spectrum of compound **2**.

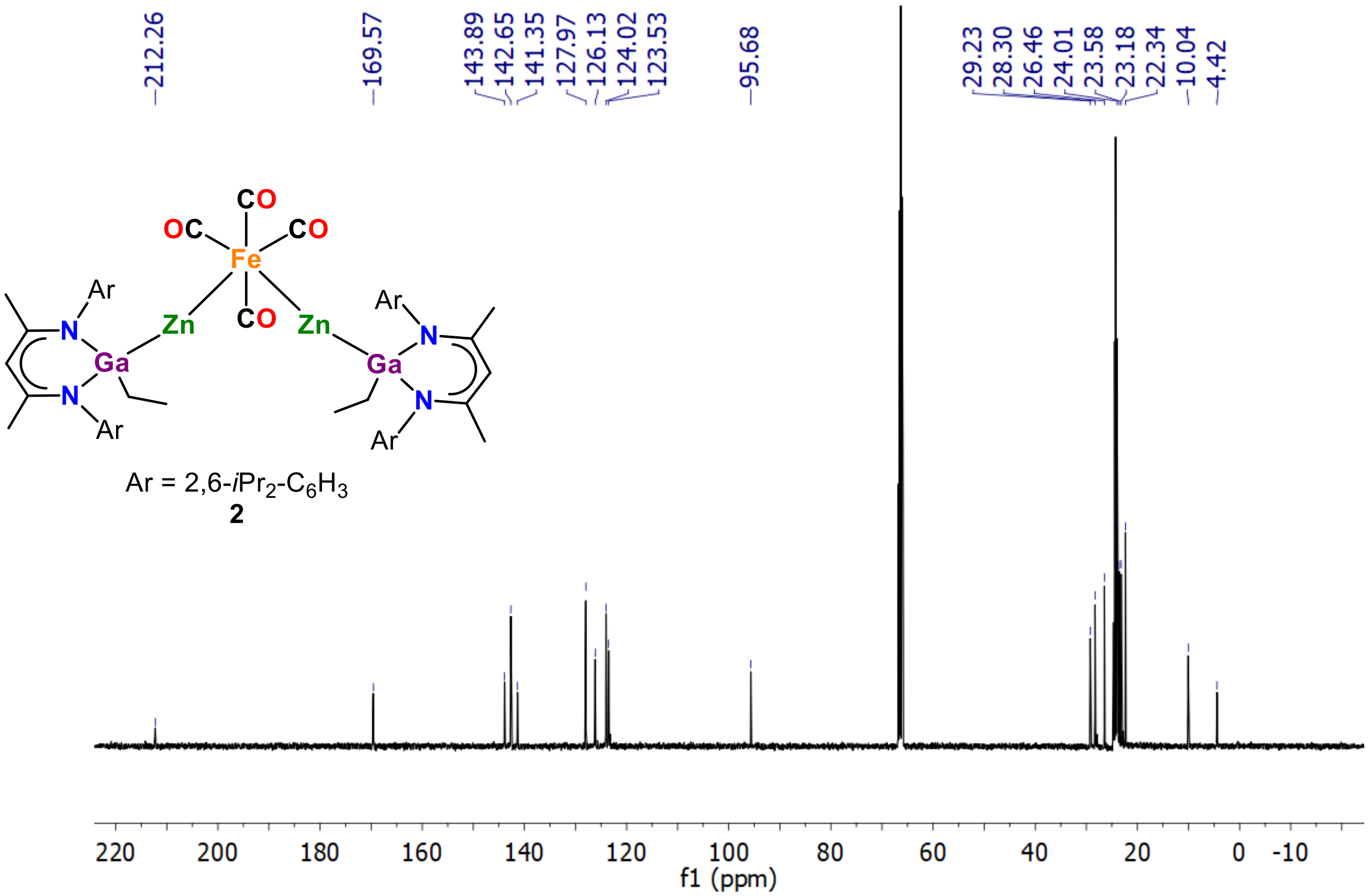

Figure S11. $^{13}C\{^{1}H\}$ NMR (100 MHz, THF-d$_8$, 298 K) spectrum of compound **2**.

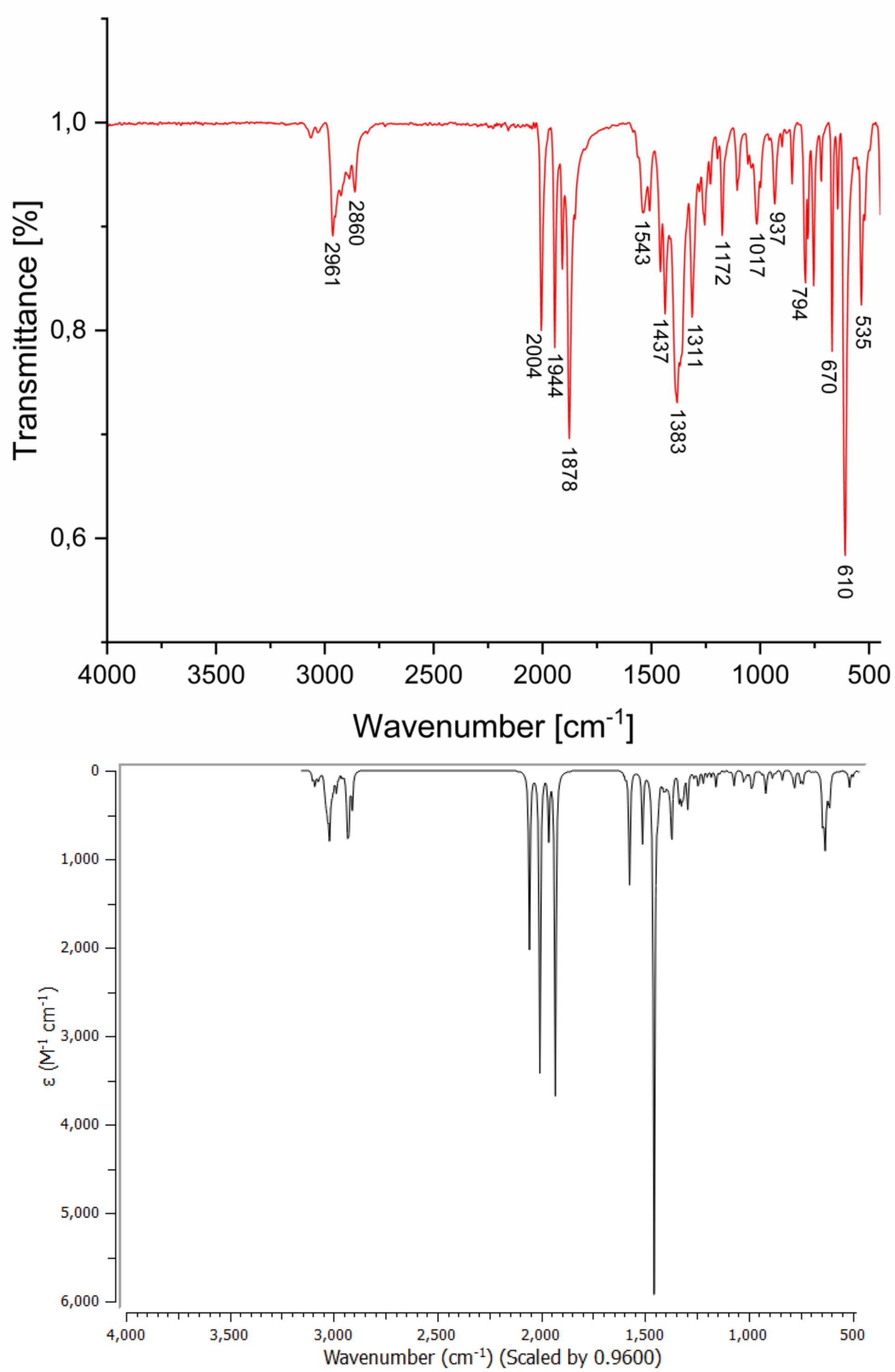

Figure S12. Experimentally determined ATR-IR spectrum (above) and simulated IR spectrum (below, PBE0-D3BJ/def2-SVP) of compound **2**. The simulated stretching vibrations for the C=O bands amount to 1938, 1970, 2013, and 2063 cm^{-1}.

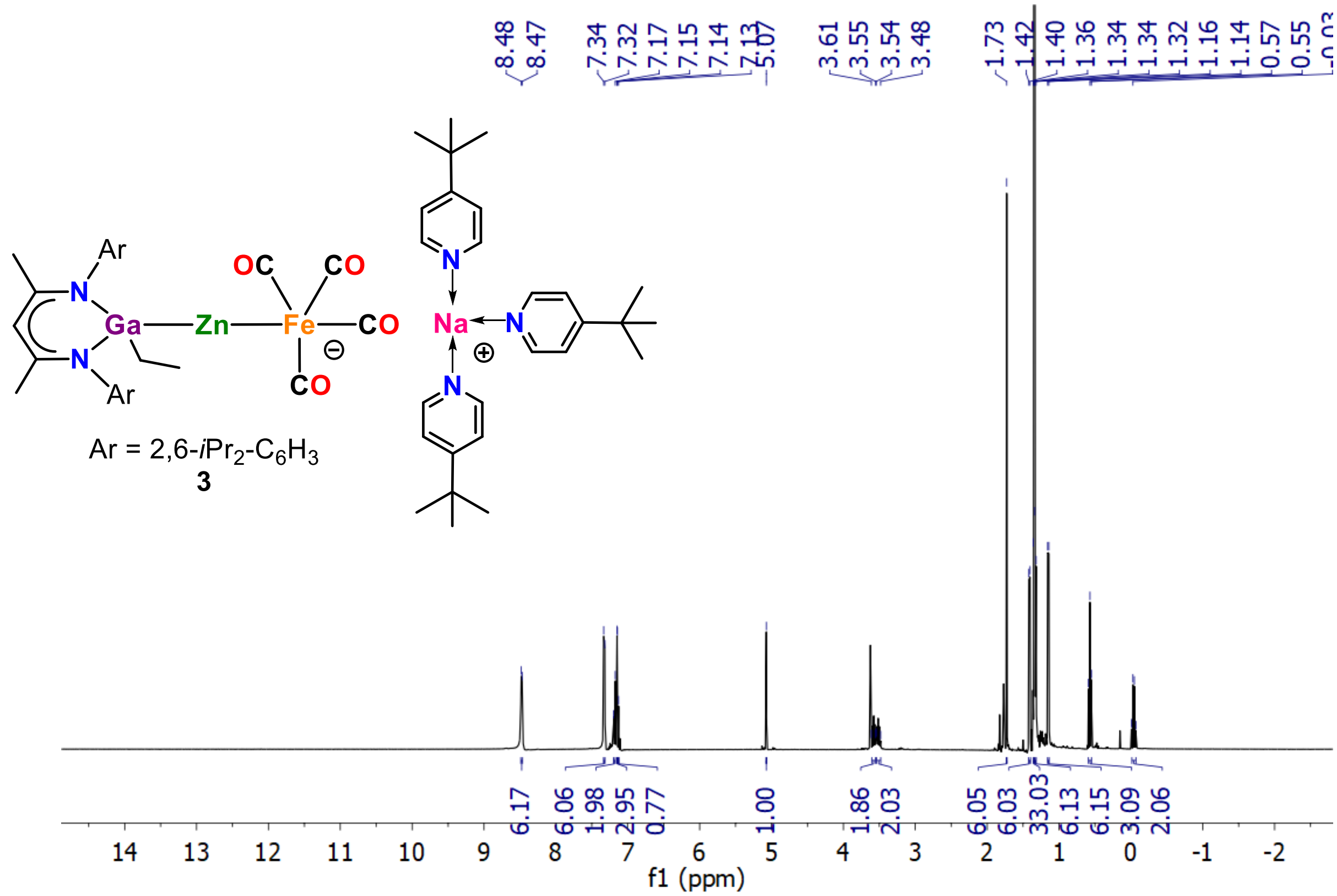

Figure S13. ^{1}H NMR (400 MHz, THF-d$_8$, 298 K) spectrum of compound **3**.

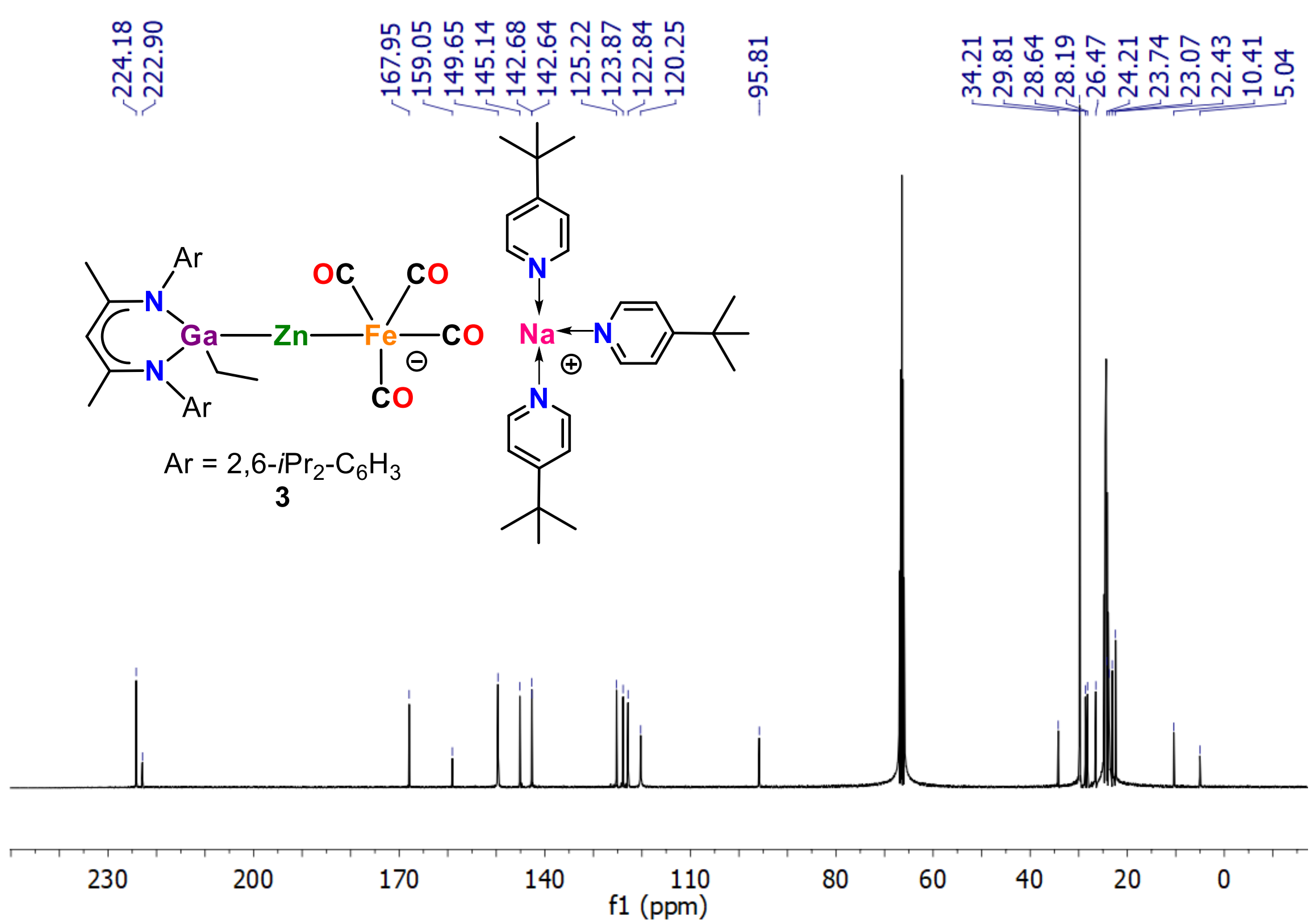

Figure S14. ^{13}C{^{1}H} NMR (100 MHz, THF-d$_8$, 298 K) spectrum of compound **3**.

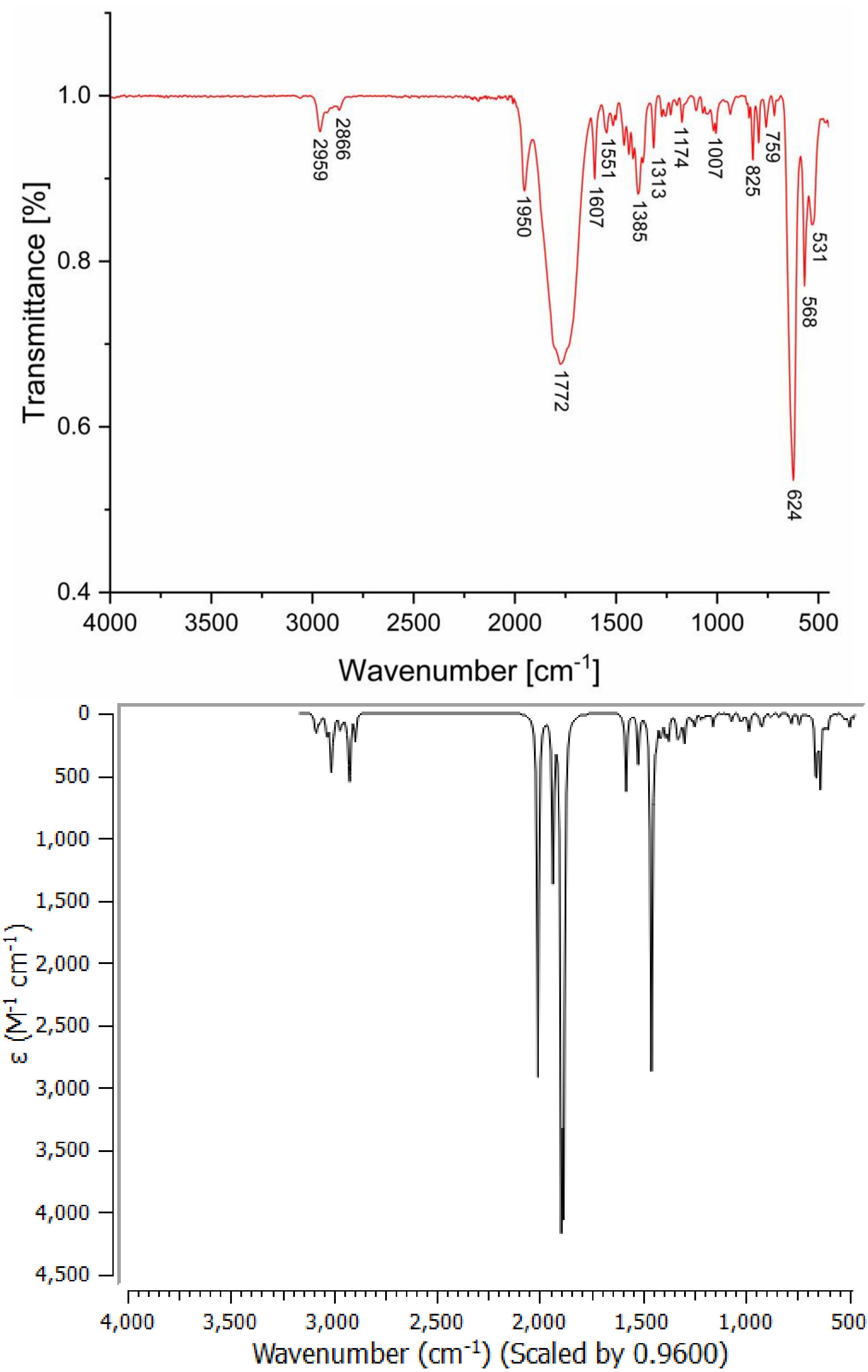

Figure S15. Experimentally determined ATR-IR spectrum of **3** (above) and simulated IR spectrum (below, PBE0-D3BJ/def2-SVP) of the anionic part of **3**. The simulated stretching vibrations for the C=O bands amount to 1889, 1900, 1940, and 2014 cm^{-1}.

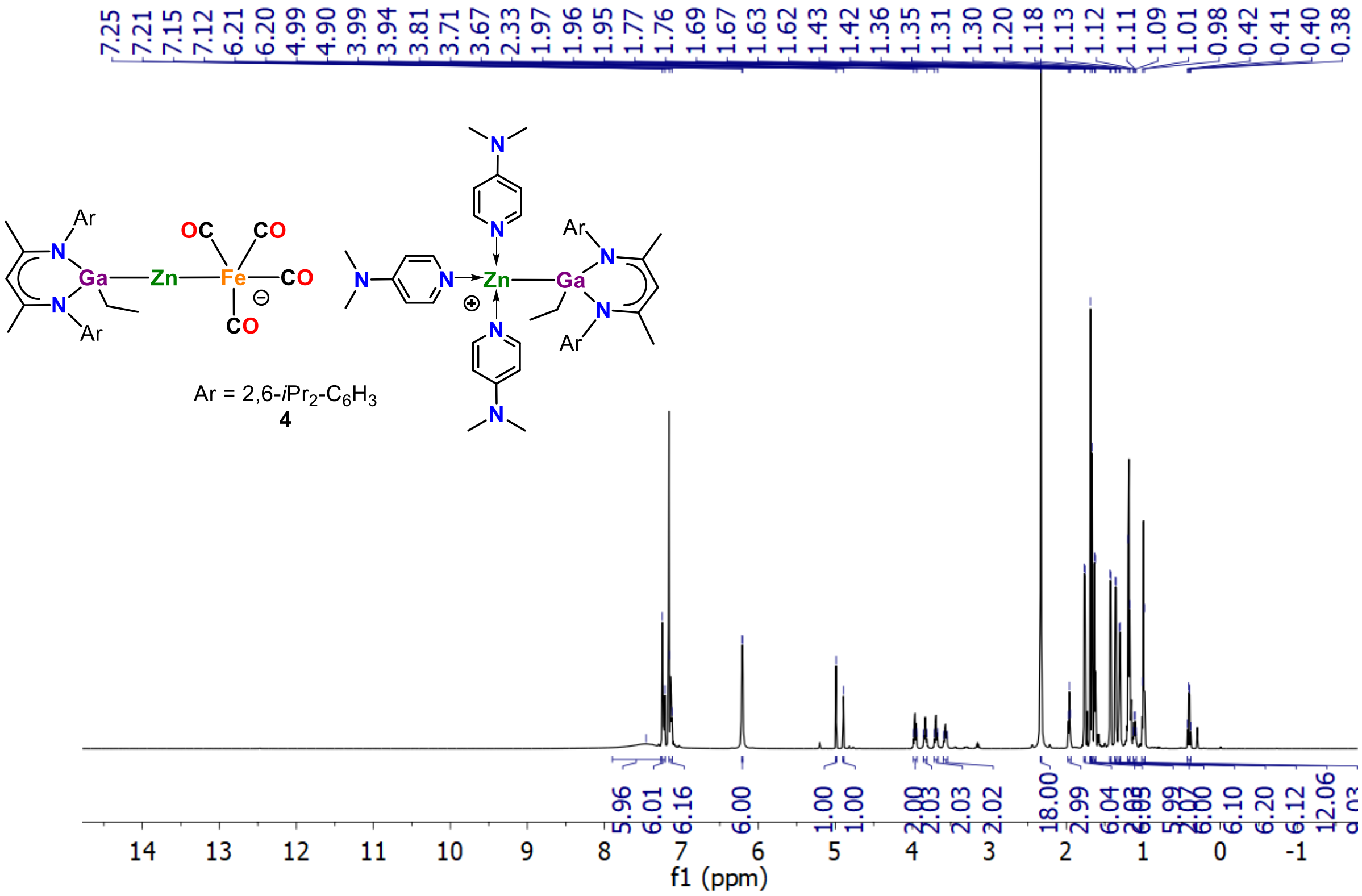

Figure S16. ^{1}H NMR (400 MHz, C_6D_6, 298 K) spectrum of compound **4**.

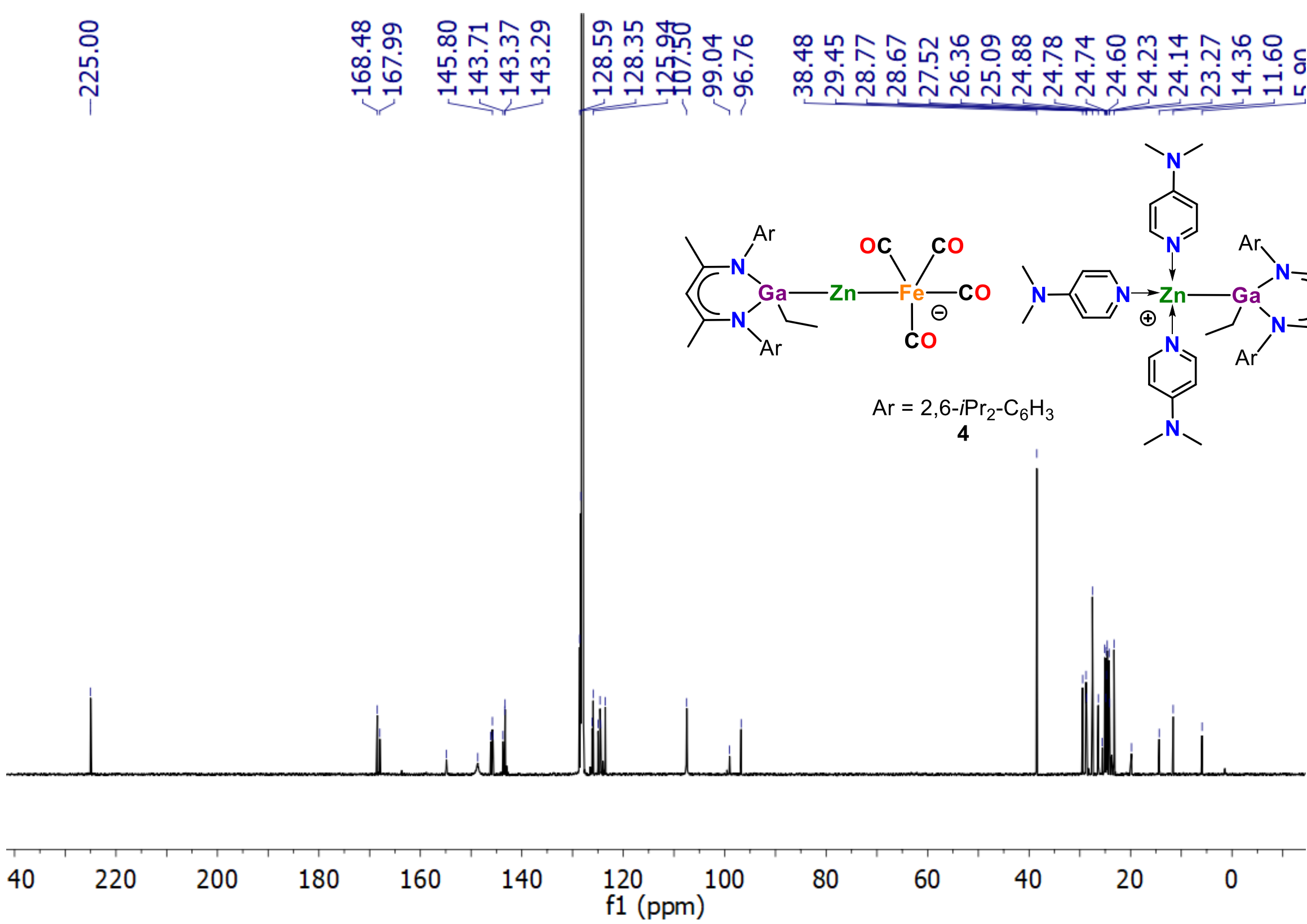

Figure S17. $^{13}C\{^{1}H\}$ NMR (100 MHz, C_6D_6, 298 K) spectrum of compound **4**.

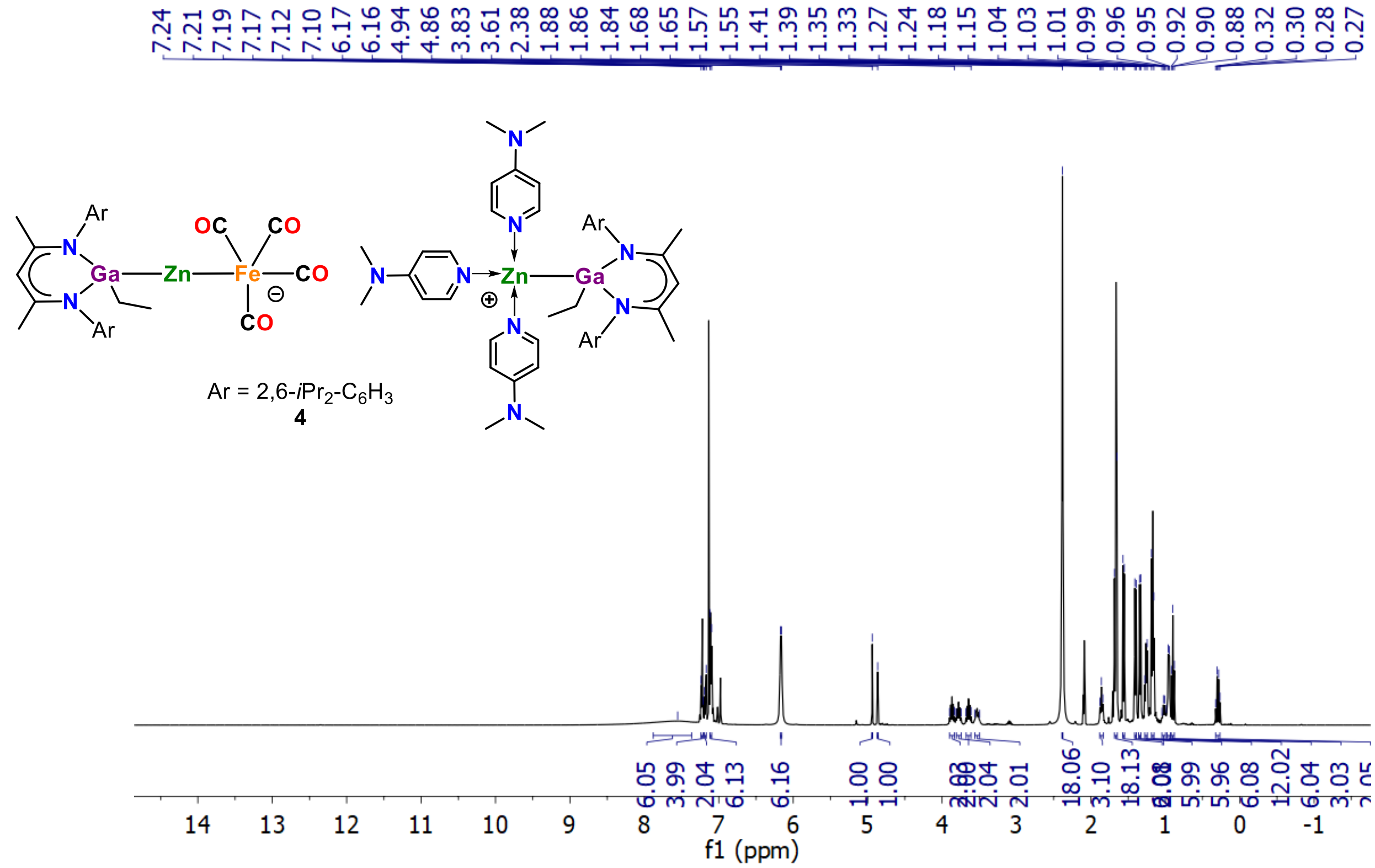

Figure S18. ^{1}H NMR (400 MHz, toluene-d$_8$, 298 K) spectrum of compound **4**.

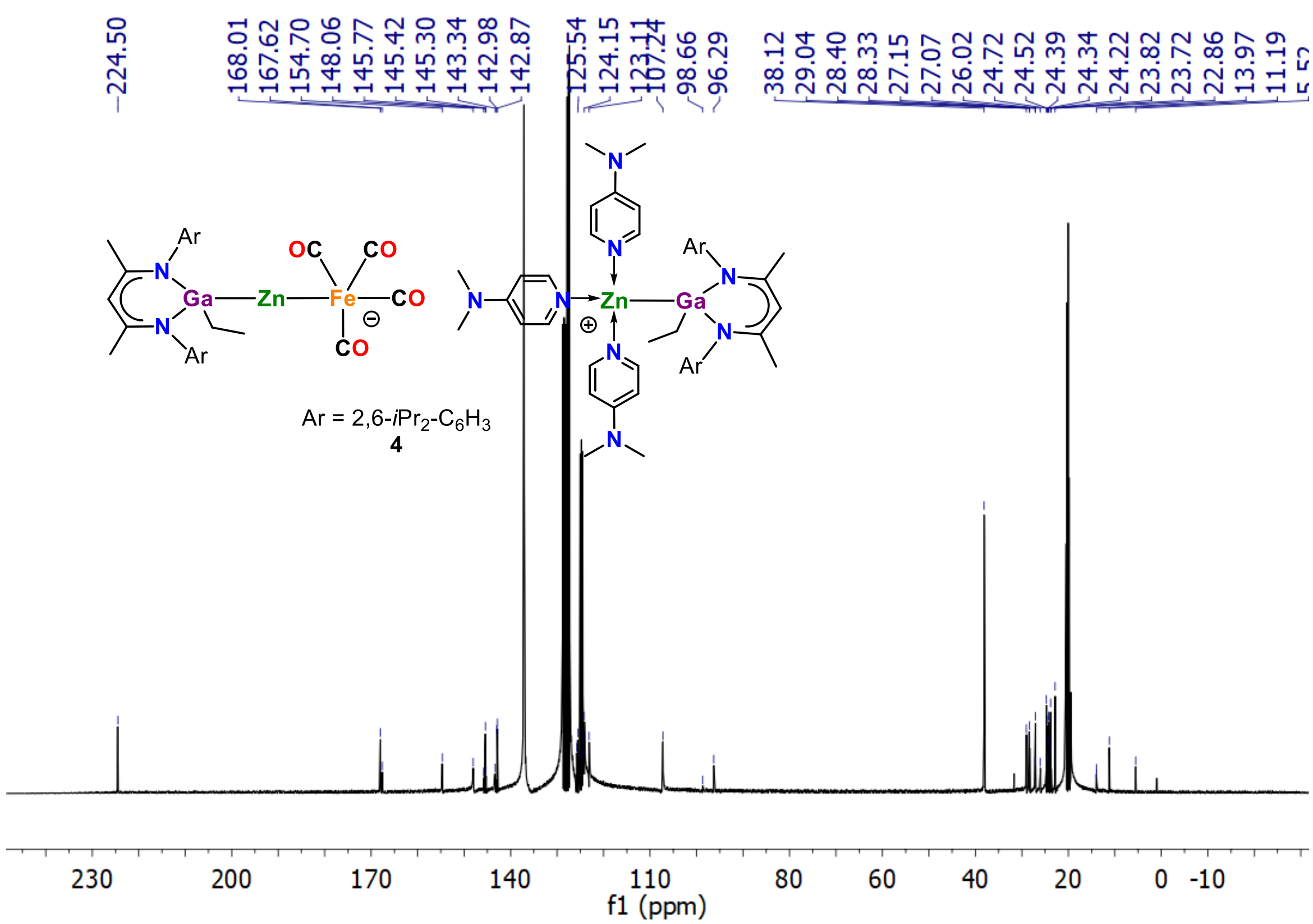

Figure S19. $^{13}C\{^{1}H\}$ NMR (100 MHz, toluene-d$_8$, 298 K) spectrum of compound **4**.

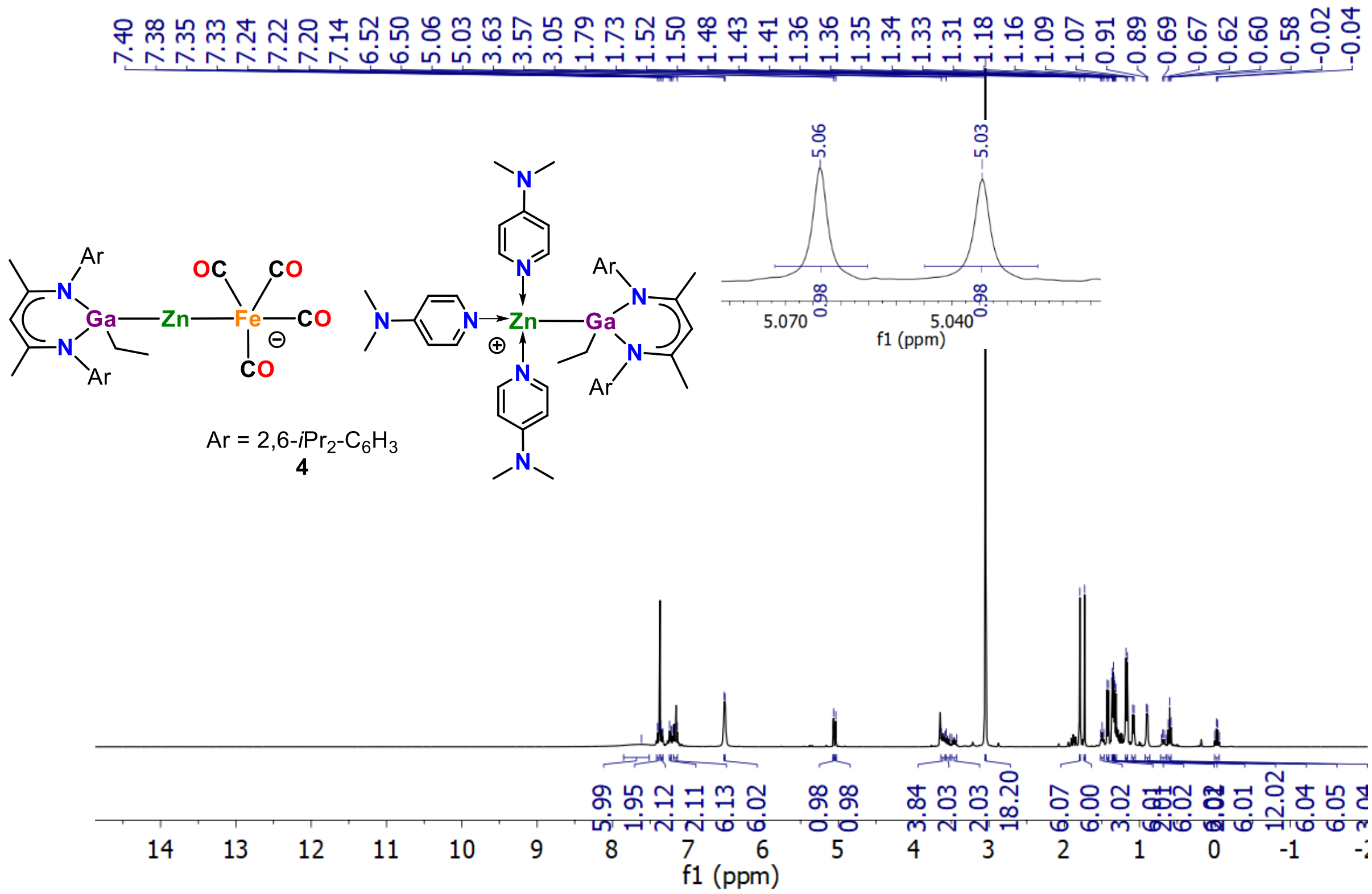

Figure S20. ^{1}H NMR (400 MHz, THF-d$_8$, 298 K) spectrum of compound **4**.

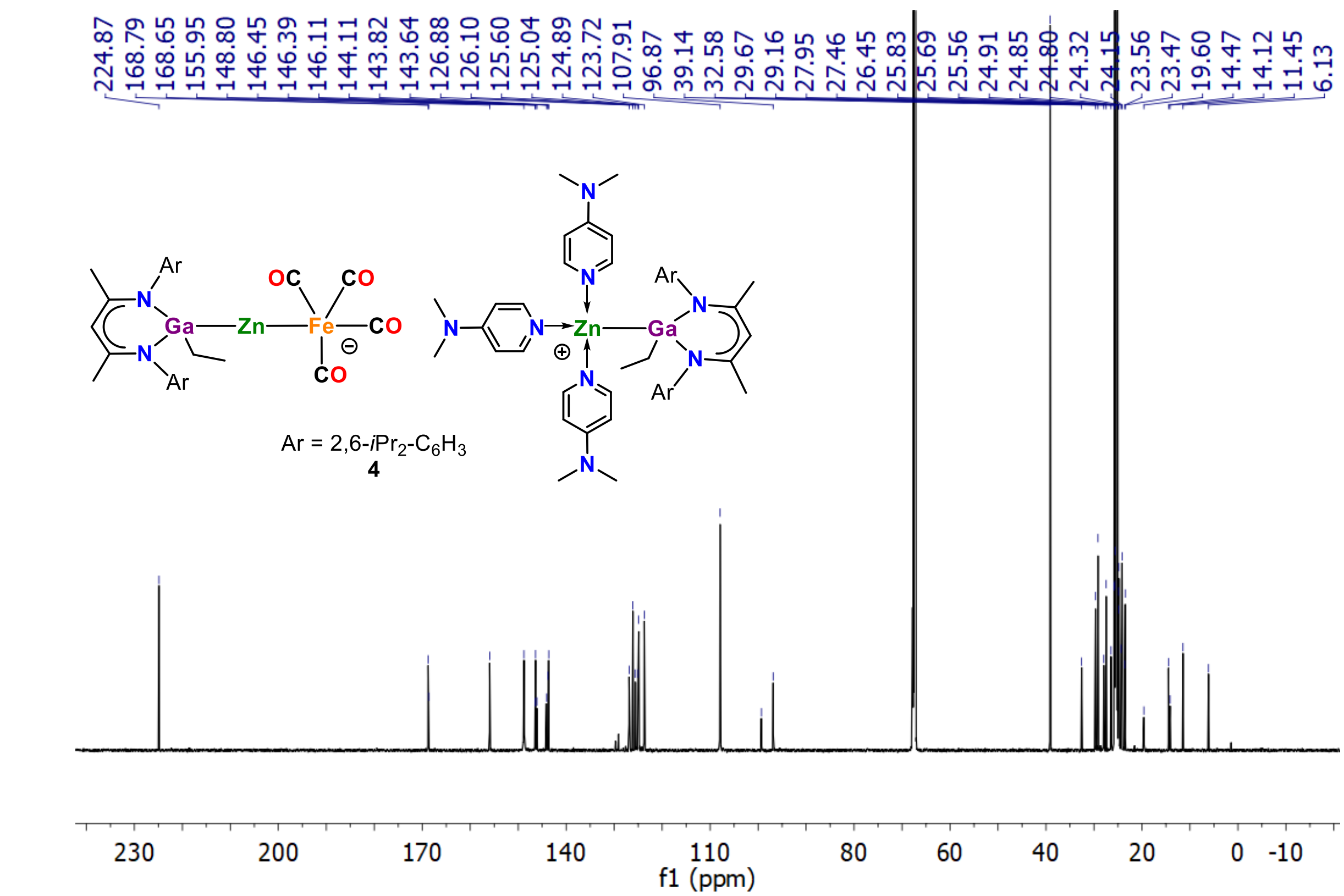

Figure S21. ^{13}C{^{1}H} NMR (100 MHz, THF-d$_8$, 298 K) spectrum of compound **4**.

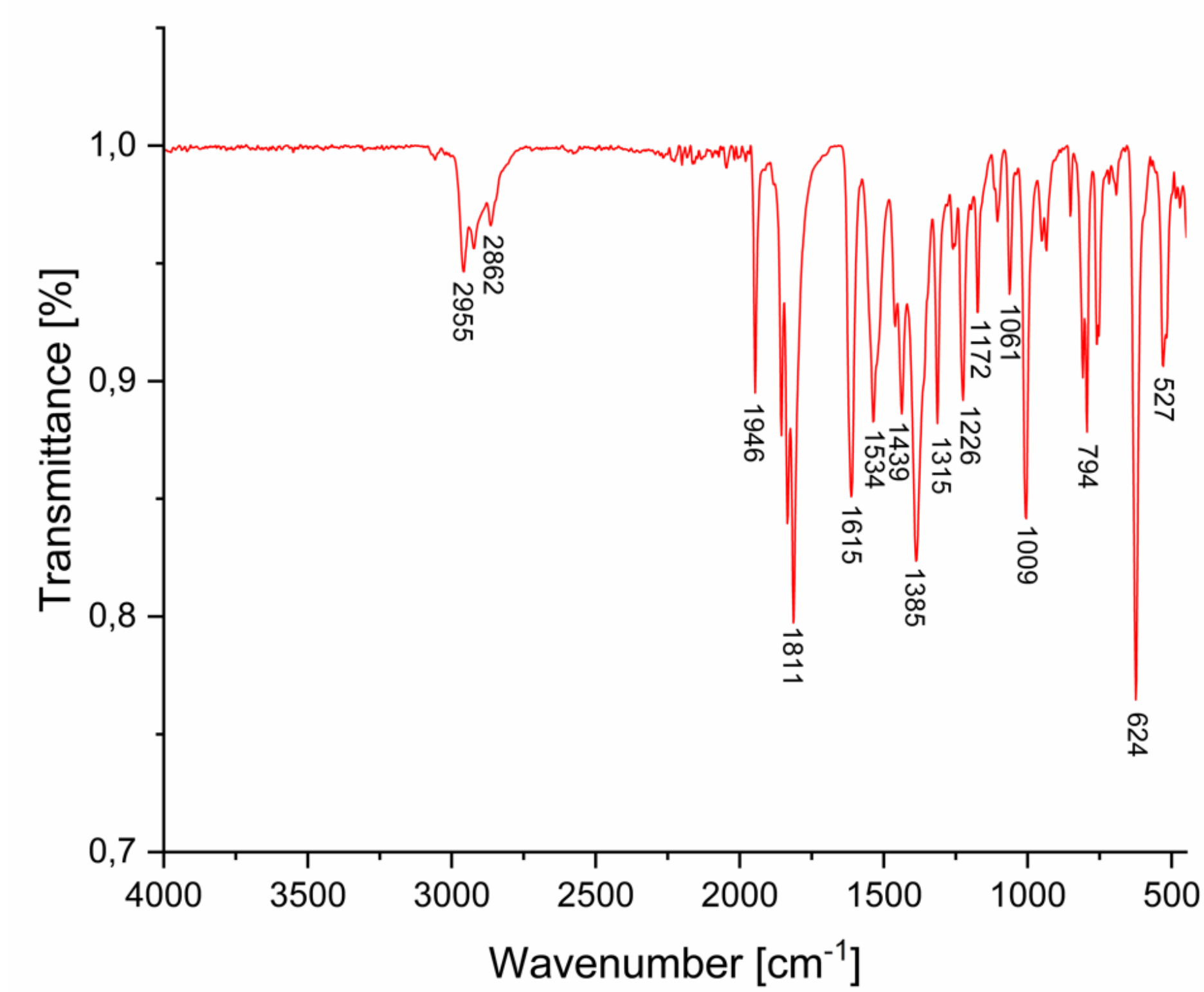

Figure S22. ATR-IR spectrum of compound **4**.

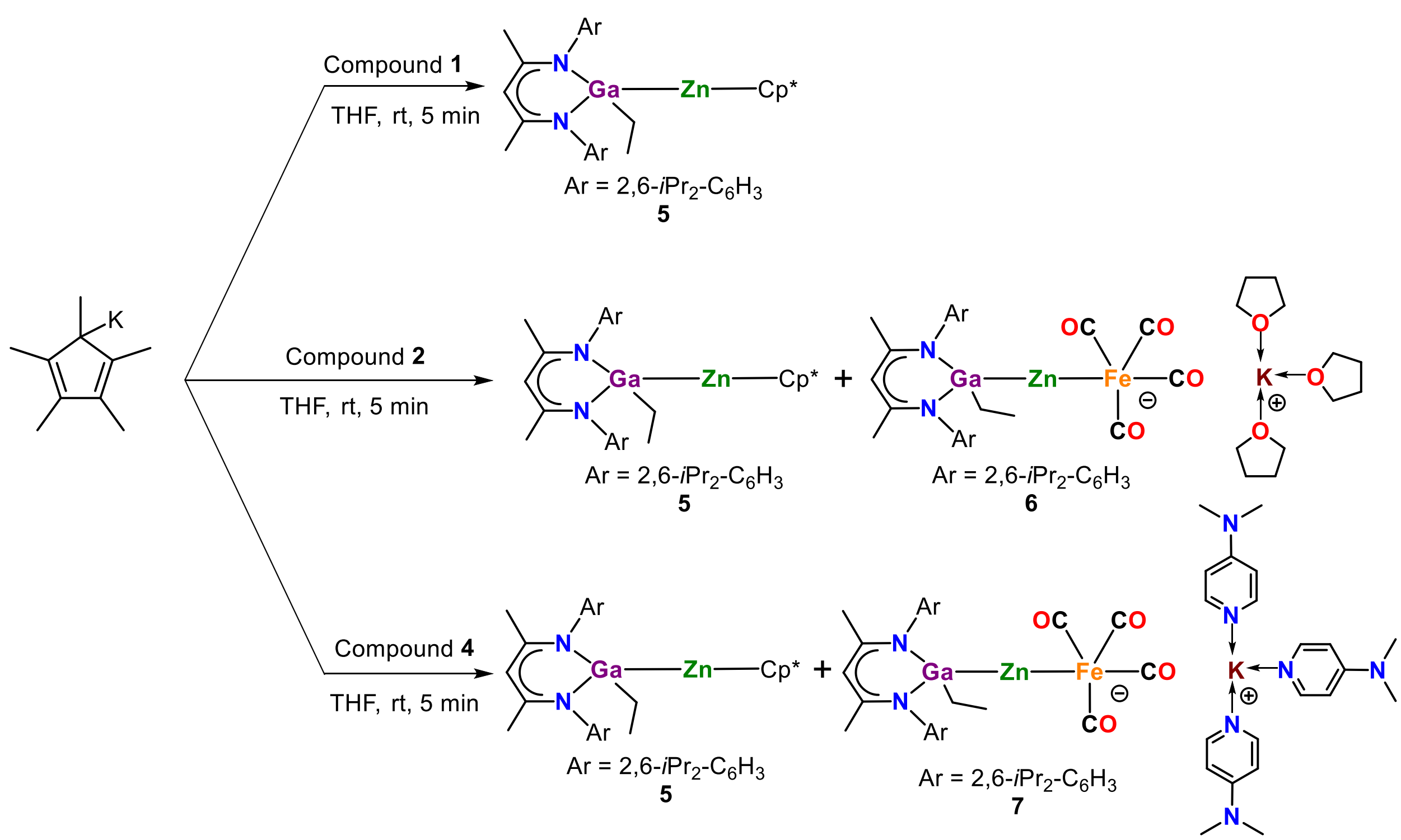

Figure S23: Synthesis of complexes **5-7**.

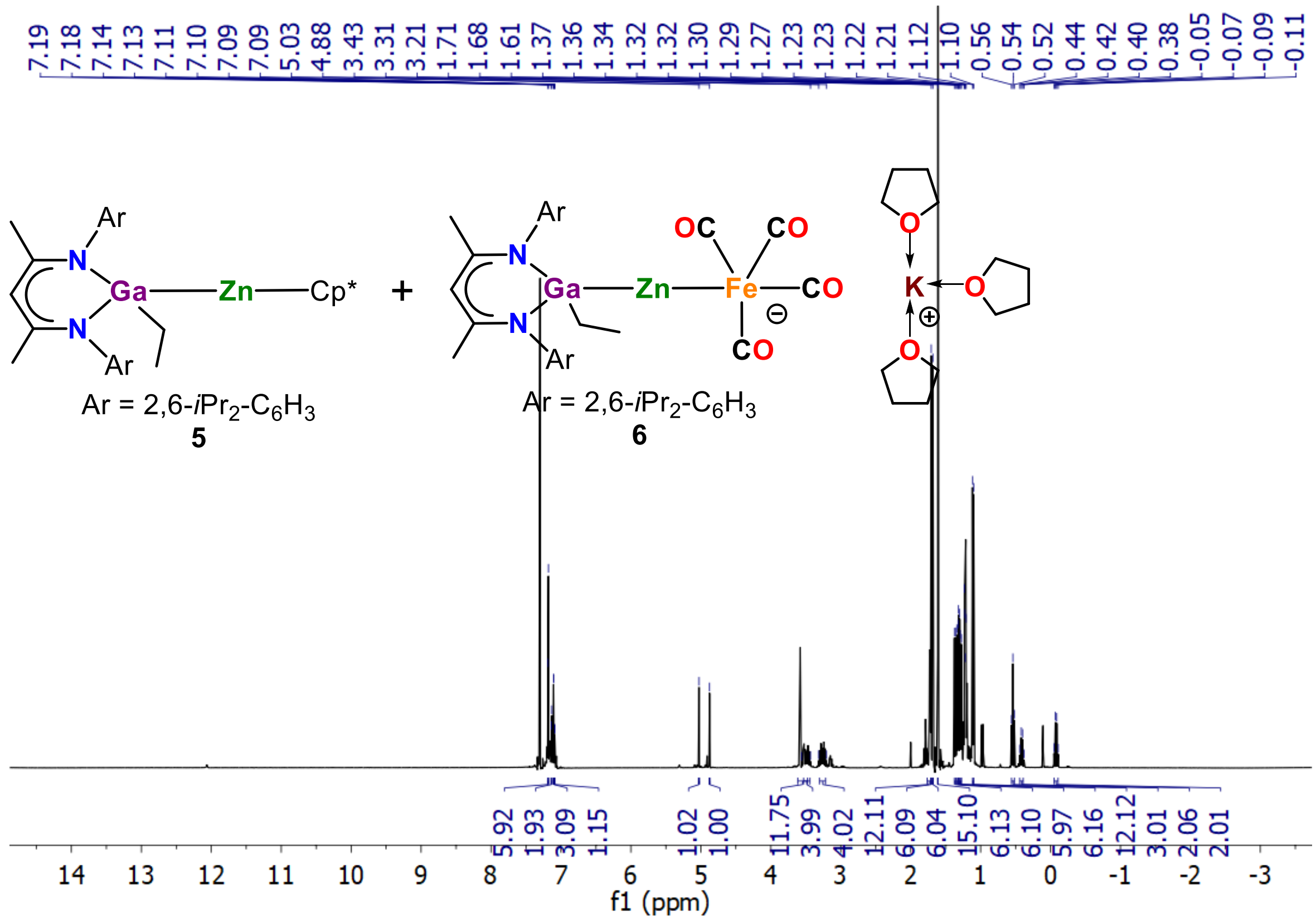

Figure S24. ^{1}H NMR (400 MHz, THF-d$_8$, 298 K) spectrum of compounds **5** and **6**.

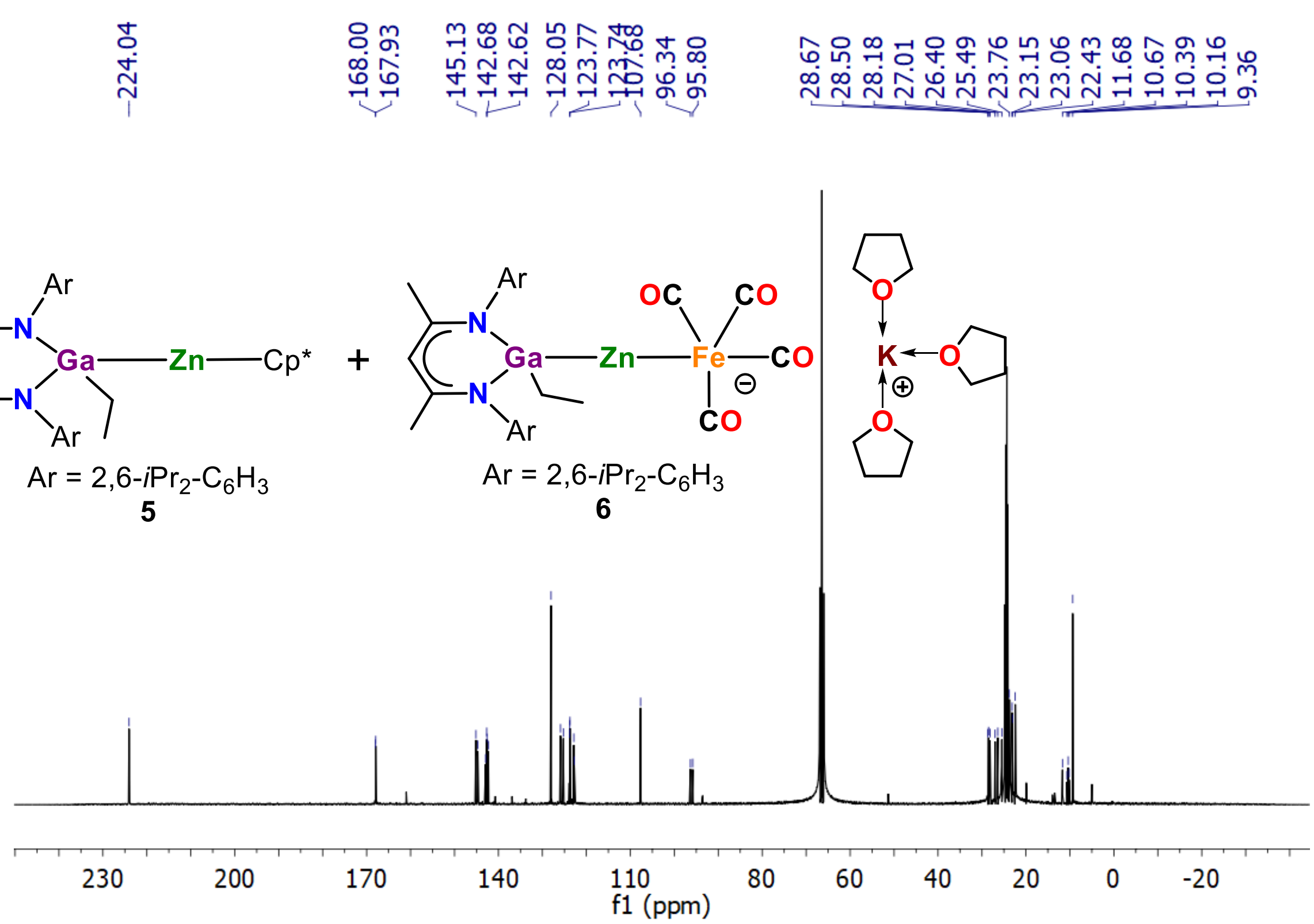

Figure S25. ^{13}C{^{1}H} NMR (100 MHz, THF-d$_8$, 298 K) spectrum of compounds **5** and **6**.

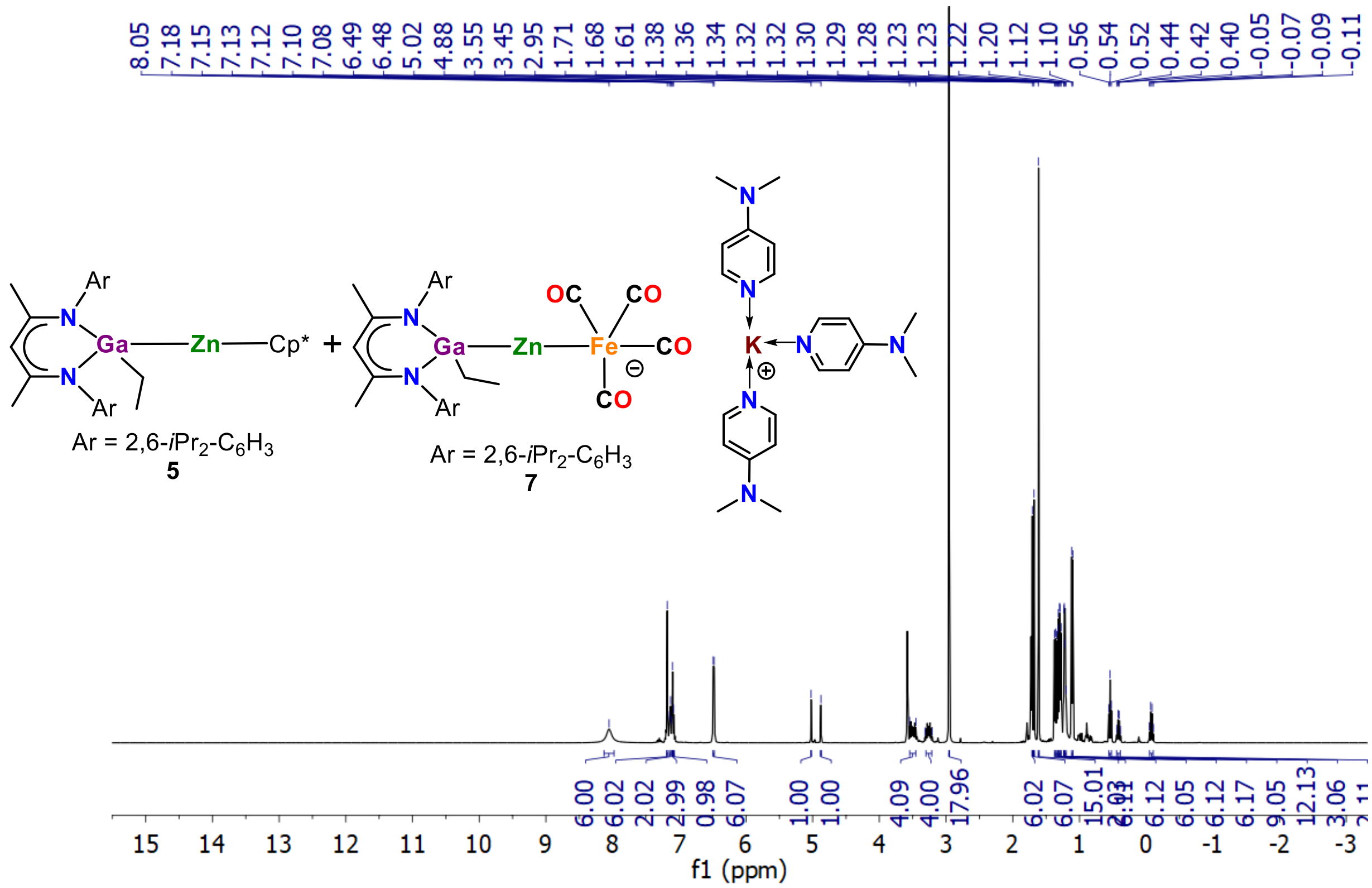

Figure S26. ^{1}H NMR (400 MHz, THF-d_8, 298 K) spectrum of compounds **5**, and **7**.

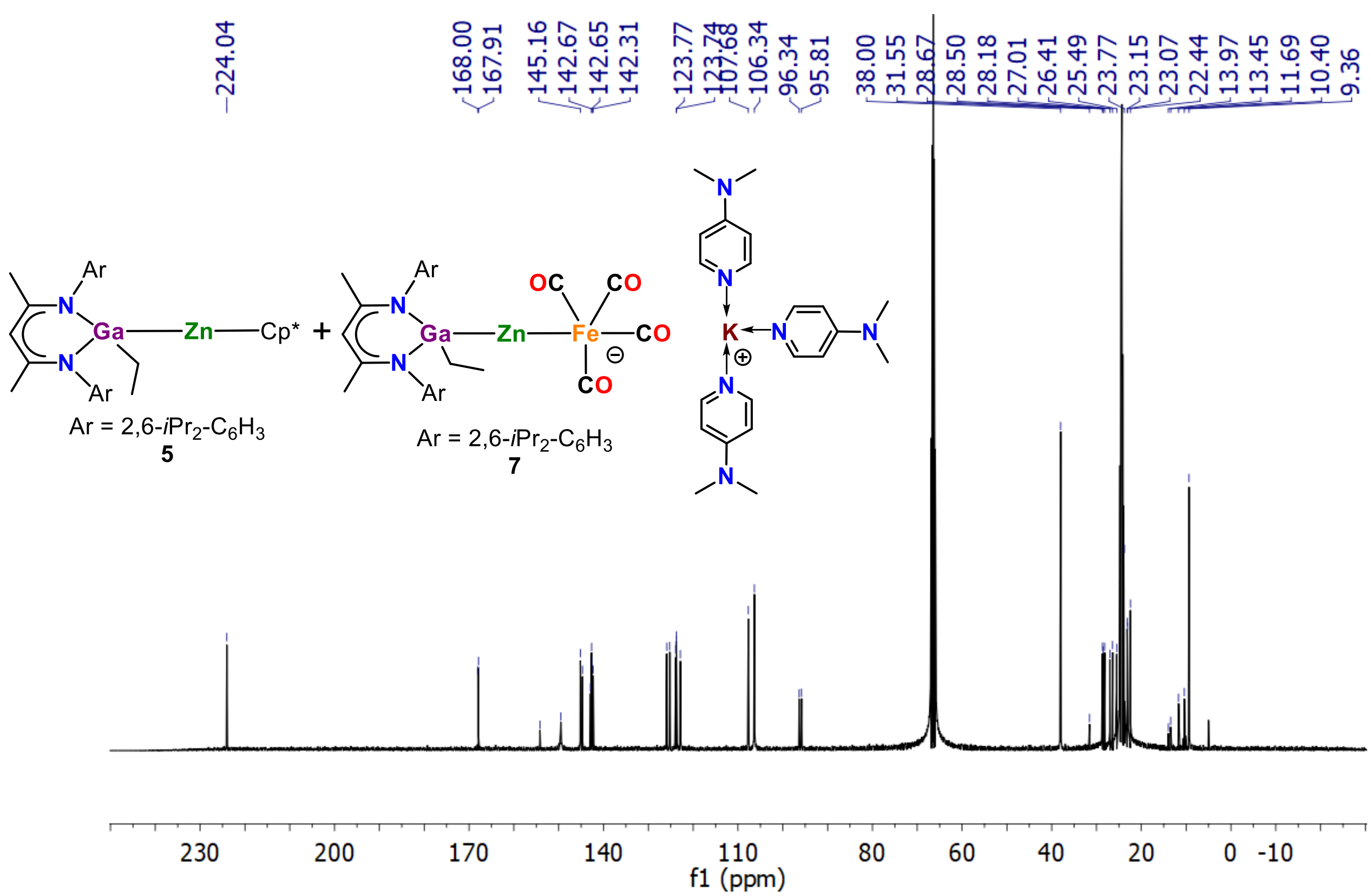

Figure S27. $^{13}C\{^{1}H\}$ NMR (100 MHz, THF-d_8, 298 K) spectrum of compounds **5**, and **7**.

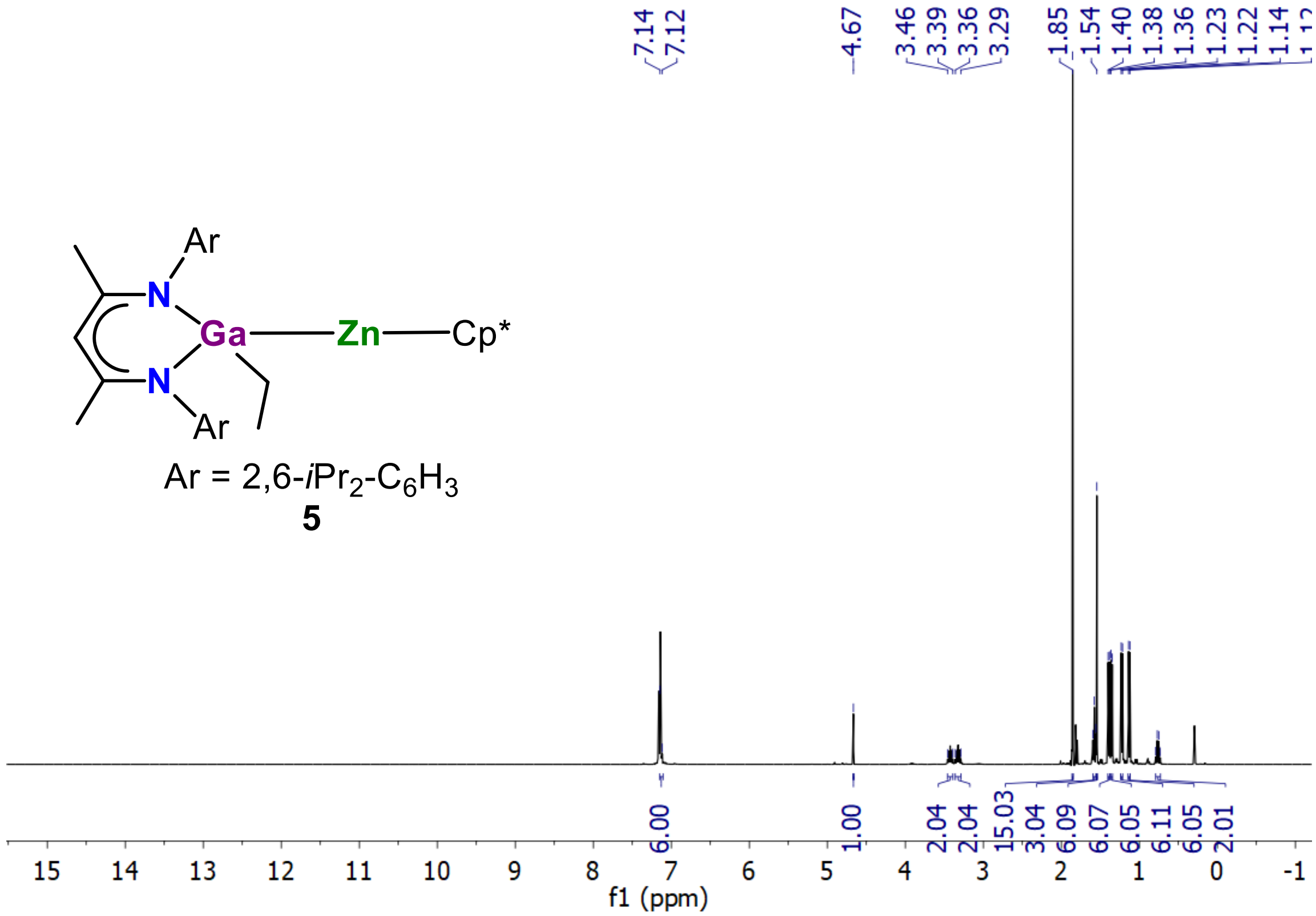

Figure S28. ^{1}H NMR (400 MHz, C_6D_6, 298 K) spectrum of compound **5**.

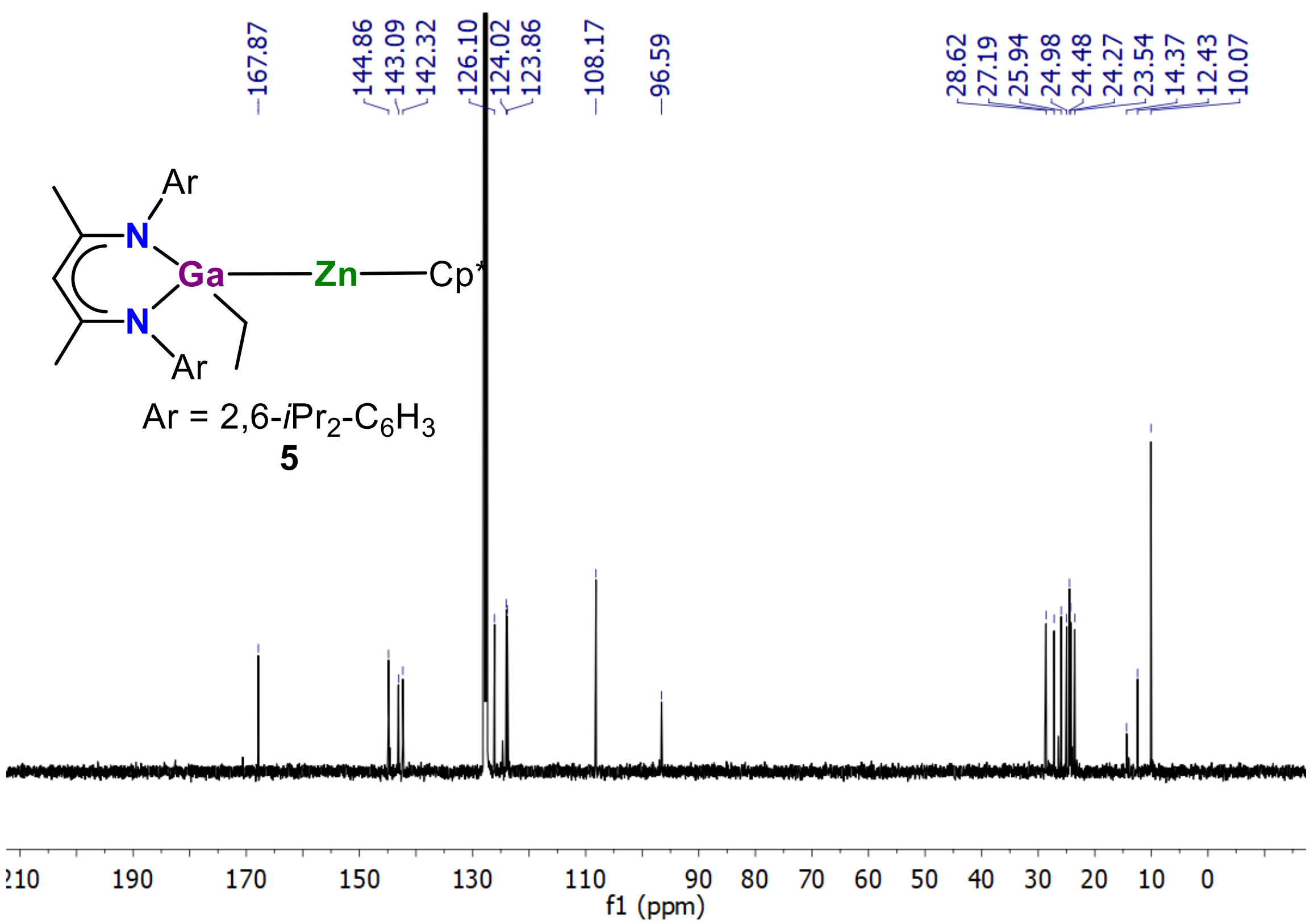

Figure S29. $^{13}C\{^{1}H\}$ NMR (100 MHz, C_6D_6, 298 K) spectrum of compound **5**.

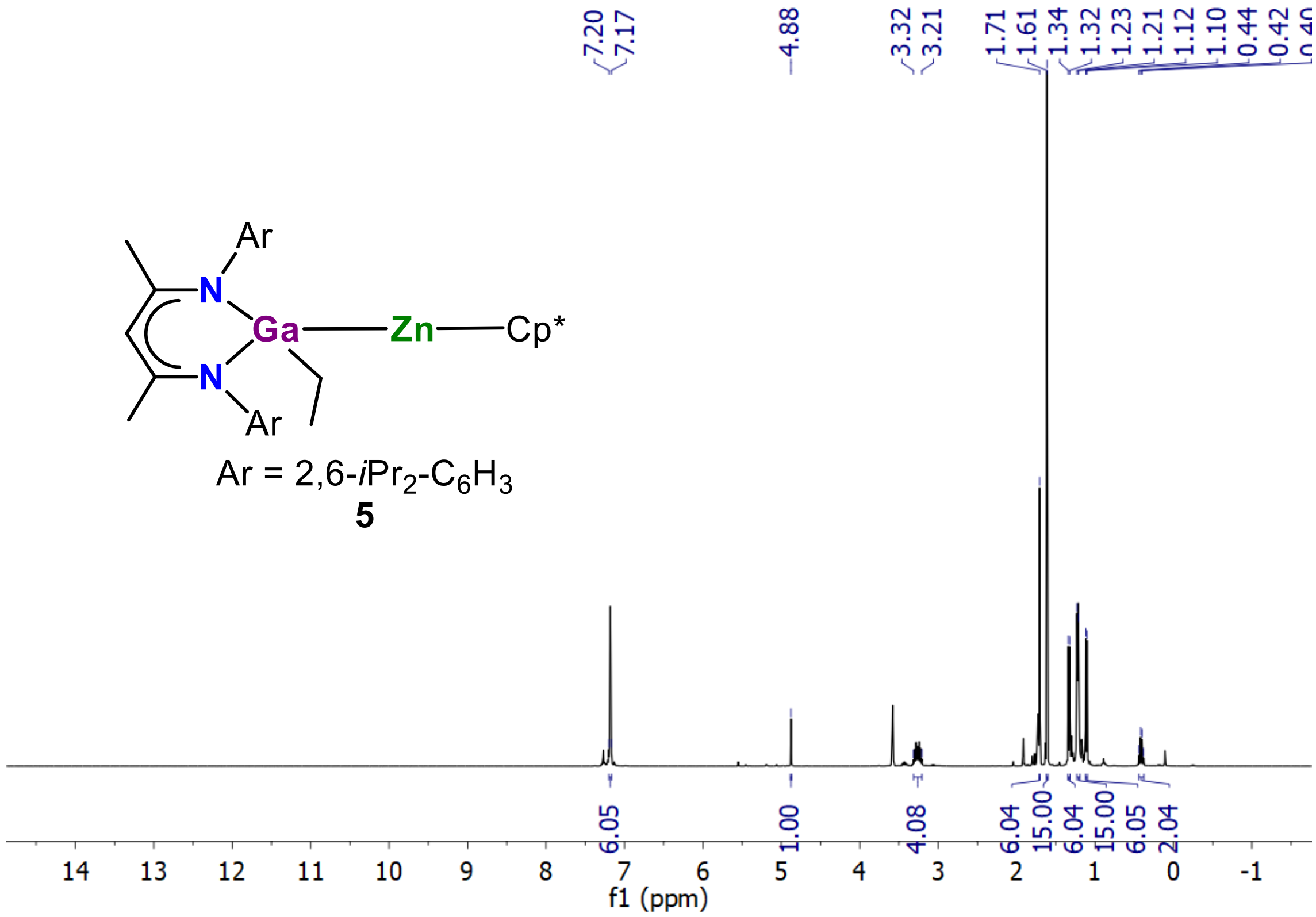

Figure S30. ^{1}H NMR (400 MHz, THF-d_8, 298 K) spectrum of compound **5**.

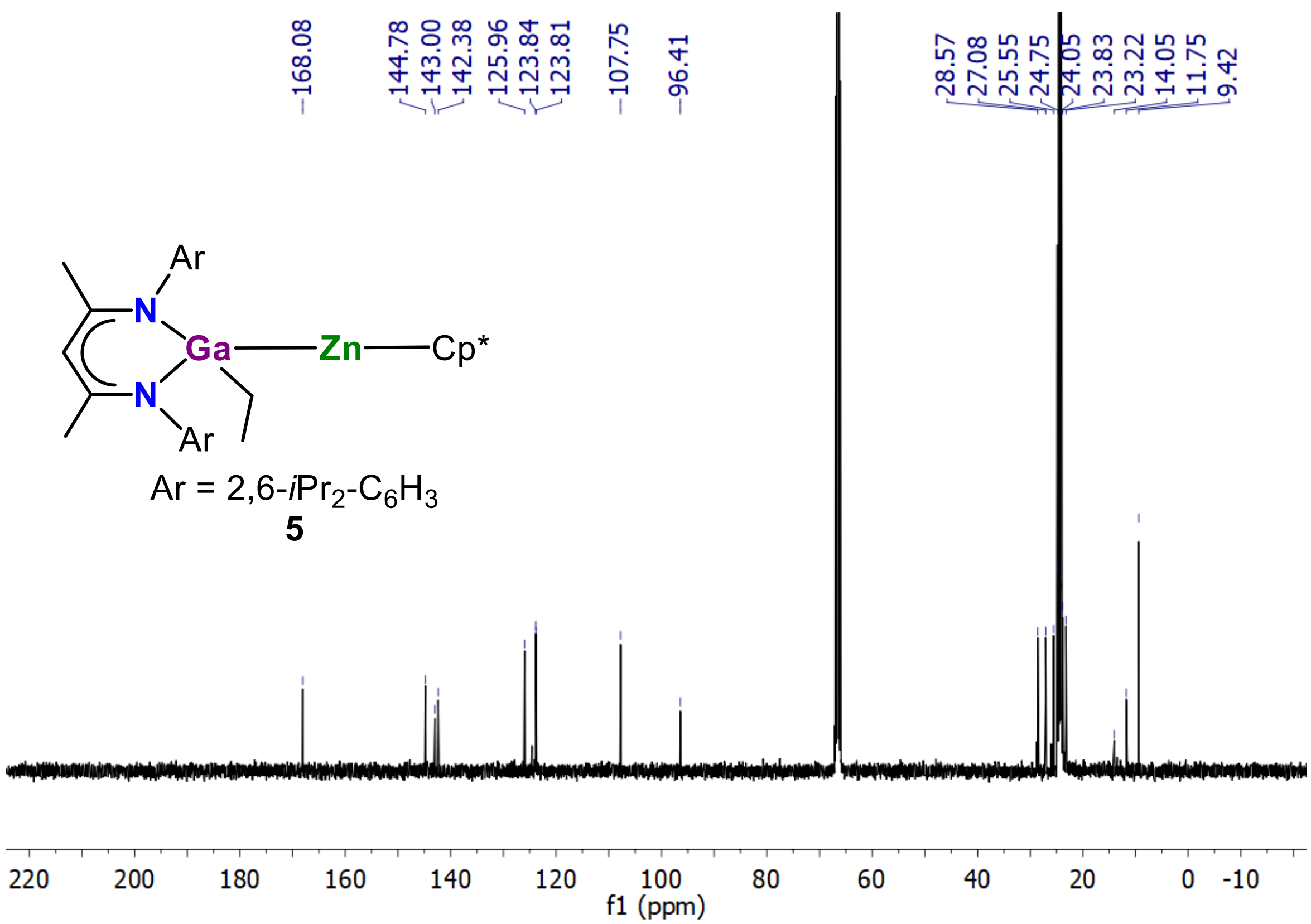

Figure S31. $^{13}C\{^{1}H\}$ NMR (100 MHz, THF-d_8, 298 K) spectrum of compound **5**.

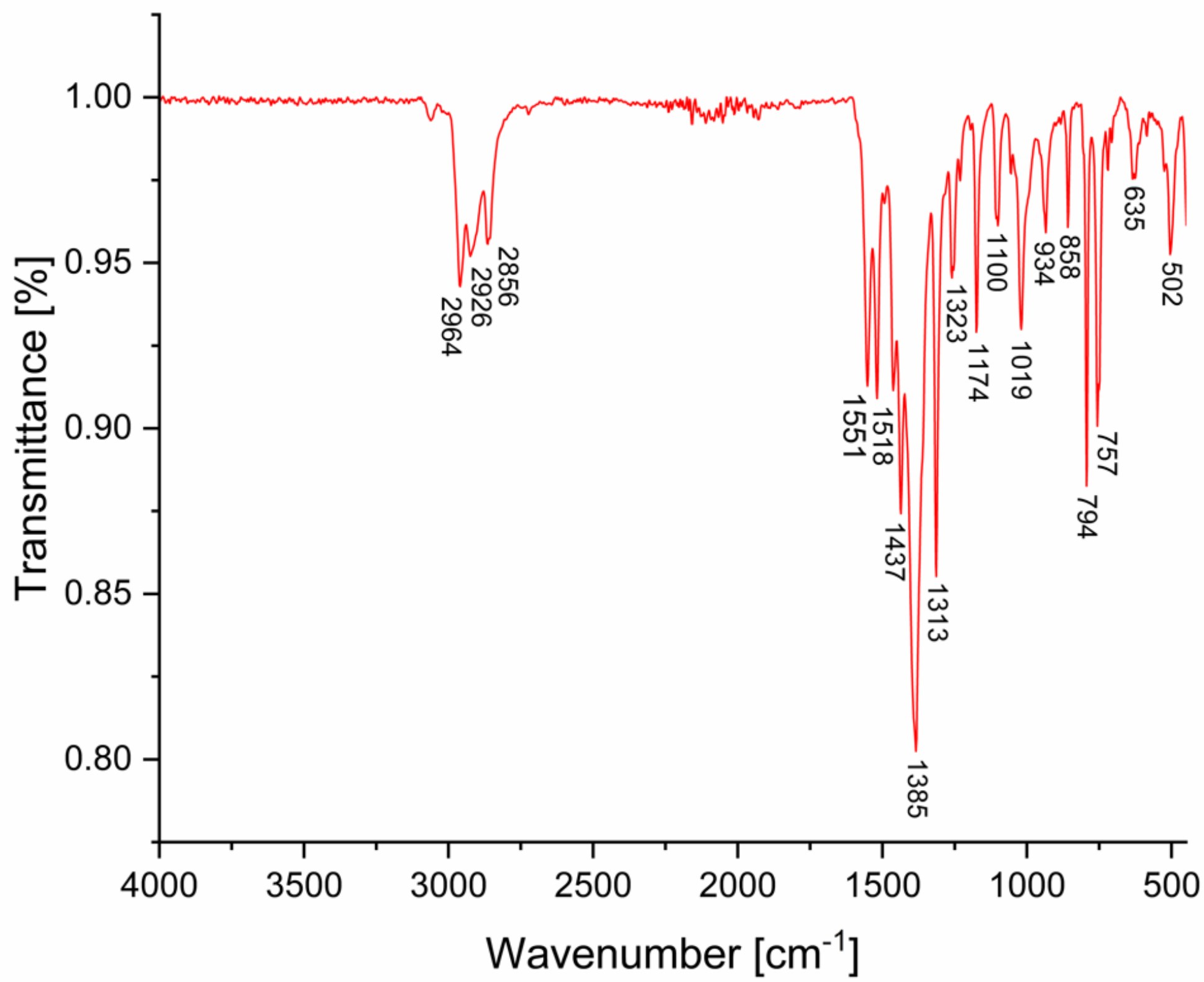

Figure S32. ATR-IR spectrum of compound **5**.

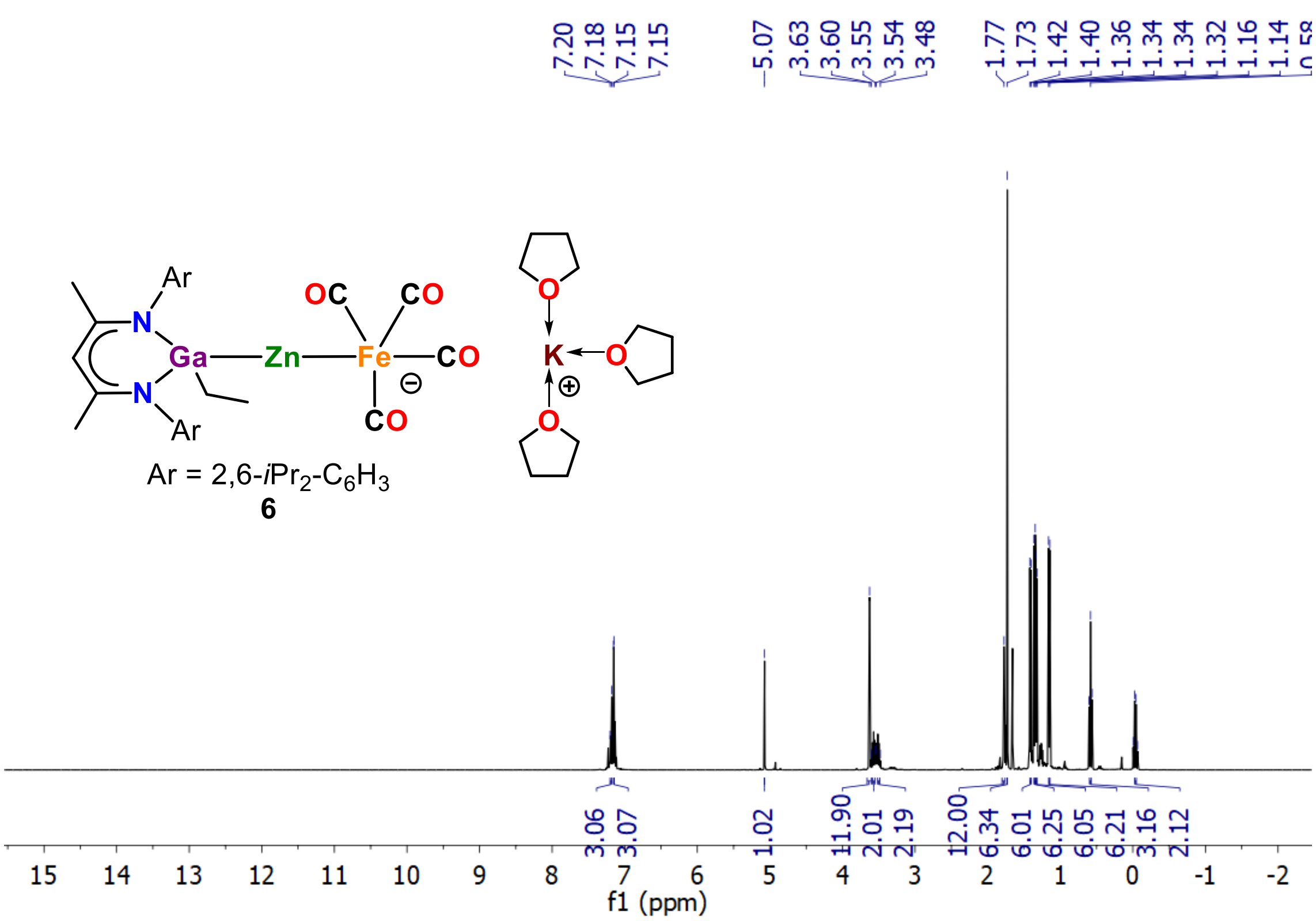

Figure S33. ^{1}H NMR (400 MHz, THF-d$_8$, 298 K) spectrum of compound **6**.

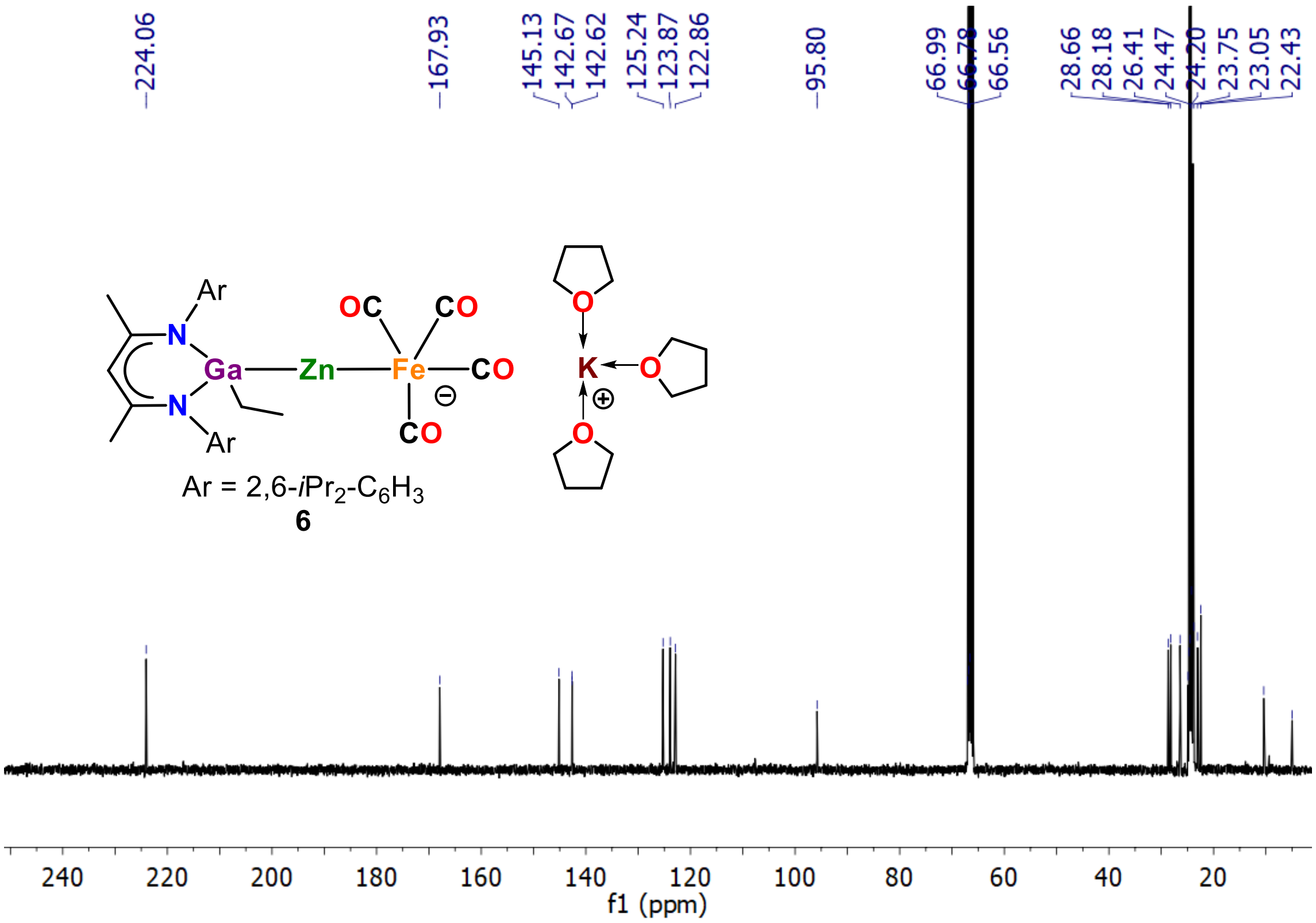

Figure S34. $^{13}C\{^{1}H\}$ NMR (100 MHz, THF-d_8, 298 K) spectrum of compound **6**.

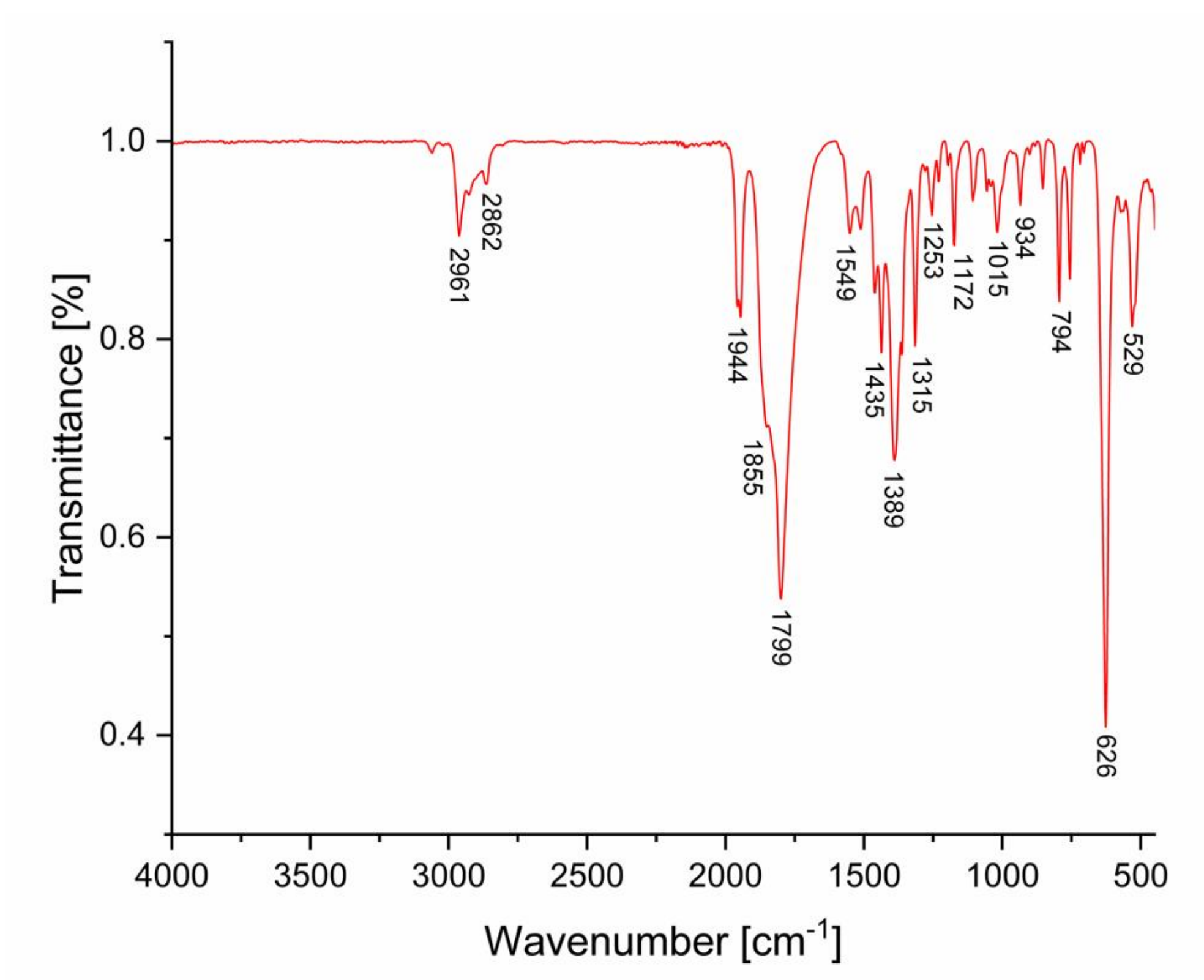

Figure S35. ATR-IR spectrum of compound **6**.

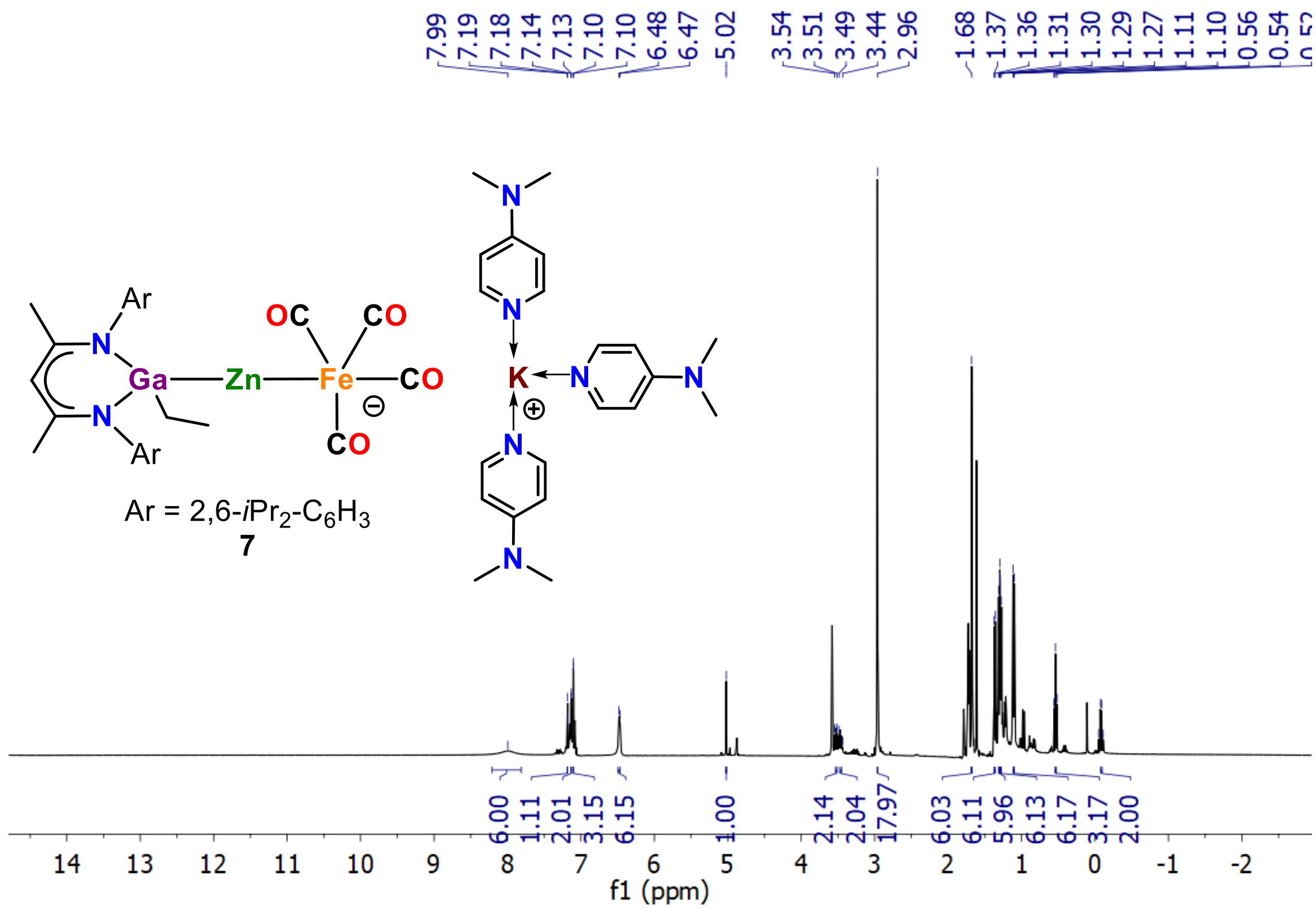

Figure S36. ^{1}H NMR (400 MHz, THF-d_8, 298 K) spectrum of compound **7**.

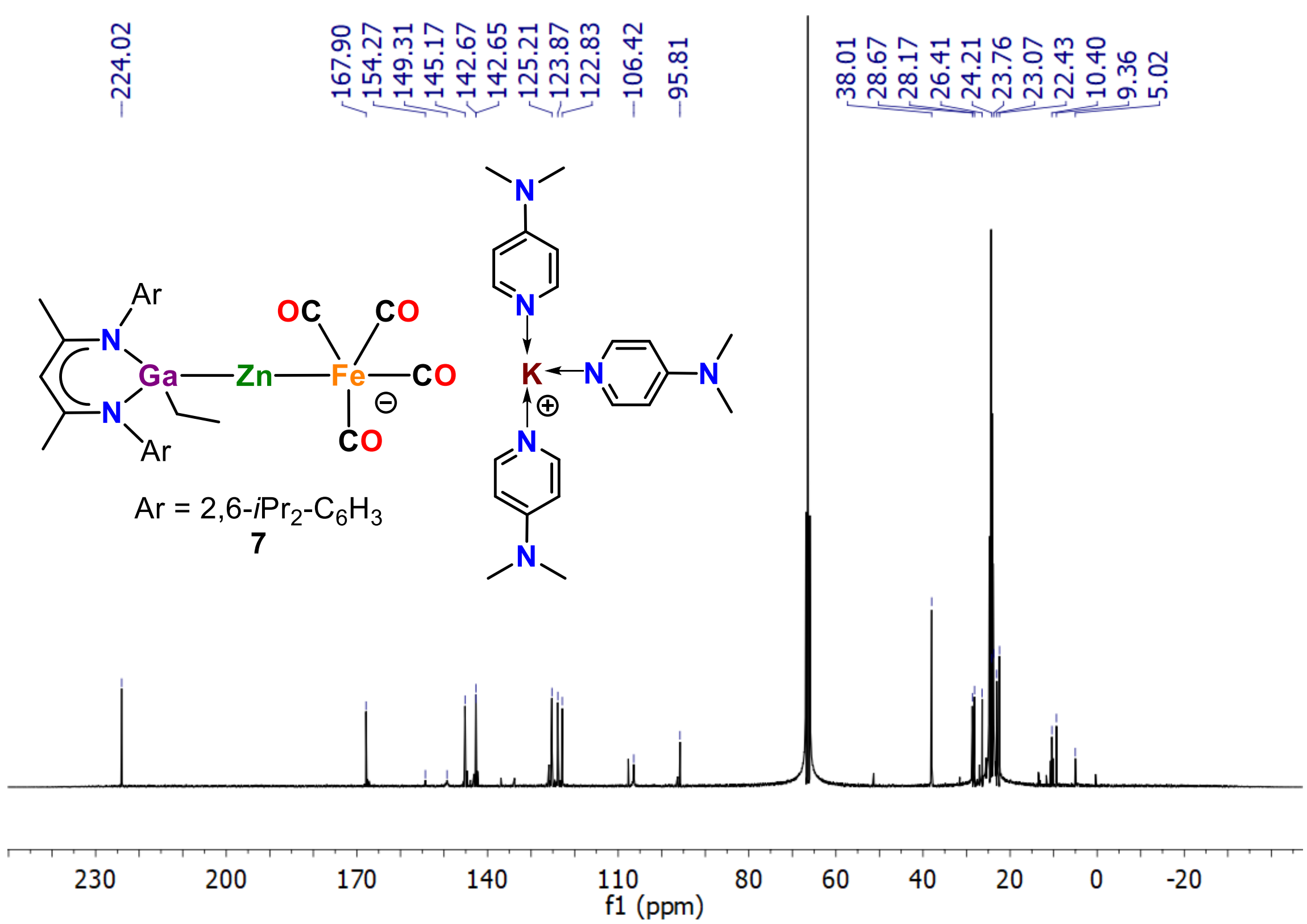

Figure S37. $^{13}C\{^{1}H\}$ NMR (100 MHz, THF-d_8, 298 K) spectrum of compound **7**.

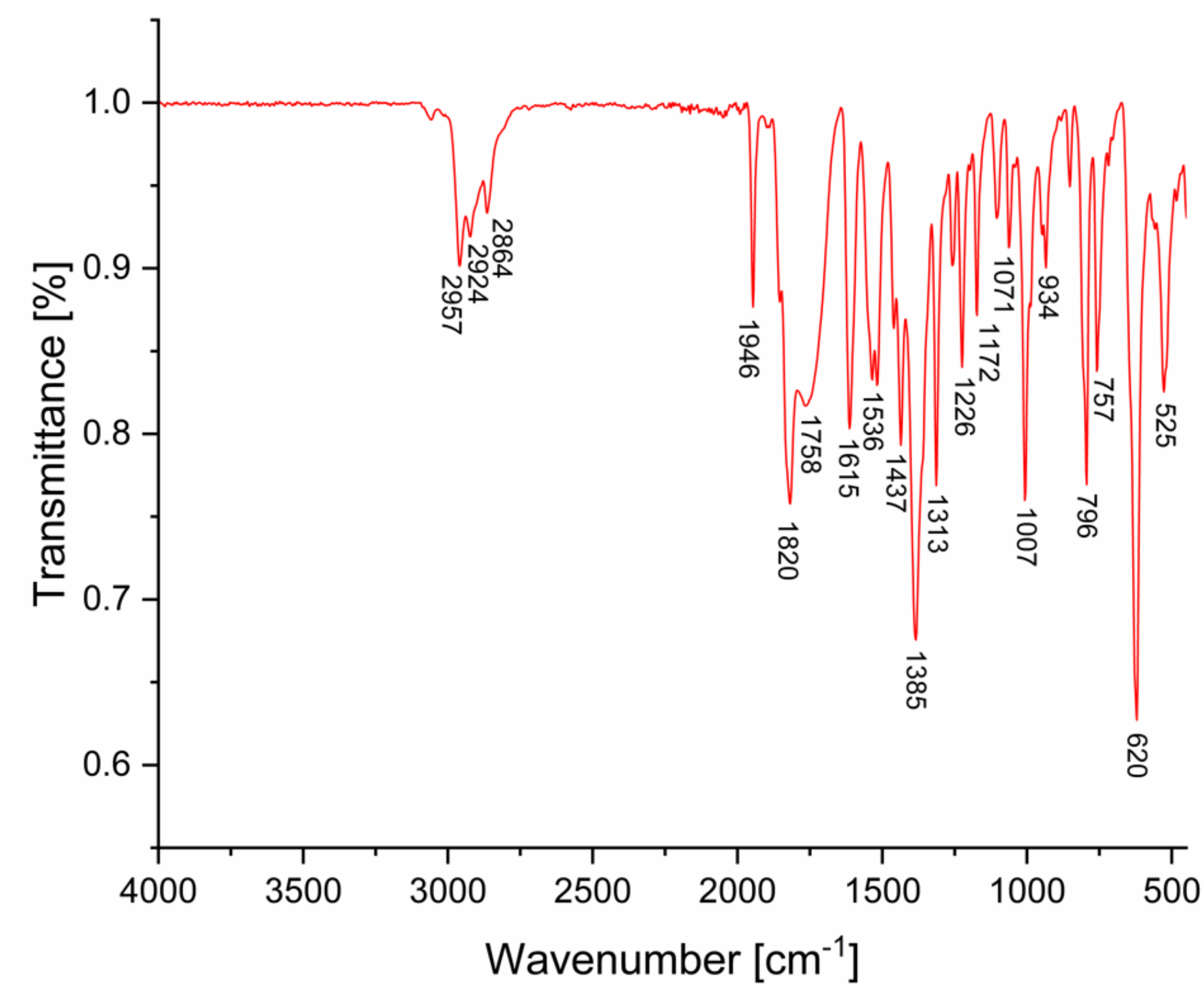

Figure S38. ATR-IR spectrum of compound **7**.

IV. Crystallographic Data of Complexes 1-5.

The crystals were mounted on nylon loops in inert oil. The data for **1-5** were collected on a Bruker AXS D8 Venture diffractometer with Photon II detector (monochromated $Cu_{K\alpha}$ radiation, λ = 1.54178 Å, microfocus source) each at 100(2) K. The structures were solved by Direct Methods (SHELXS-2013)[3] and refined anisotropically by full-matrix least-squares on F^2 (SHELXL-2017).[4-6] Absorption corrections were performed semi-empirically from equivalent reflections on basis of multi-scans (Bruker AXS APEX3). Hydrogen atoms were refined using a riding model or rigid methyl groups.

The crystal of compound **1** was a non-merohedral twin and the model was refined against de-twinned HKLF4 data.

On the crystal of **2** a small satellite crystal could not be removed. It diffracted too poorly to be treated as a twin domain, however due to overlaps reflection intensities may be distorted. Two obvious cases were ignored in the refinement (OMIT). Quantitative results should be carefully assessed.

In **3** one of the ^{t}Bu pyridine moieties is disordered over two positions. The corresponding bond lengths and angles of the ring were restrained to be equal (SADI) and the ring was restrained to be planar (FLAT). The displacement parameters were restrained with RIGU. Atoms in close proximity were refined with common displacement parameters (EADP). An isopropyl group is disordered over two positions. The solvent molecule is highly disordered and could only crudely be modelled with two alternate positions. The bond lengths and angles were restrained to be equal (SADI). An anisotropic refinement of the displacement parameters failed. Instead, they were constrained to equal values (EADP) to which RIGU restraints were applied. The co-ordinates of the alternate positions of the beginning and the end of the chain were constrained to be equal (EXYZ).

In **4** the solvent molecules and correlated to the THF on the centre of inversion an ethyl and isopropyl group are disordered. The *n*-hexane is only partially occupied and shares the position on the centre of inversion. The local symmetry of the molecules on the centre of inversion was ignored in the refinement (negative PART). The corresponding bond lengths and angles of the solvent molecules were restrained to be equal (SADI) and RIGU and SIMU restrained were used in the refinement of the displacement parameters. The displacement parameters of atoms in close proximity were refined with additional SIMU restraints to overcome parameter correlations. RIGU and SIMU restraints were applied to the displacement parameters of the isopropyl group.

The model of **5** was refined as a 2-component inversion twin. Even though the Flack parameter suggests centro-symmetry a solution in *Pnma* fails. The diffraction pattern suggests *c* to be doubled, however despite systematic extinctions of two glide planes a solution in orthorhombic space groups failed. The structure can be solved with this larger cell in monoclinic $P2_1/c$ with Z′ = 4 and a pseudo-merohedral twinning by 2-fold rotation about *a*. A refinement fails due to unacceptable displacement parameters. The final model (Z′ = 2)

shows pseudo translation which suggests that the true cell might be half the size (*a* half, $Z' = 1$) and the modulation extending in two directions. A solution with this cell in $Pna2_1$ (again $x \approx 0.5$) is possible but geometric and displacement parameter refine to unacceptable values. Considering this, results beyond the connectivity are unreliable and should not be discussed. A global RIGU restraint was applied and in two cases atoms related by the pseudo mirror plane were refined with common displacement parameters (EADP) to avoid non positive definite.

CCDC-2559142 (**1**), -2559143 (**2**), -2559144 (**3**), -2559145 (**4**), and -2559146 (**5**) contain the supplementary crystallographic data for this paper. These data can be obtained free of charge from The Cambridge Crystallographic Data Centre via www.ccdc.cam.ac.uk/data_request/cif.

Table S1. Crystal data and structure refinement of complexes **1-3**.

Compound	**1**	**2**	**3**
Empirical Formula	$C_{70}H_{108}Cl_2Ga_2N_4O_2Zn_2$	$C_{73}H_{100}FeGa_2N_4O_4Zn_2$	$C_{65}H_{92}FeGaN_5NaO_4Zn$
Formula weight	1378.68	1423.59	1221.36
Temperature (K)	100(2)	100(2)	100(2)
Wavelength (Å)	1.54178	1.54178	1.54178
Crystal size (mm)	0.323×0.130×0.084	0.174×0.145×0.079	0.418×0.297×0.209
Crystal system	triclinic	monoclinic	triclinic
Space group	*P*-1	*C*2/*c*	*P-1*
a (Å)	10.1070(12)	28.8218(16)	9.4658(3)
b (Å)	12.1933(14)	16.3271(9)	13.6690(4)
c (Å)	15.6285(18)	16.6588(9)	26.6127(8)
α (deg)°	108.416(3)	90	80.0814(8)
β (deg)°	96.254(3)	116.1389(18)	83.1620(9)
γ (deg)°	104.806(3)	90	83.8400(8)
Volume ($Å^3$)	1728.5(4)	7037.5(7)	3354.48(18)
Z	1	4	2
Calculated density (g/cm^3)	1.324	1.344	1.209
Absorption coefficient (mm^{-1})	2.725	3.609	3.017
F (000)	728	2984	1294
θ max (deg)°	80.470	79.495	79.598
Index ranges	$-12 \le h \le 12$<br>$-15 \le k \le 14$<br>$0 \le l \le 19$	$-36 \le h \le 36$<br>$-20 \le k \le 20$<br>$-21 \le l \le 21$	$-12 \le h \le 11$<br>$-17 \le k \le 17$<br>$-33 \le l \le 33$
No. of reflections collected	176970	112198	155803
Unique reflect.	7508	7597	14440
R_{int}	0.0520	0.0621	0.0373
Data / restraints / parameters	7357 / 0 / 381	7597 / 0 / 404	14440 / 527 / 818
Goodness–of–fit on F^2	1.035	1.039	1.026
$R1$ [$I>2\sigma(I)$]	0.0230	0.0278	0.0269
$wR2$ [$I>2\sigma(I)$]	0.0629	0.0743	0.0725
$R1$ [all data]	0.0234	0.0300	0.0269
$wR2$ [all data]	0.0632	0.0761	0.0726
Flack parameter	-	-	-
Largest diff. peak and hole, max./min.[$e \cdot Å^{-3}$]	0.329/-0.453	0.868/-0.403	0.676/-0.619

Table S2. Crystal data and structure refinement of complexes **4** and **5**.

Compound	**4**	**5**
Empirical Formula	$C_{93.09}H_{134.68}FeGa_2N_{10}O_{5.15}Zn_2$	$C_{41}H_{61}GaN_2Zn$
Formula weight	1802.29	717.00
Temperature (K)	100(2)	100(2)
Wavelength (Å)	1.54178	1.54178
Crystal size (mm)	0.355×0.265×0.072	0.340×0.244×0.198
Crystal system	triclinic	orthorhombic
Space group	*P*-1	$Pna2_1$
a (Å)	12.0711(6)	35.1185(18)
b (Å)	18.1583(10)	20.6610(11)
c (Å)	21.3071(11)	10.6986(5)
α (deg)°	92.9371(18)	90
β (deg)°	97.2064(18)	90
γ (deg)°	91.8977(17)	90
Volume ($Å^3$)	4623.6(4)	7762.7(7)
Z	2	8
Calculated density (g/cm^3)	1.295	1.227
Absorption coefficient (mm^{-1})	2.886	1.809
F (000)	1905	3056
θ max (deg)°	80.282	79.530
Index ranges	$-15 \leq h \leq 15$<br>$-23 \leq k \leq 23$<br>$-27 \leq l \leq 27$	$-44 \leq h \leq 44$<br>$-24 \leq k \leq 26$<br>$-13 \leq l \leq 13$
No. of reflections collected	276709	413148
Unique reflect.	20027	16785
R_{int}	0.0432	0.0605
Data / restraints / parameters	19550 / 433 / 1216	16785 / 799 / 832
Goodness–of–fit on F^2	1.040	1.069
$R1$ [I>2σ(I)]	0.0284	0.0689
$wR2$ [I>2σ(I)]	0.0744	0.1646
$R1$ [all data]	0.0290	0.0696
$wR2$ [all data]	0.0748	0.1650
Flack parameter	-	0.46(5)
Largest diff. peak and hole, max./min.[$e \cdot Å^{-3}$]	0.697/-0.877	2.088/-3.534

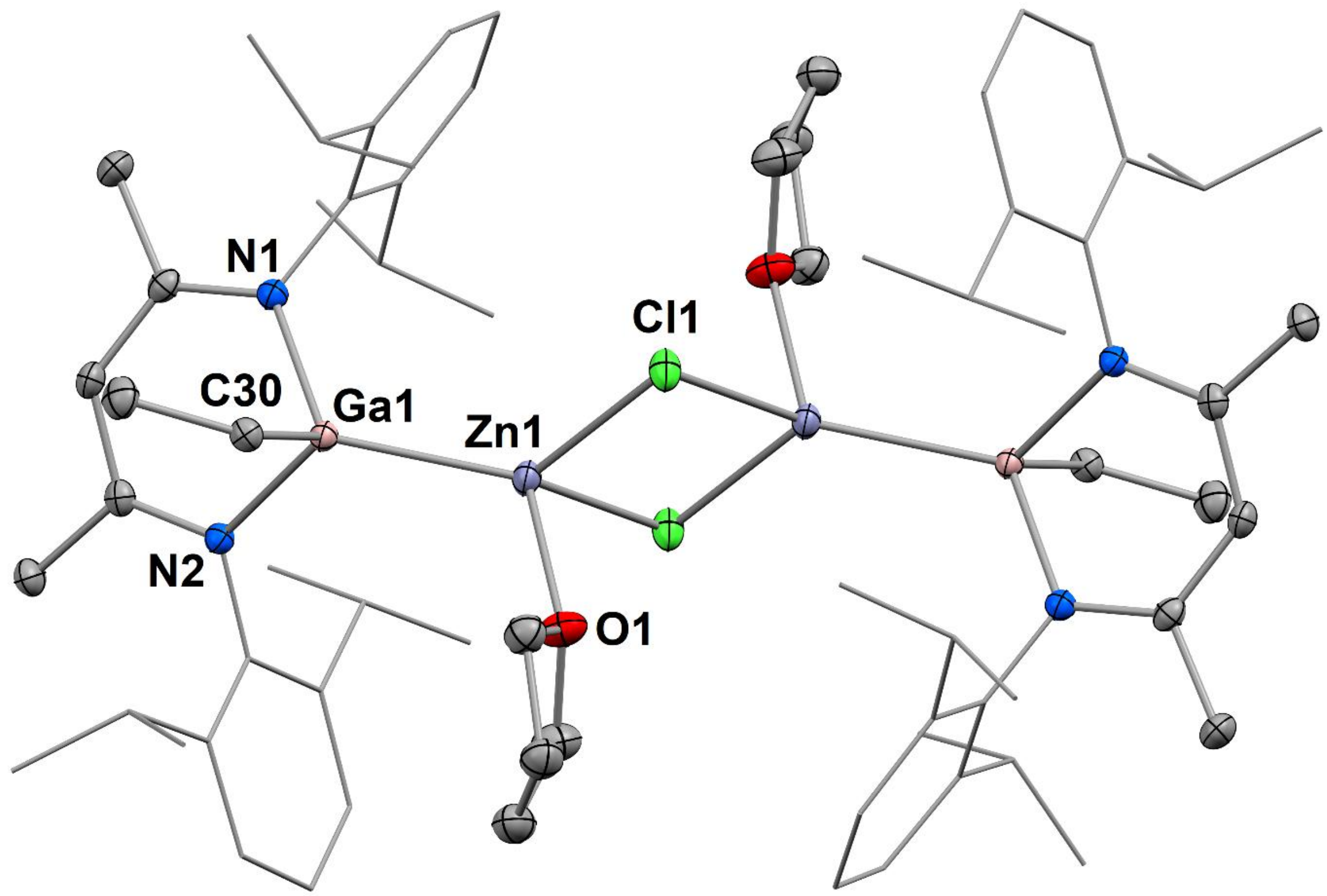

Figure S39: Molecular structure of **1**. Displacement ellipsoids drawn at 50% probability level, and hydrogen atoms are omitted for clarity. Labels are omitted on symmetry generated atoms. Selected distances (Å) and angles (°) for **1**: Ga1-Zn1 2.4089(3), Ga1-N1 2.0013(10), Ga1-N2 2.0316(10), Zn1-Cl1 2.3570(4), Ga1-C30 2.0325(12), Zn1-O1 2.1571(10), N1-Ga1-Zn1 116.74(3), N2-Ga1-Zn1 117.21(3), N1-Ga1-N2 92.14(4), Cl1-Zn1-Ga1 138.262(11), Cl1-Zn1-O1 91.46(3).

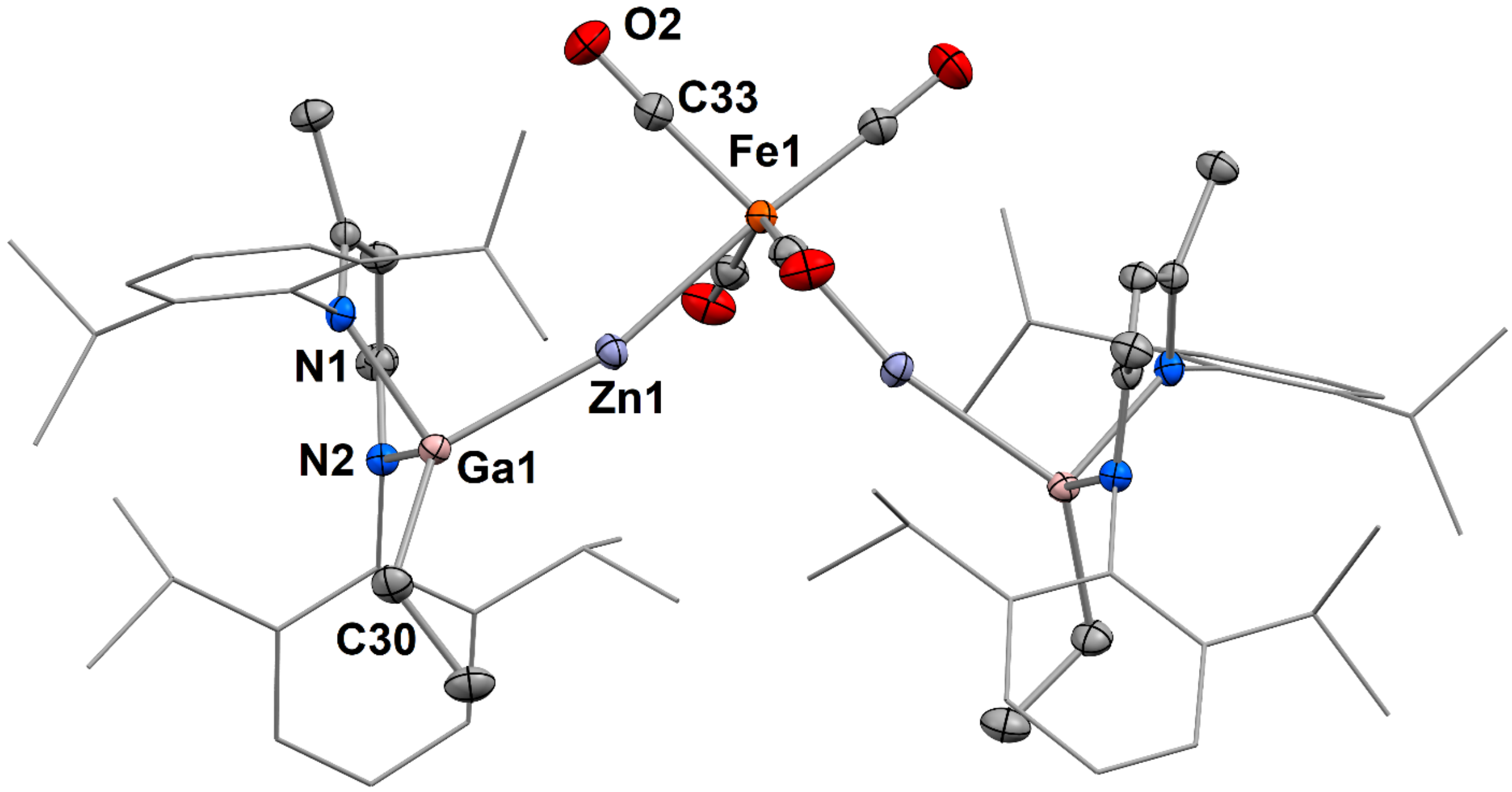

Figure S40: Molecular structure of **2**. Displacement ellipsoids drawn at 50% probability level, and hydrogen atoms are omitted for clarity. Labels are omitted on symmetry generated atoms. Selected distances (Å) and angles (°) for **2**: Ga1-Zn1 2.4336(3), Zn1-Fe1 2.4386(3), Fe1-C33 1.7760(17), C33-O2 1.147(2), Ga1-N1 1.9944(13), Ga1-N2 1.9905(13), Ga1-C30 1.9768(17), Ga1-Zn1-Fe1 166.904(12), Zn1-Fe1-Zn1’ 90.287(16), N1-Ga1-Zn1 97.25(4), N2-Ga1-Zn1 99.27(4), N1-Ga1-N2 92.82(5).

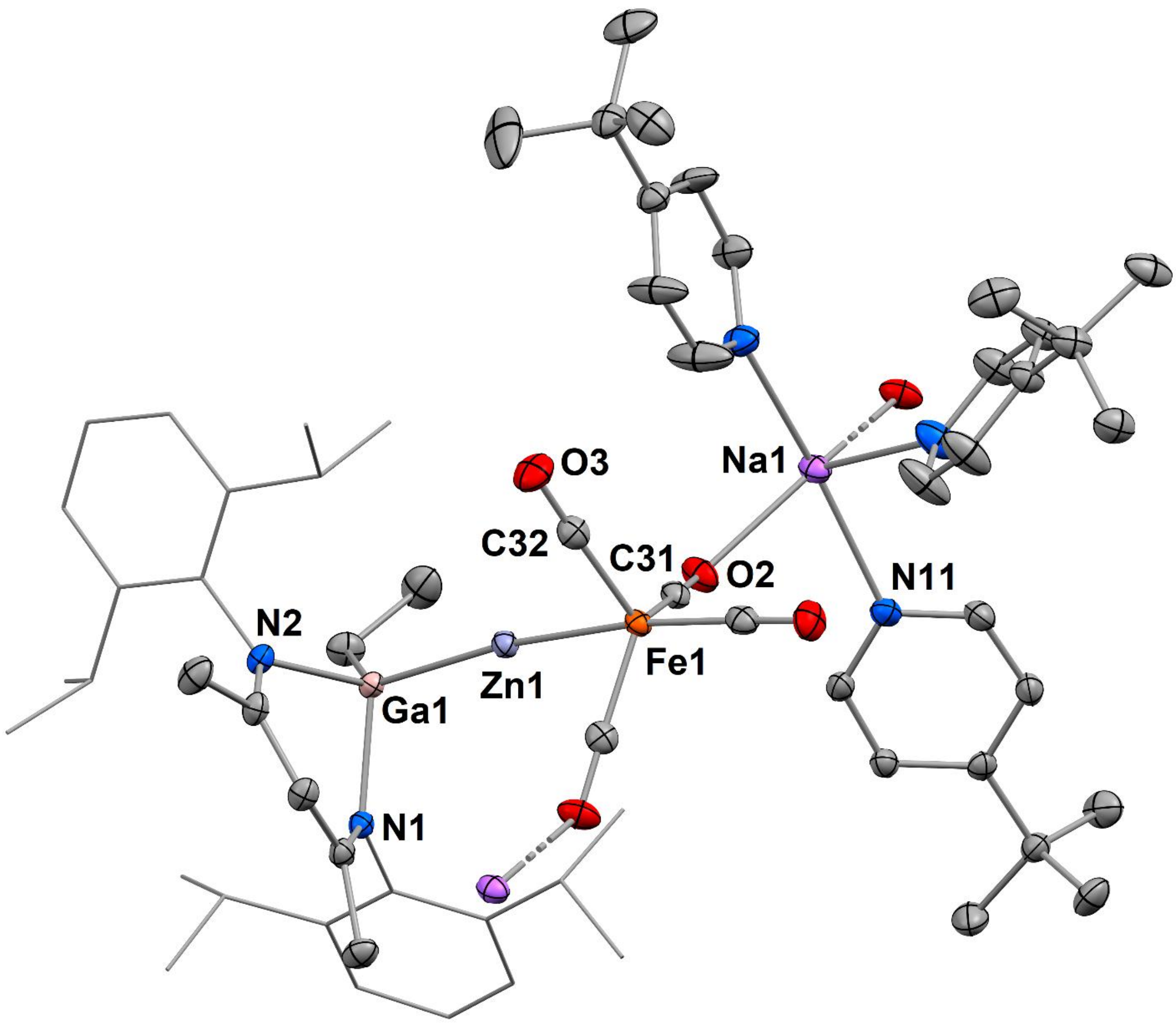

Figure S41: Molecular structure of **3**. Displacement ellipsoids drawn at 50% probability level, and hydrogen atoms are omitted for clarity. Selected distances (Å) and angles (°) for **3**: Ga1-Zn1 2.4303(2), Zn1-Fe1 2.3723(2), Fe1-C31 1.7553(13), Fe1-C32 1.7729(14), C31-O2 1.1753(16), C32-O3 1.1609(17), Na1-O2 2.3462(10), Na1-N11 2.4265(12), Ga1-C34 1.9892(13), Ga1-N1 2.0074(10), Ga1-N2 2.0125(10), Ga1-Zn1-Fe1 173.535(9), Zn1-Fe1-C30 173.22(4), Zn1-Fe1-C31 74.42(4), Fe1-C31-O2 179.08(13), C31-O2-Na1 130.81(9), N1-Ga1-Zn1 103.85(3), N2-Ga1-Zn1 102.67(3), N1-Ga1-N2 92.77(4), C34-Ga1-Zn1 128.82(4).

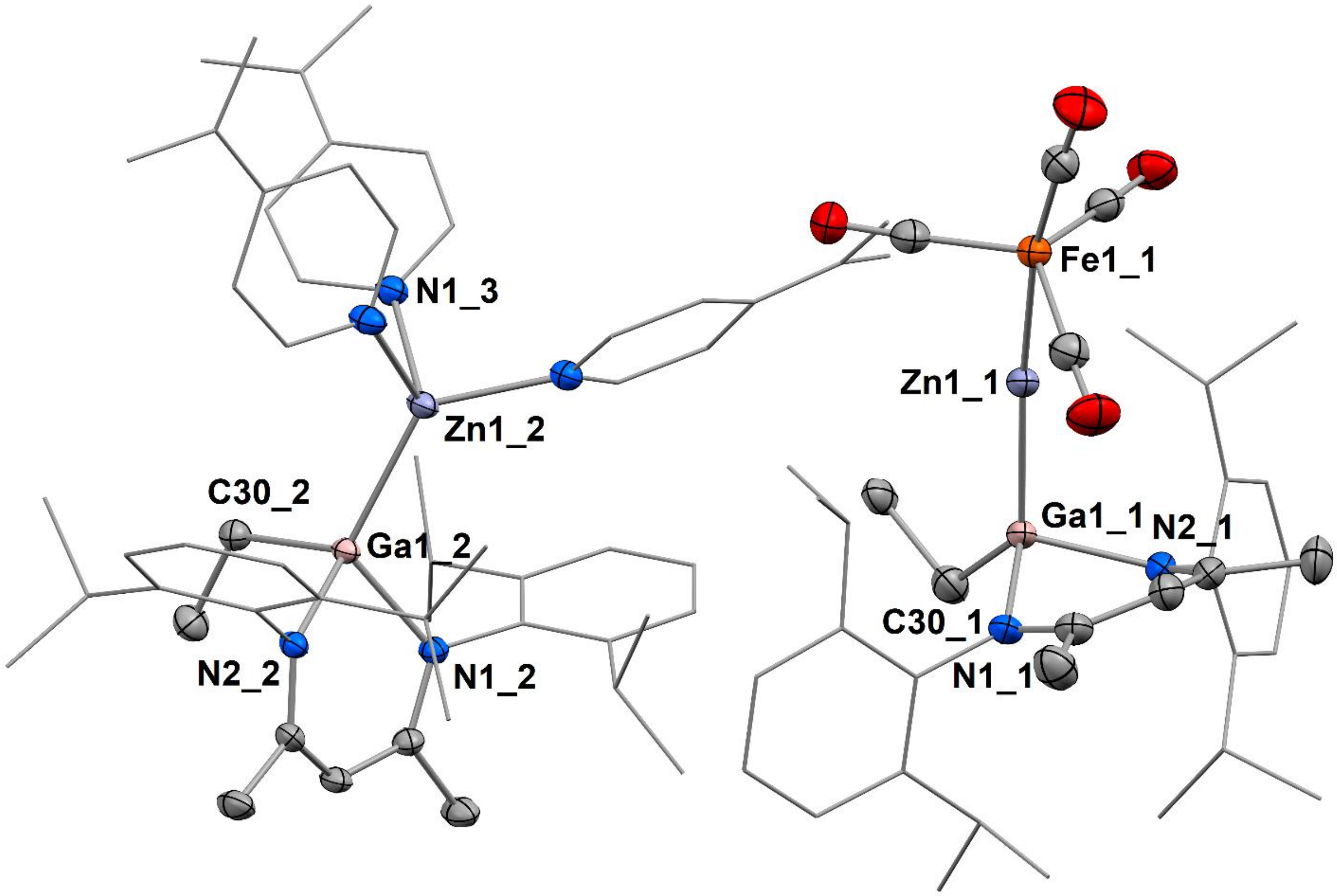

Figure S42: Molecular structure of **4**. Displacement ellipsoids drawn at 50% probability level, and hydrogen atoms are omitted for clarity. Selected distances (Å) and angles (°) for **4**: Ga11-Zn11 2.4508(3), Zn11-Fe11 2.3682(3), Ga12-Zn12 2.4150(3), Zn12-N13 2.1646(12, Ga11-N11 2.0257(13), Ga11-N21 2.0106(13), Ga12-N12 2.0266(11), Ga12-N22 2.0383(12), Ga11-C301 1.9815(18), Ga12-C302 2.0259(15), Ga11-Zn11-Fe11 169.657(12), N11-Ga11-Zn11 101.85(4), N21-Ga11-Zn11 103.71(4), N21-Ga11-N11 91.27(5), C301-Ga11-Zn11 134.35(5), C301-Ga11-Zn11 134.35(5), N12-Ga12-N22 92.35(5), N12-Ga12-Zn12 115.31(3), N22-Ga12-Zn12 120.50(3), C302-Ga12-Zn12 115.86(4), N13-Zn12-Ga12 110.33(3).

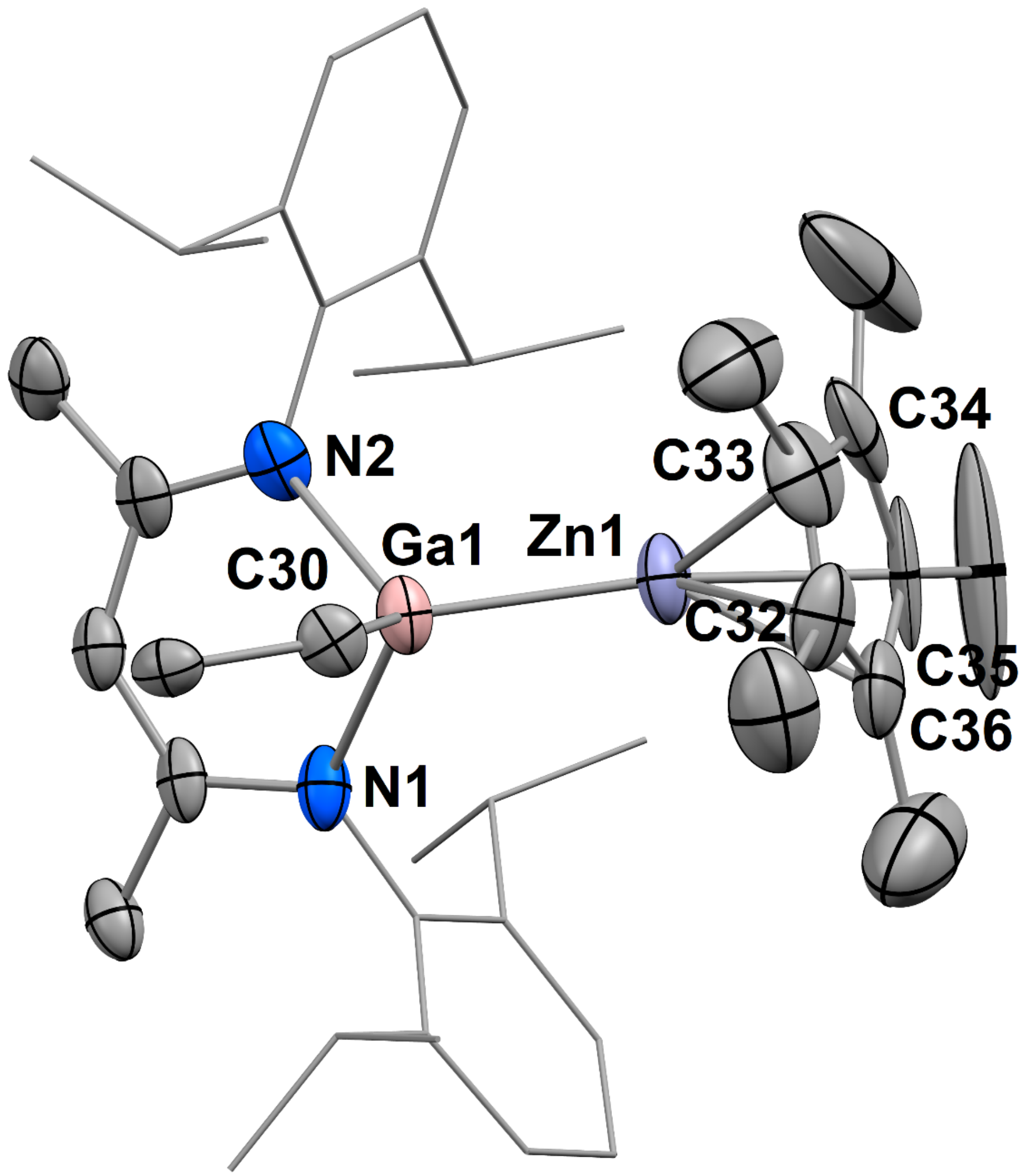

Figure S43: Molecular structure of **5**. Displacement ellipsoids drawn at 50% probability level, and hydrogen atoms are omitted for clarity. Selected distances (Å) and angles (°) for **5**: Ga1−Zn1 2.3685(14), Ga1−C30 1.968(9), Ga1−N1 2.011(5), Ga1−N2 1.986(5), Zn1−C32 2.305(9), Zn1−C33 2.357(9), Zn1−C34 2.289(8), Zn1−C35 2.265(9), Zn1−C36 2.251(11), N1−Ga1−Zn1 113.79(16), N2−Ga1−Zn1 115.51(16), N1−Ga1−N2 94.1(2), C30−Ga1−Zn1 114.6(3), Cp*(centr)-Zn1-Ga1 171.0(3).

V. Computational Details

All calculations were performed by using the programs Gaussian 16[7] and the Amsterdam Modeling Suite (AMS; Amsterdam Density Functional (ADF)).[8-9] The geometrical parameters of the neutral molecule **2** and the anionic part of **3** were optimized by means of the density functionals PBE0[10], the dispersion corrections with Becke-Johnson damping (D3BJ)[11] and the basis set def2-SVP[12-13]. For all structures C_1 symmetry was applied. Frequency calculations were carried out at each of the structures to verify the nature of the stationary point. It turned out that all structures have none imaginary frequency.

To investigate the possible presence of multireference effects, state-averaged CASSCF (Complete Active Space Self-Consistent Field) calculations were performed using ORCA 5.[14] The def2-SVP basis set was employed. Three singlet states were included in the state-averaged treatment. An active space comprising eight electrons in eight orbitals was used. Analysis of the CASSCF wavefunction revealed that the ground state is predominantly described by a single electronic configuration, with the 22220000 occupation pattern exhibiting a CI coefficient of 0.96. This indicates negligible multireference character and supports the applicability of single-reference DFT methods for the present system.

Furthermore, the energies of the stationary points were calculated using the density functional PBE0-D3BJ and the basis set def2-TZVP[12]. To take solvent effects into account, the solvent model SMD[15] (benzene as solvent) was used for the single point calculations. The bond energy analysis calculations[16] and extended transition state–natural orbitals for chemical valence (ETS-NOCV)[17] analysis were performed by means of the density functional B3LYP[18-20] with additional dispersion correction with Becke-Johnson damping[11] and the basis set TZ2P[21]. To treat relativistic effects the zeroth order regular approximation (ZORA)[22] to the Dirac equation was used for the bond energy analysis

Table S3. NBO charges [e] calculated by means of PBE0-D3BJ(SMD,C_6H_6)/def2-TZVP.

Compound	Ga(1)	Zn(1)	Fe	Zn(2)	Ga(2)
**2**	0.923	0.672	-2.821	0.672	0.923
Anionic part of **3**	0.866	0.717	-2.632		

Table S4. Wiberg bond index calculated by means of PBE0-D3BJ(SMD,C_6H_6)/def2-TZVP.

Compound	Ga(1)–Zn(1)	Zn(1)–Fe	Fe–Zn(2)	Zn(2)–Ga(2)
**2**	0.804	0.252	0.252	0.804
Anionic part of **3**	0.770	0.353		

Table S5. Mayer bond order calculated by means of PBE0-D3BJ(SMD,C_6H_6)/def2-TZVP.

Compound	Ga(1)–Zn(1)	Zn(1)–Fe	Fe–Zn(2)	Zn(2)–Ga(2)
**2**	0.690	0.617	0.617	0.691
Anionic part of **3**	0.801	0.666		

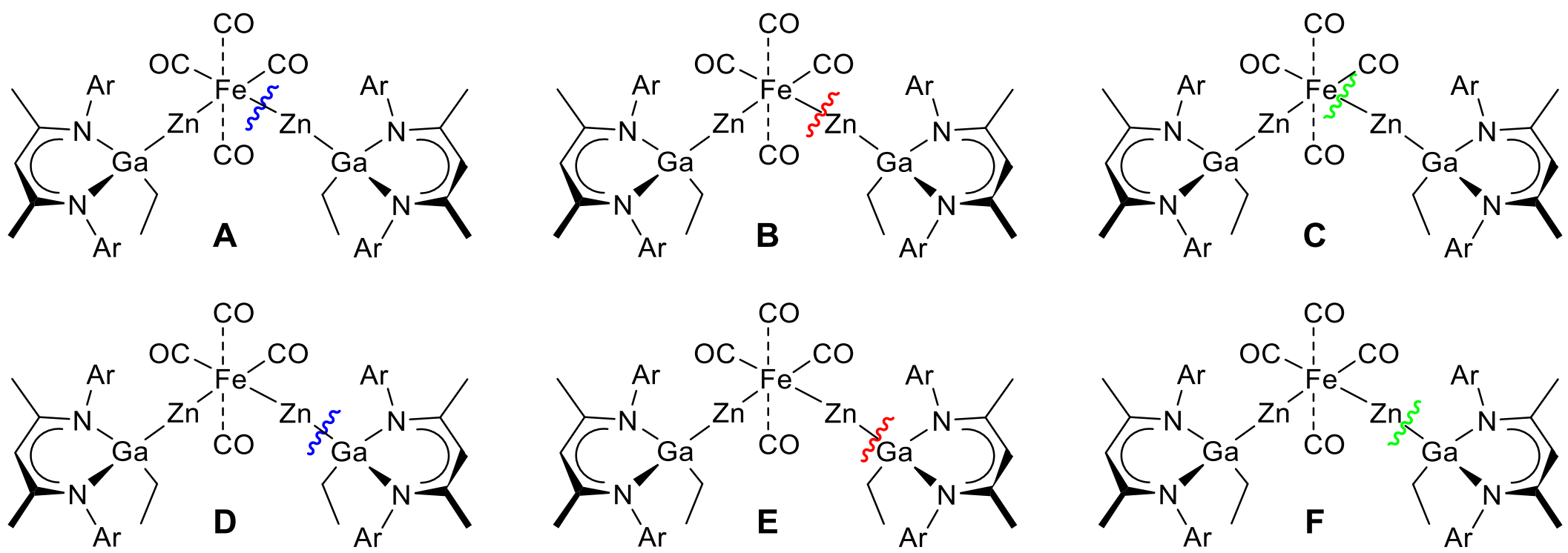

Table S6. Analysis of the total bonding energy of **2** calculated by means of B3LYP-D3BJ/TZ2P. The bond energy is computed as an energy difference between molecule and fragments. The total bonding energy (ΔE) consists of the Pauli repulsion (ΔE_{Pauli}), the electrostatic interaction (ΔV_{elstat}), orbital interactions (ΔE_{oi}) and the dispersion energy (ΔE_{Disp}). The values are given in kcal/mol. Ar = 2,6-*i*$Pr_2C_6H_3$.

Type	Fragments	ΔE_{Pauli}	ΔV_{elstat}	ΔE_{oi}	ΔE_{Disp}	ΔE
**A**	LFe$^{\bullet}$ + $^{\bullet}$ZnL’	180.13	-136.39	-90.91	-24.53	-71.70
**B**	LFe^{-} + $^{+}$ZnL’	156.33	-168.86	-91.99	-24.53	-129.05
**C**	LFe^{+} + $^{-}$ZnL’	306.36	-271.92	-238.18	-24.53	-228.27
**D**	L’’Zn$^{\bullet}$ + $^{\bullet}$GaL’’’	139.36	-120.53	-64.60	-29.99	-75.76
**E**	L’’Zn^{-} + $^{+}$GaL’’’	169.17	-189.06	-113.68	-29.99	-163.56
**F**	L’’Zn^{+} + $^{-}$GaL’’’	196.37	-224.76	-157.48	-29.99	-215.86

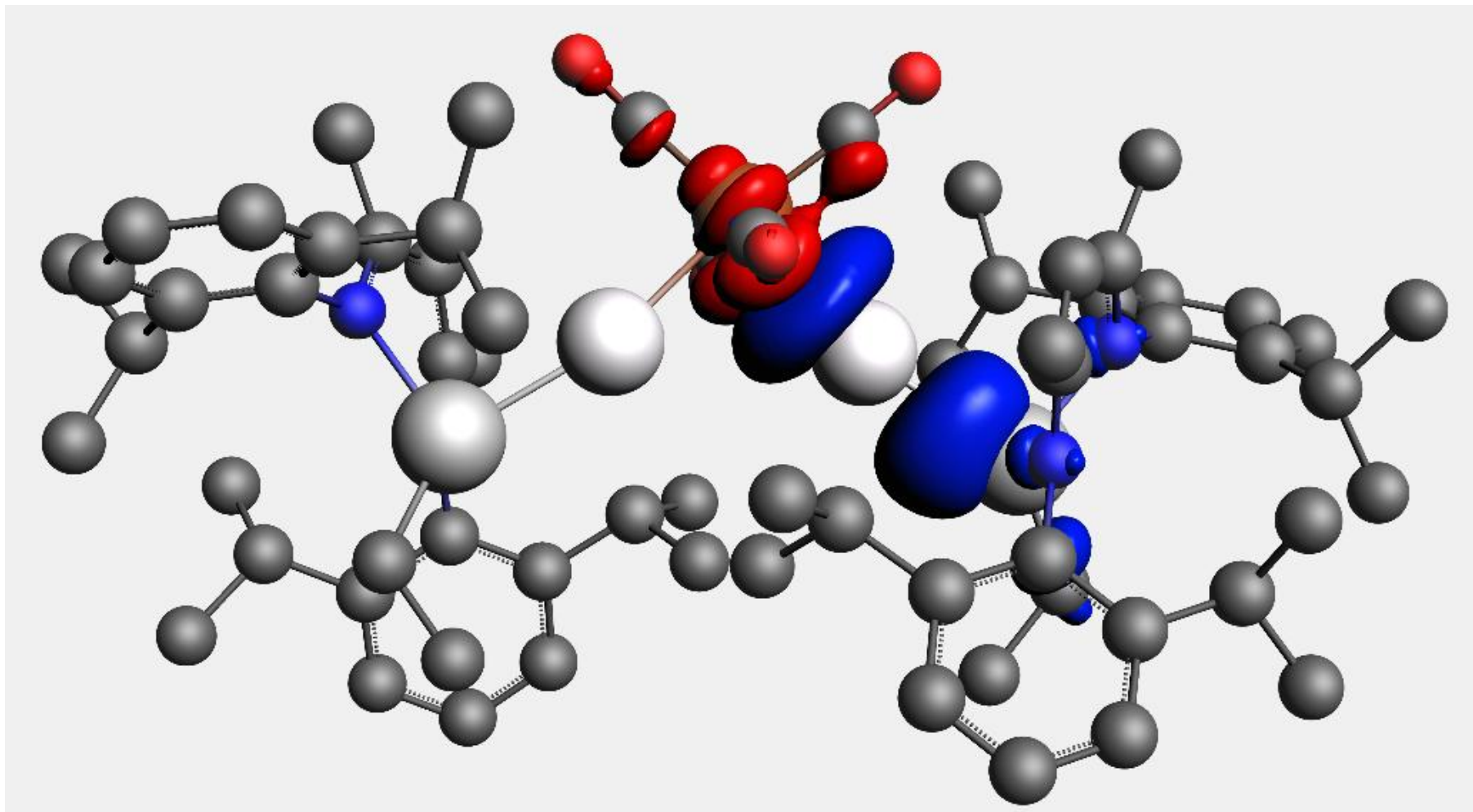

$v_1^{\alpha} = 0.38, \qquad \Delta E_{orb,1}^{\alpha} = -13.32$

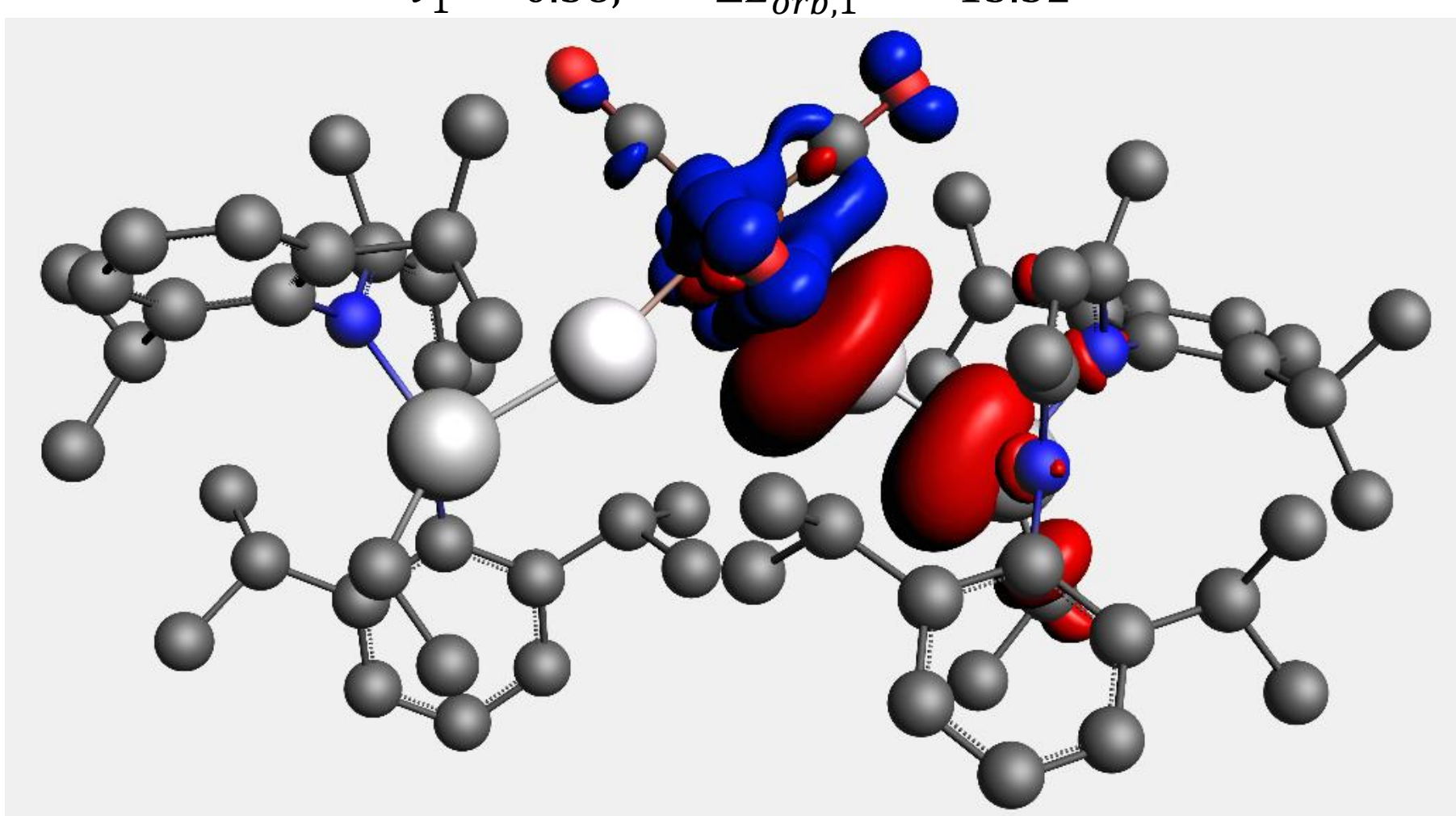

$v_1^{\beta} = 0.73, \qquad \Delta E_{orb,1}^{\beta} = -62.40$

Figure S44. NOCV deformation densities (isovalue = 0.001 au) for the dominant α- and β-spin NOCV pair of the (LFe$^{\bullet}$ + $^{\bullet}$ZnL') system (type **A** in Table S6), calculated at the B3LYP-D3BJ/TZ2P level of theory. The corresponding NOCV eigenvalues (ν) and orbital interaction energy contributions (ΔE_{orb}) are given below each panel. Red and blue isosurfaces indicate electron density depletion and accumulation, respectively. Orbital interaction energy contributions are given in kcal/mol. Hydrogen atoms are omitted for clarity.

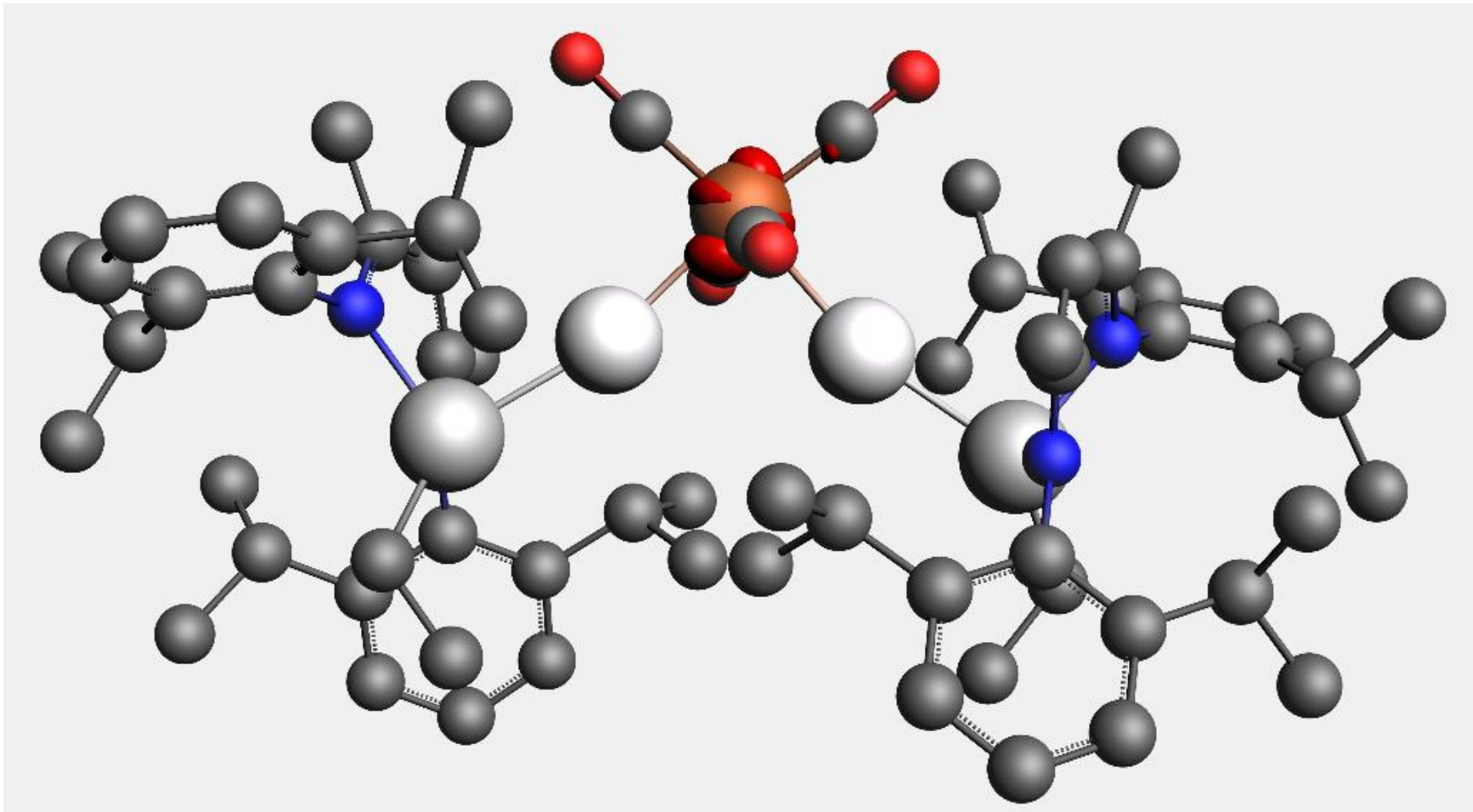

$v_2^{\alpha} = 0.10, \quad \Delta E_{orb,2}^{\alpha} = -1.51$

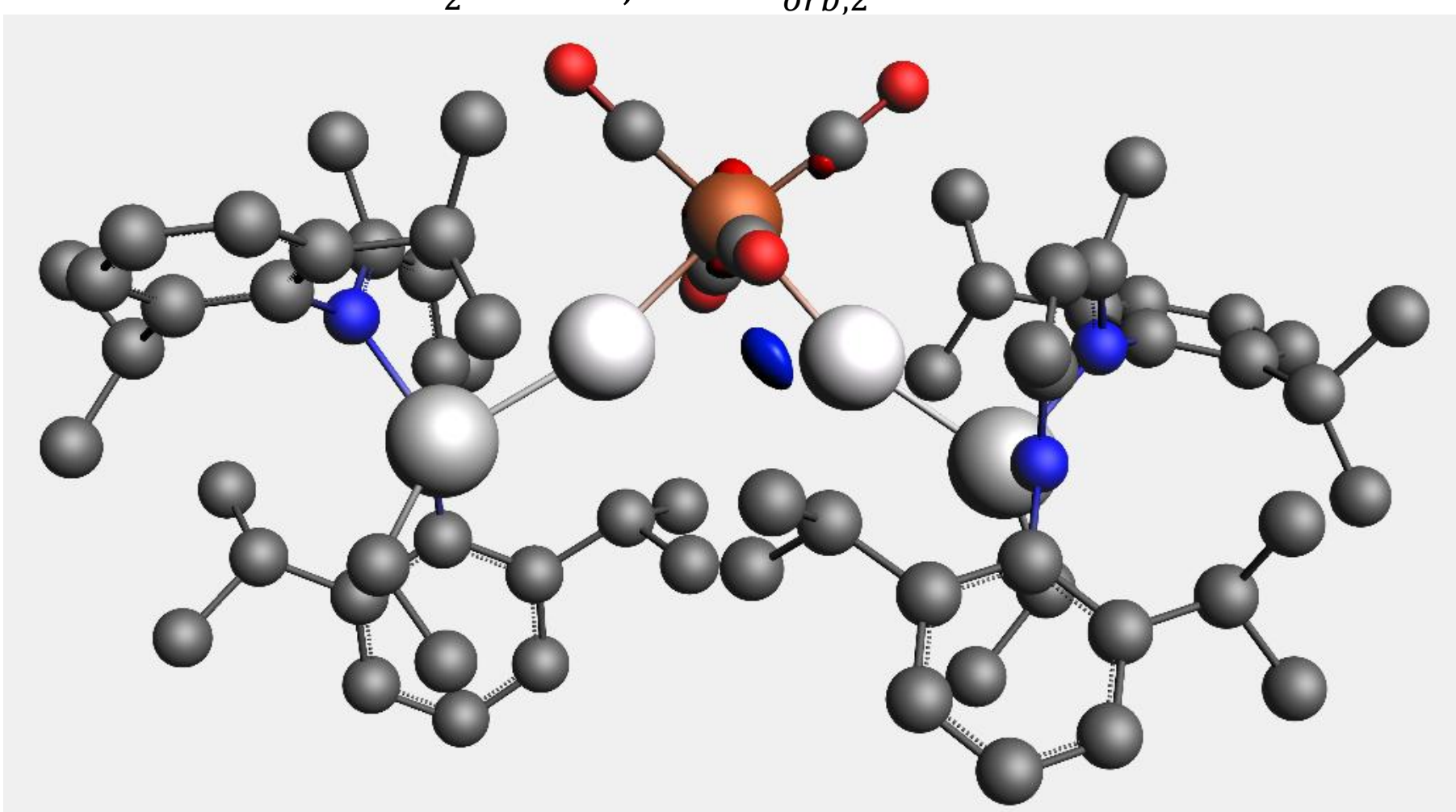

$v_2^{\beta} = 0.09, \quad \Delta E_{orb,2}^{\beta} = -1.74$

Figure S45. NOCV deformation densities (isovalue = 0.001 au) for the second most important α- and β-spin NOCV pair of the (LFe• + •ZnL') system (type **A** in Table S6), calculated at the B3LYP-D3BJ/TZ2P level of theory. The corresponding NOCV eigenvalues (ν) and orbital interaction energy contributions (ΔE_{orb}) are given below each panel. Red and blue isosurfaces indicate electron density depletion and accumulation, respectively. Orbital interaction energy contributions are given in kcal/mol. Hydrogen atoms are omitted for clarity.

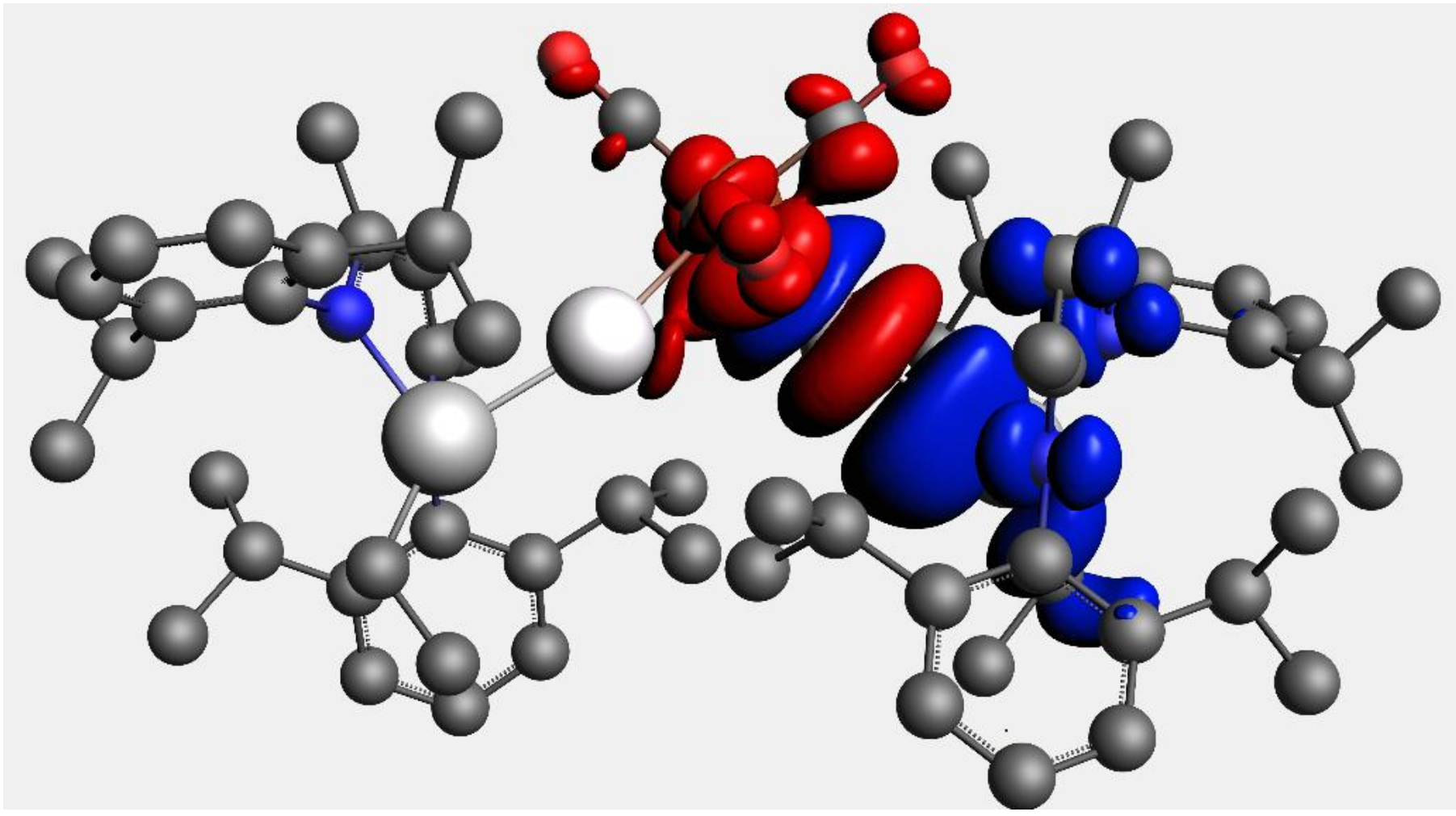

$v_1 = 0.87, \qquad \Delta E_{orb,1} = -58.61$

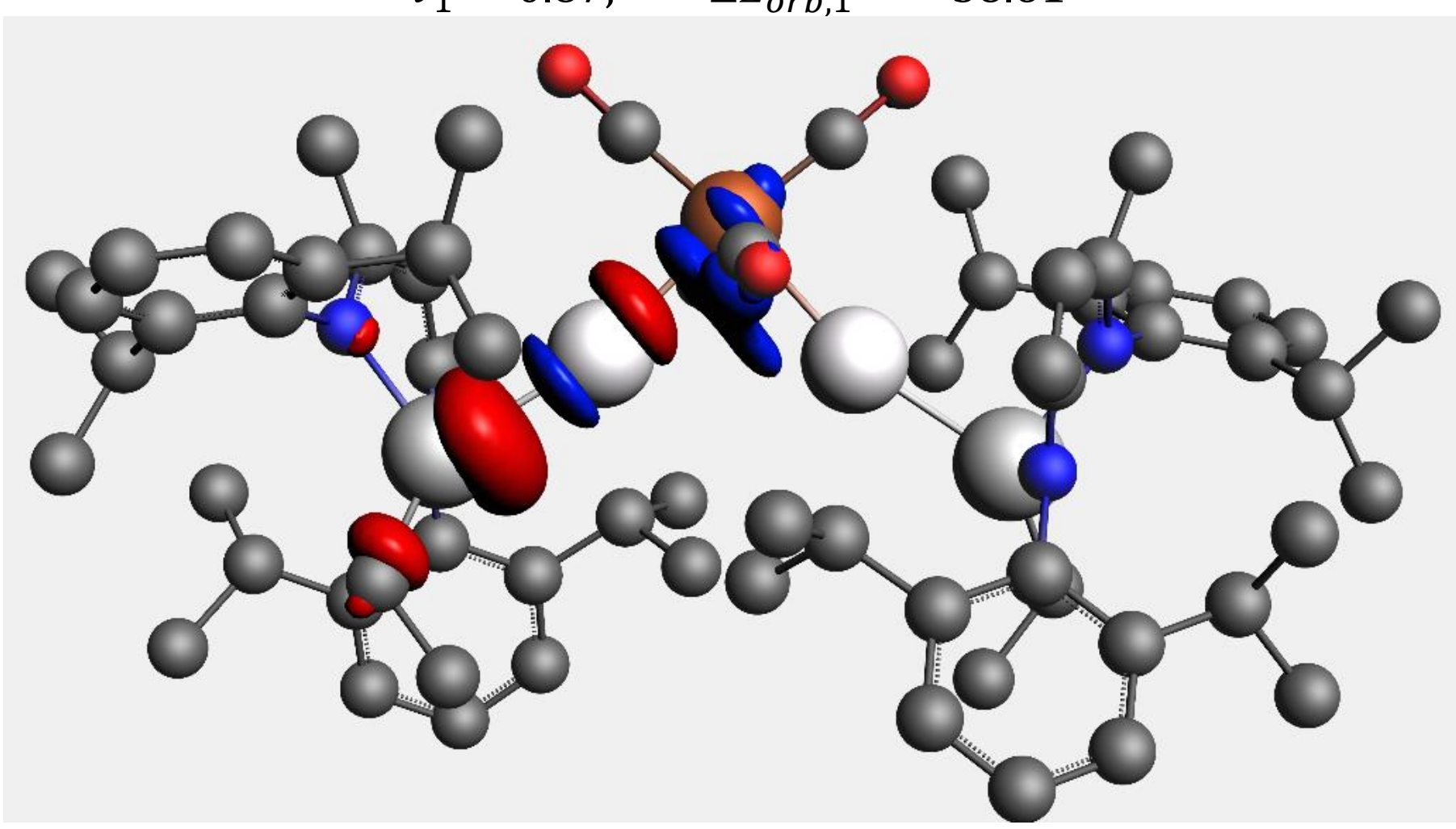

$v_2 = 0.25, \qquad \Delta E_{orb,2} = -4.68$

Figure S46. First two NOCV deformation densities (isovalue = 0.001 au) of the (LFe^{-} + $^{+}ZnL'$) system (type **B** in Table S6), calculated at the B3LYP-D3BJ/TZ2P level of theory. The corresponding NOCV eigenvalues (ν) and orbital interaction energy contributions (ΔE_{orb}) are given below each panel. Red and blue isosurfaces indicate electron density depletion and accumulation, respectively. Orbital interaction energy contributions are given in kcal/mol. Hydrogen atoms are omitted for clarity.

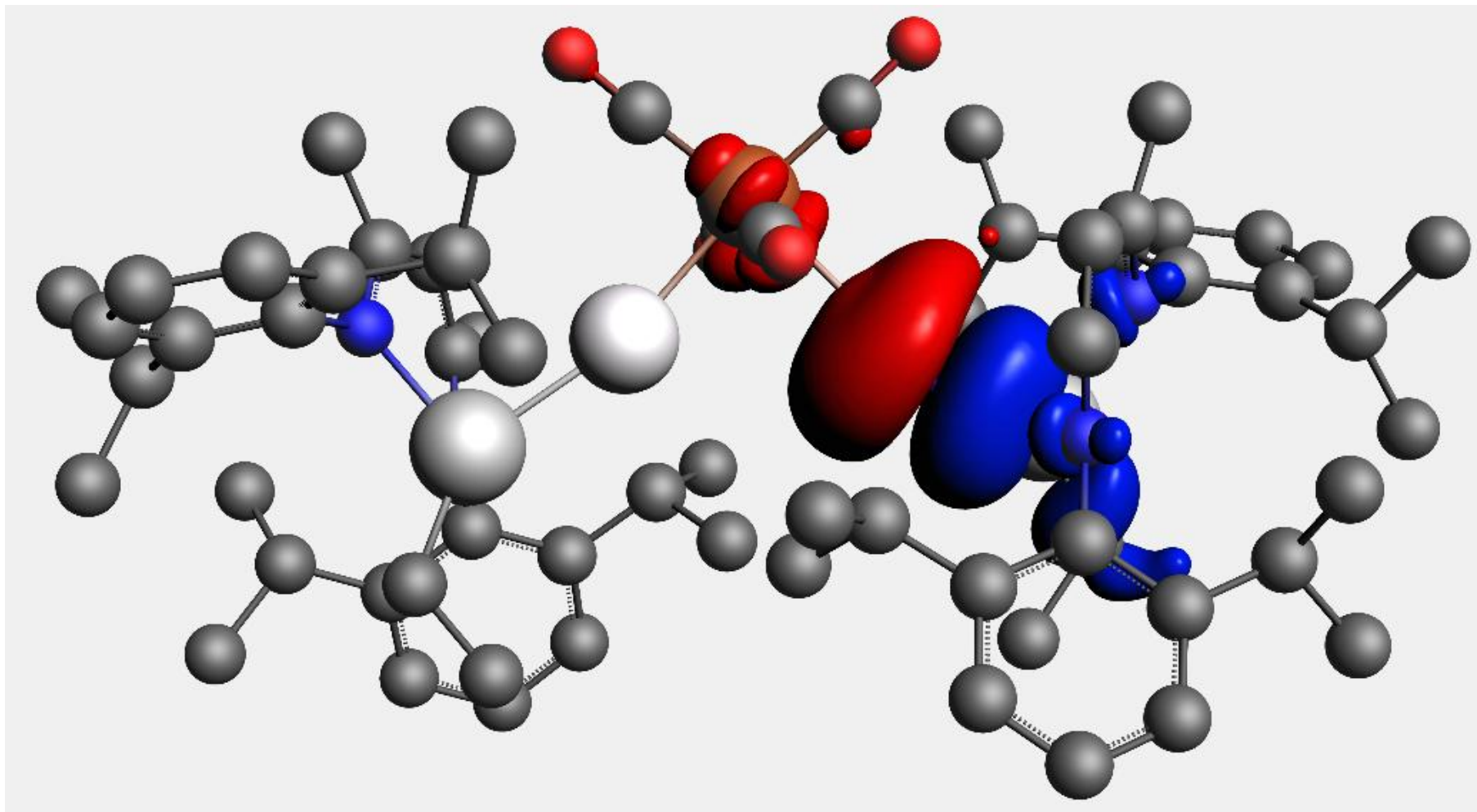

$v_1^{\alpha} = 0.45, \quad \Delta E^{\alpha}_{orb,1} = -24.54$

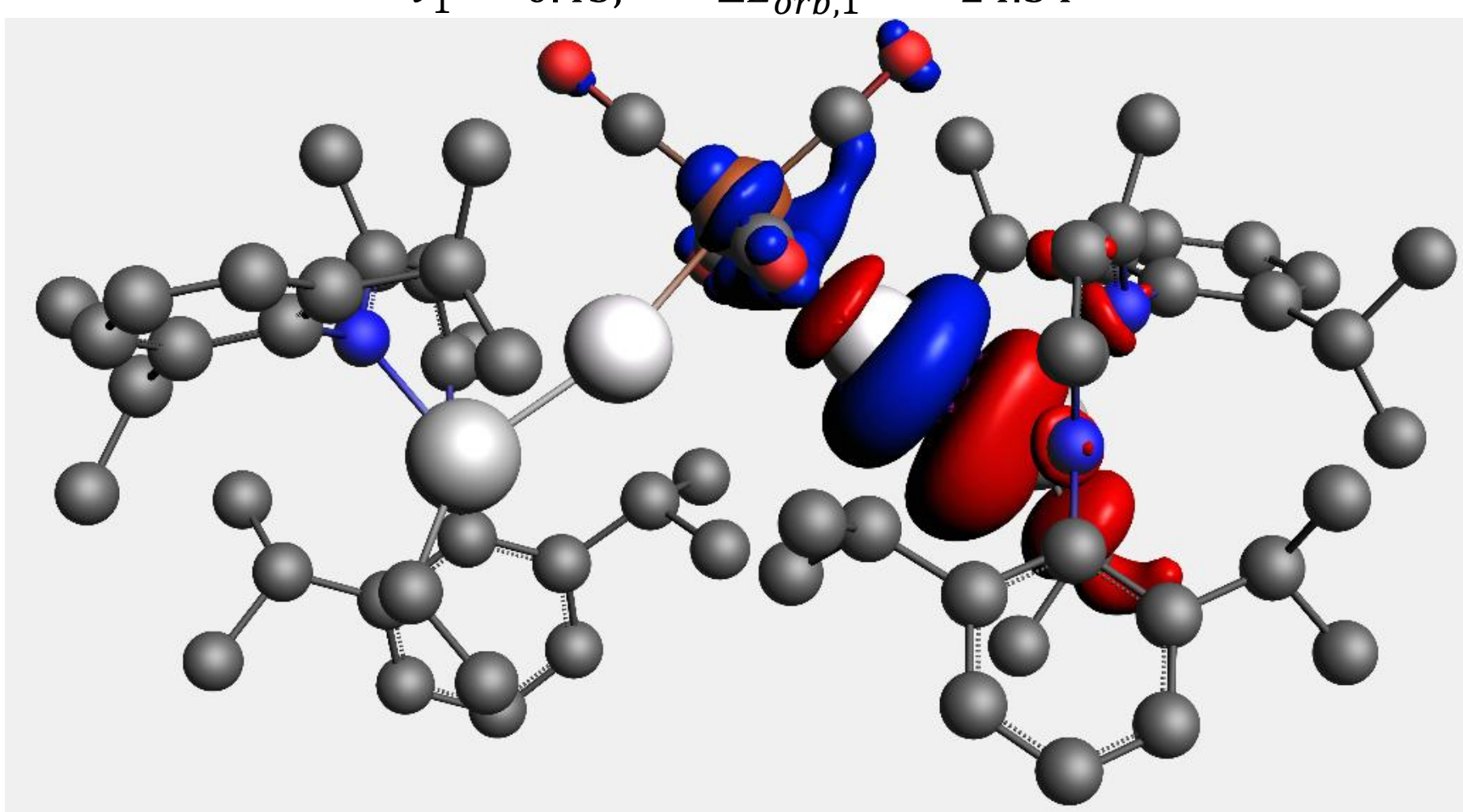

$v_1^{\beta} = 0.50, \quad \Delta E^{\beta}_{orb,1} = -26.58$

Figure S47. NOCV deformation densities (isovalue = 0.001 au) for the dominant α- and β-spin NOCV pair of the (L''Zn• + •GaL''') system (type **D** in Table S6), calculated at the B3LYP-D3BJ/TZ2P level of theory. The corresponding NOCV eigenvalues (ν) and orbital interaction energy contributions (ΔE_{orb}) are given below each panel. Red and blue isosurfaces indicate electron density depletion and accumulation, respectively. Orbital interaction energy contributions are given in kcal/mol. Hydrogen atoms are omitted for clarity.

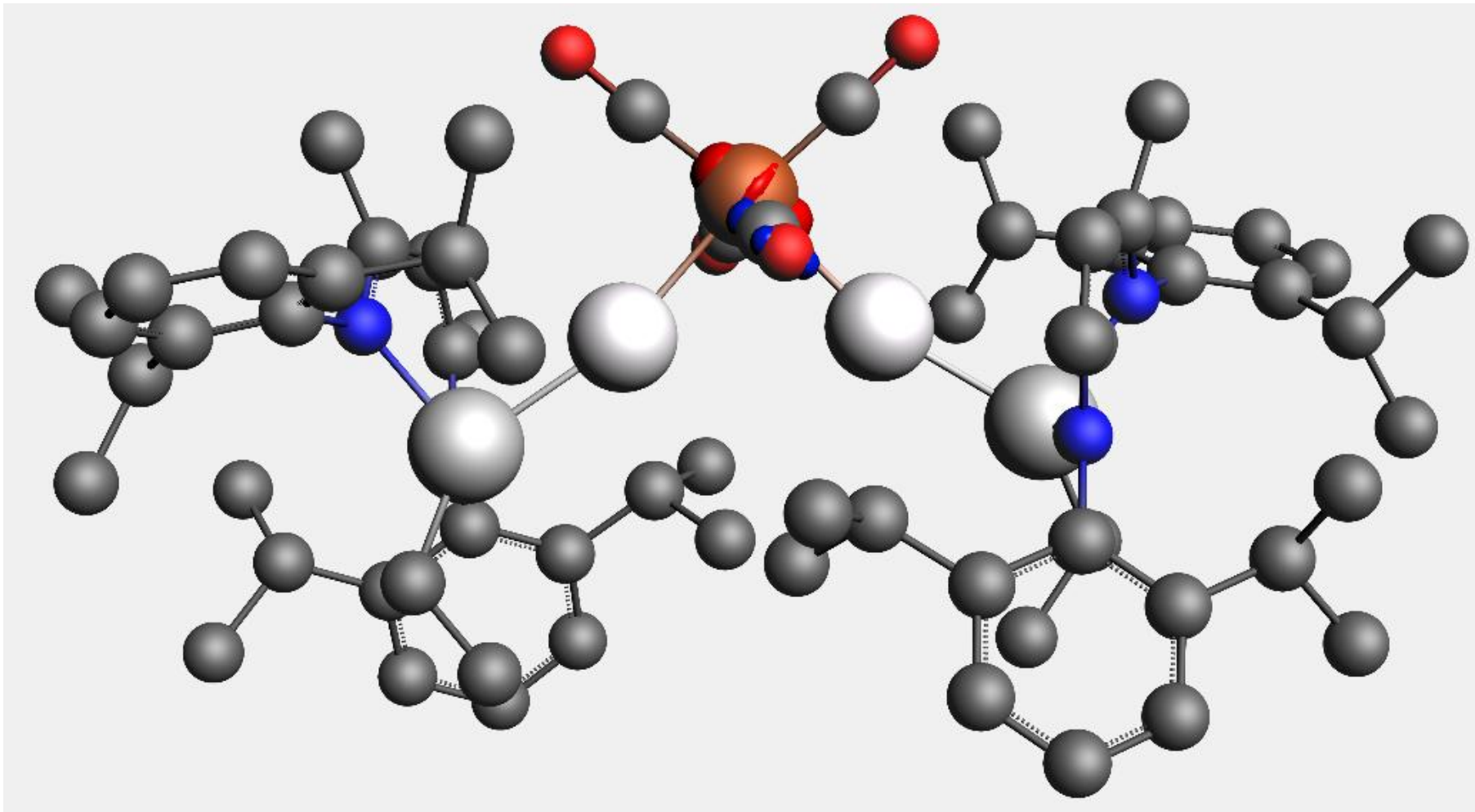

$v_2^{\alpha} = 0.07, \qquad \Delta E_{orb,2}^{\alpha} = -0.76$

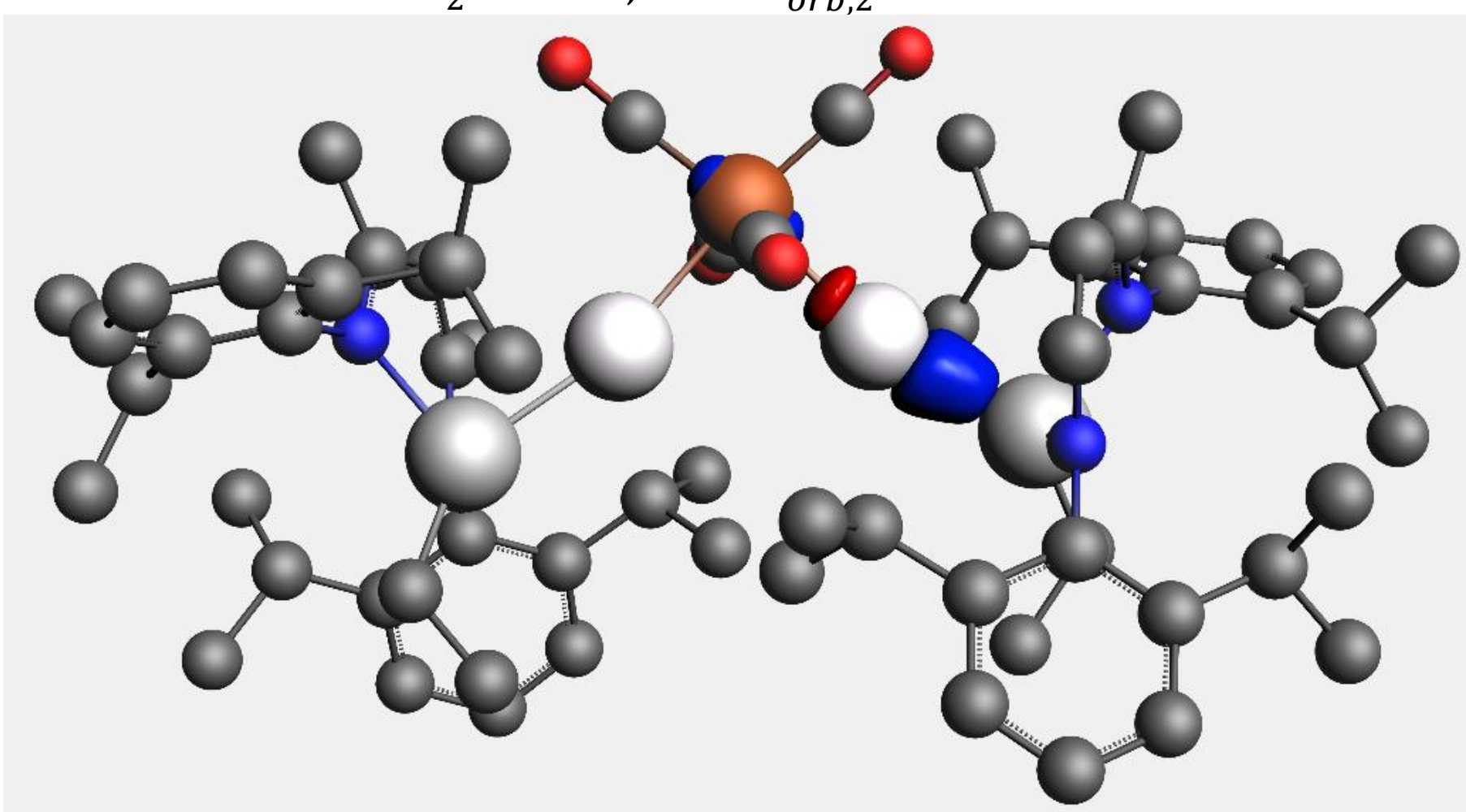

$v_2^{\beta} = 0.08, \qquad \Delta E_{orb,2}^{\beta} = -1.26$

Figure S48. NOCV deformation densities (isovalue = 0.001 au) for the second most important α- and β-spin NOCV pair of the (L''Zn• + •GaL''') system (type **D** in Table S6), calculated at the B3LYP-D3BJ/TZ2P level of theory. The corresponding NOCV eigenvalues (ν) and orbital interaction energy contributions (ΔE_{orb}) are given below each panel. Red and blue isosurfaces indicate electron density depletion and accumulation, respectively. Orbital interaction energy contributions are given in kcal/mol. Hydrogen atoms are omitted for clarity.

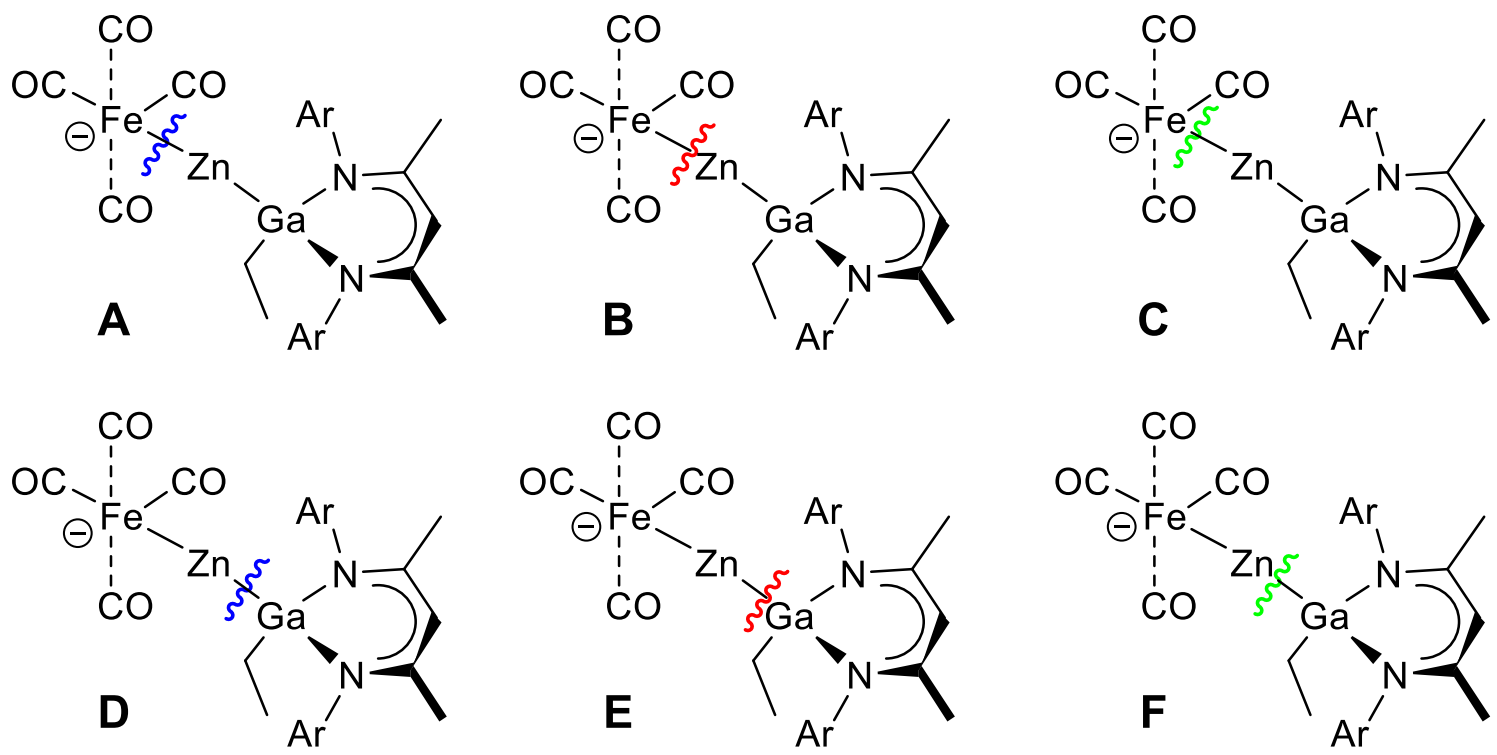

Table S7. Analysis of the total bonding energy of the anionic part of **3** calculated by means of B3LYP-D3BJ/TZ2P. The bond energy is computed as an energy difference between molecule and fragments. The total bonding energy (ΔE) consists of the Pauli repulsion ($\Delta E_{\mathrm{Pauli}}$), the electrostatic interaction ($\Delta V_{\mathrm{elstat}}$), orbital interactions (ΔE_{oi}) and the dispersion energy (ΔE_{Disp}). The values are given in kcal/mol. Ar = 2,6-*i*$Pr_2C_6H_3$.

Type	Fragments	$\Delta E_{\mathrm{Pauli}}$	$\Delta V_{\mathrm{elstat}}$	ΔE_{oi}	ΔE_{Disp}	ΔE
**A**	LFe$^{-\bullet}$ + $^{\bullet}$ZnL’	194.22	-150.75	-95.70	-15.33	-67.56
**B**	LFe^{2-} + $^{+}$ZnL’	185.88	-278.96	-144.80	-15.33	-253.22
**C**	LFe + $^{-}$ZnL’	262.58	-204.17	-174.61	-15.33	-131.54
**D**	L’’Zn$^{-\bullet}$ + $^{\bullet}$GaL’’’	147.41	-129.64	-66.30	-20.16	-68.69
**E**	L’’Zn^{2-} + $^{+}$GaL’’’	213.18	-297.14	-158.95	-20.16	-263.07
**F**	L’’Zn + $^{-}$GaL’’’	178.22	-164.12	-107.87	-20.16	-113.93

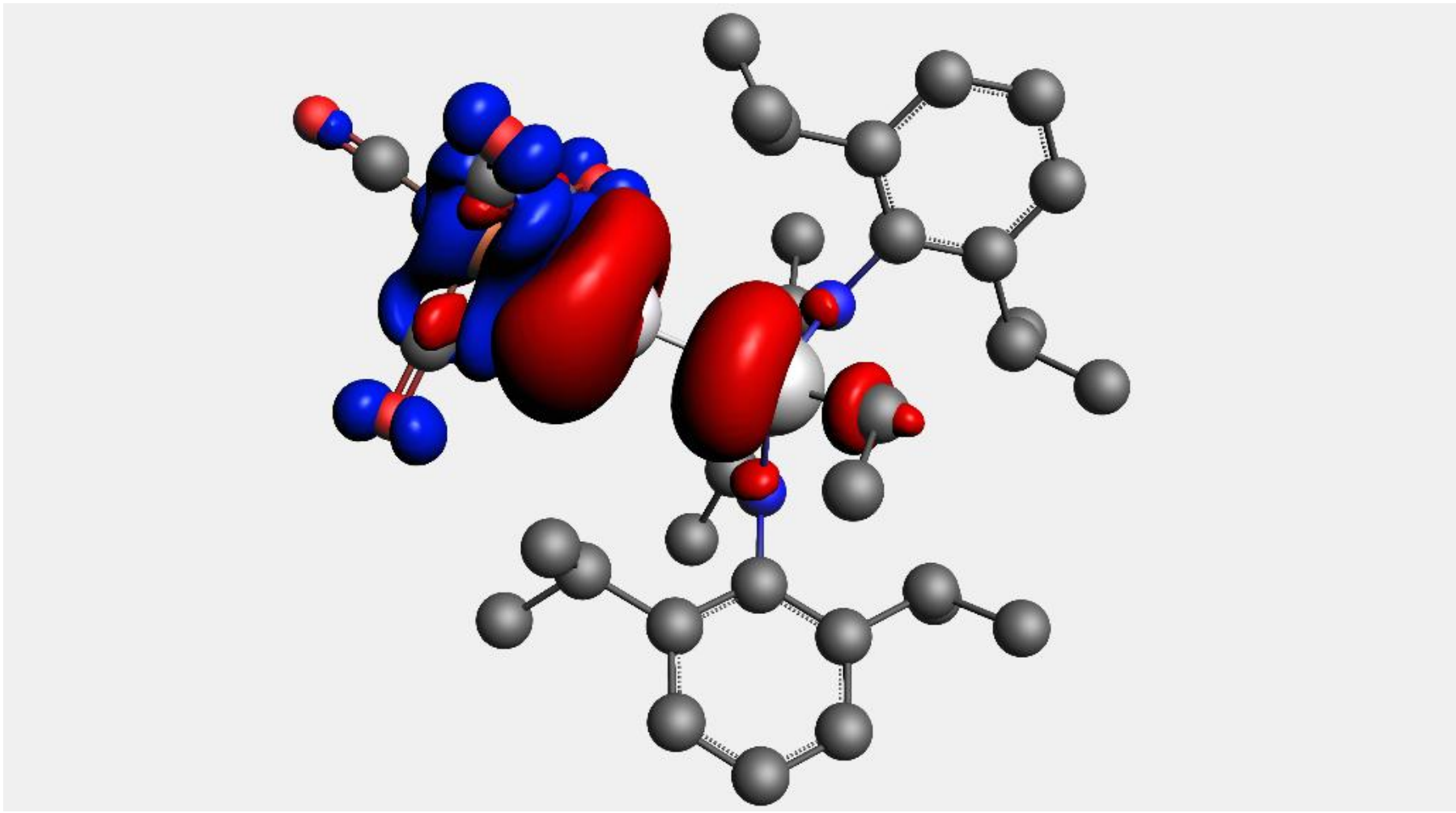

$v_1^{\alpha} = 0.67, \qquad \Delta E_{orb,1}^{\alpha} = -52.26$

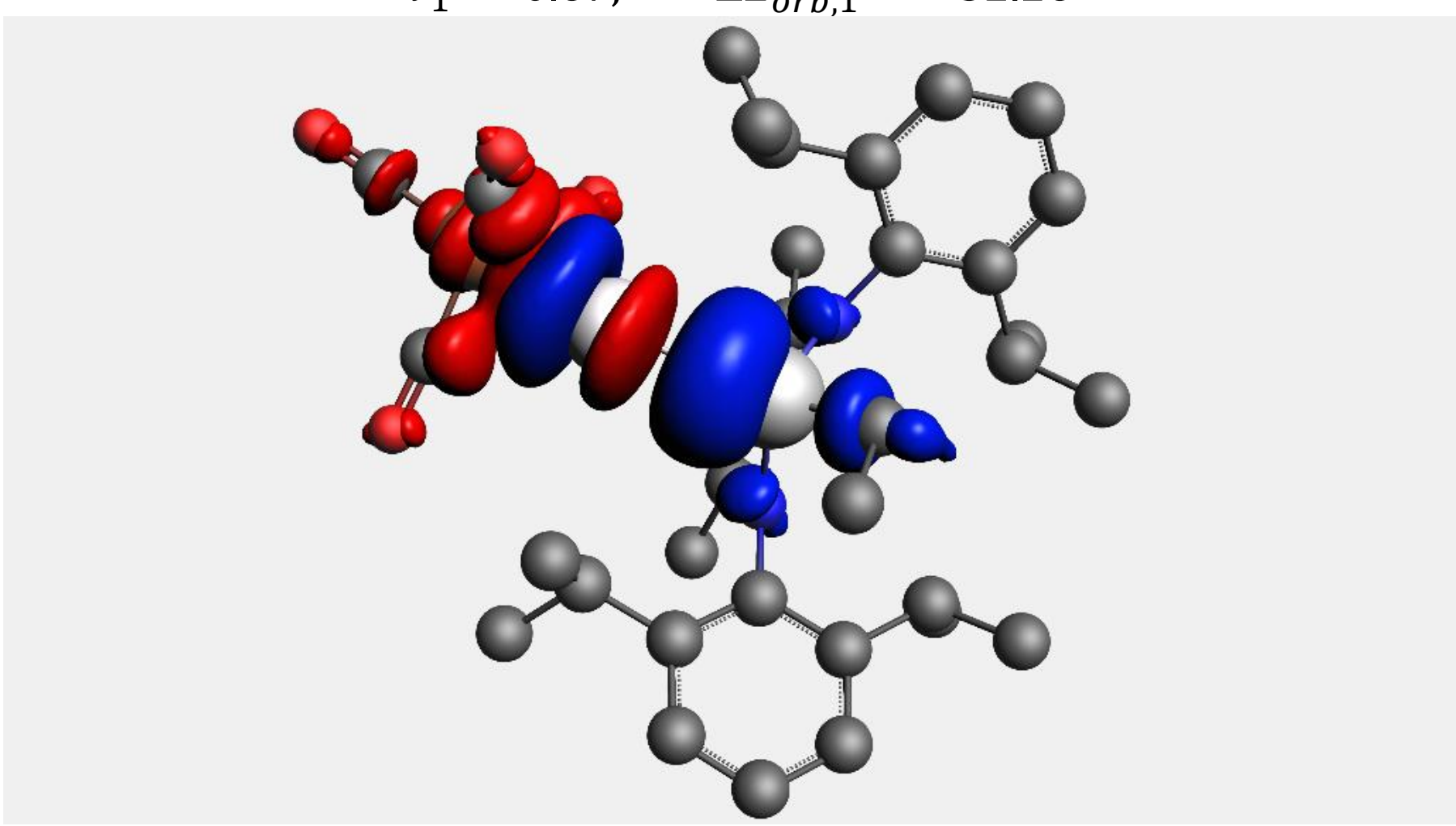

$v_1^{\beta} = 0.45, \qquad \Delta E_{orb,1}^{\beta} = -22.69$

Figure S49. NOCV deformation densities (isovalue = 0.001 au) for the dominant α- and β-spin NOCV pair of the ($LFe^{-\bullet}$ + $^{\bullet}$ZnL') system (type **A** in Table S7), calculated at the B3LYP-D3BJ/TZ2P level of theory. The corresponding NOCV eigenvalues (ν) and orbital interaction energy contributions (ΔE_{orb}) are given below each panel. Red and blue isosurfaces indicate electron density depletion and accumulation, respectively. Orbital interaction energy contributions are given in kcal/mol. Hydrogen atoms are omitted for clarity.

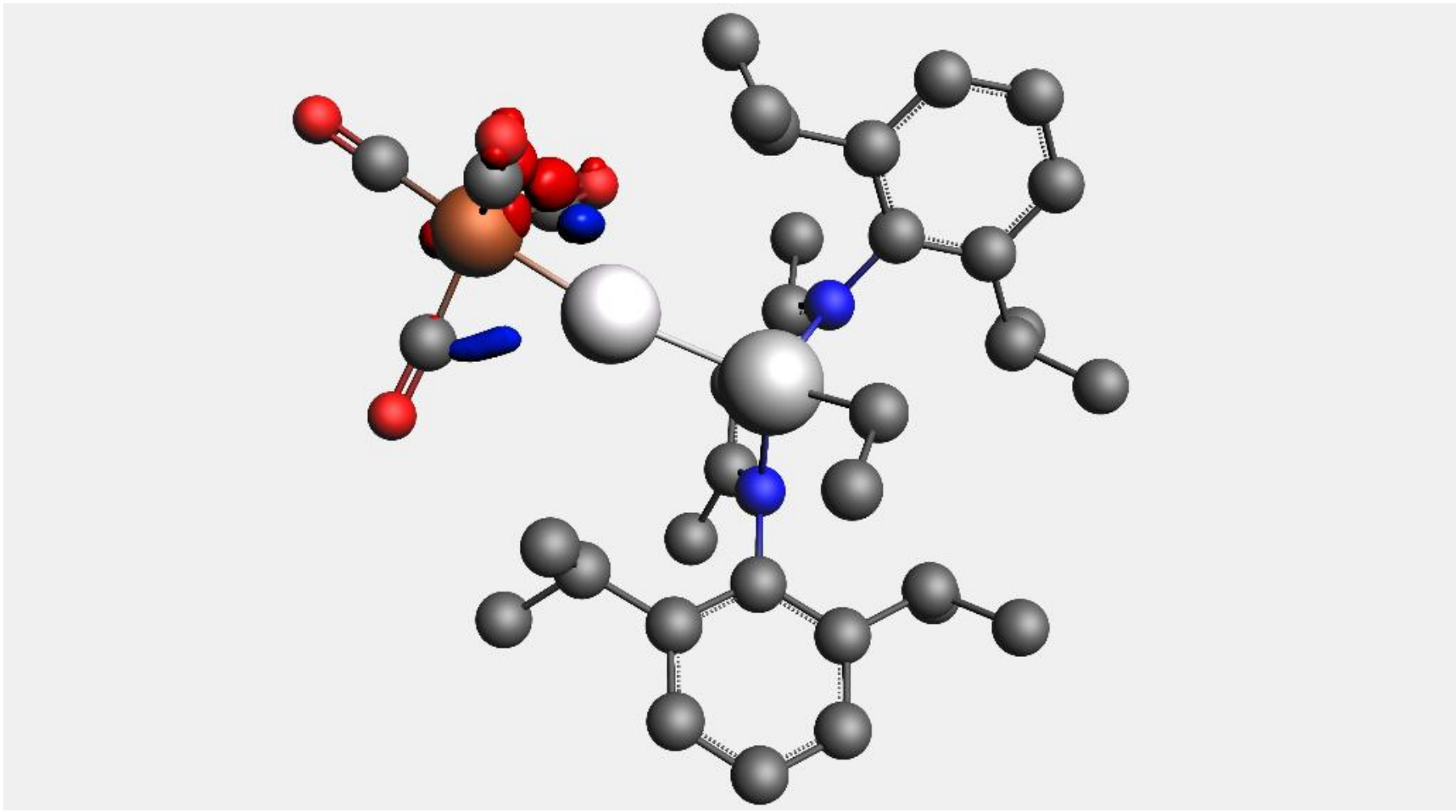

$v_2^\alpha = 0.12, \qquad \Delta E_{orb,2}^\alpha = -2.52$

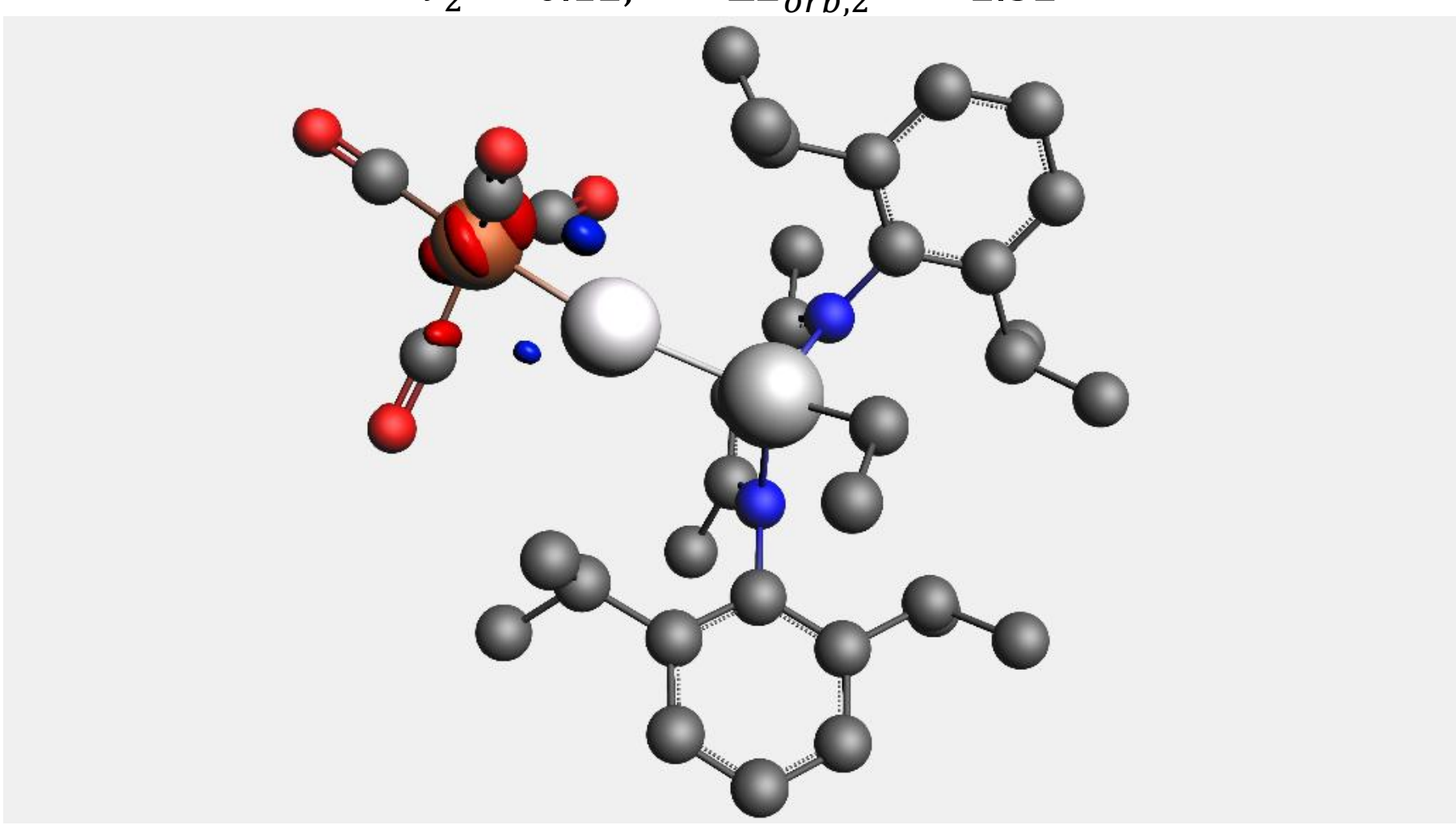

$v_2^\beta = 0.12, \qquad \Delta E_{orb,2}^\beta = -2.24$

Figure S50. NOCV deformation densities (isovalue = 0.001 au) for the second most important α- and β-spin NOCV pair of the ($LFe^{-\bullet}$ + $^{\bullet}ZnL'$) system (type **A** in Table S7), calculated at the B3LYP-D3BJ/TZ2P level of theory. The corresponding NOCV eigenvalues (ν) and orbital interaction energy contributions (ΔE_{orb}) are given below each panel. Red and blue isosurfaces indicate electron density depletion and accumulation, respectively. Orbital interaction energy contributions are given in kcal/mol. Hydrogen atoms are omitted for clarity.

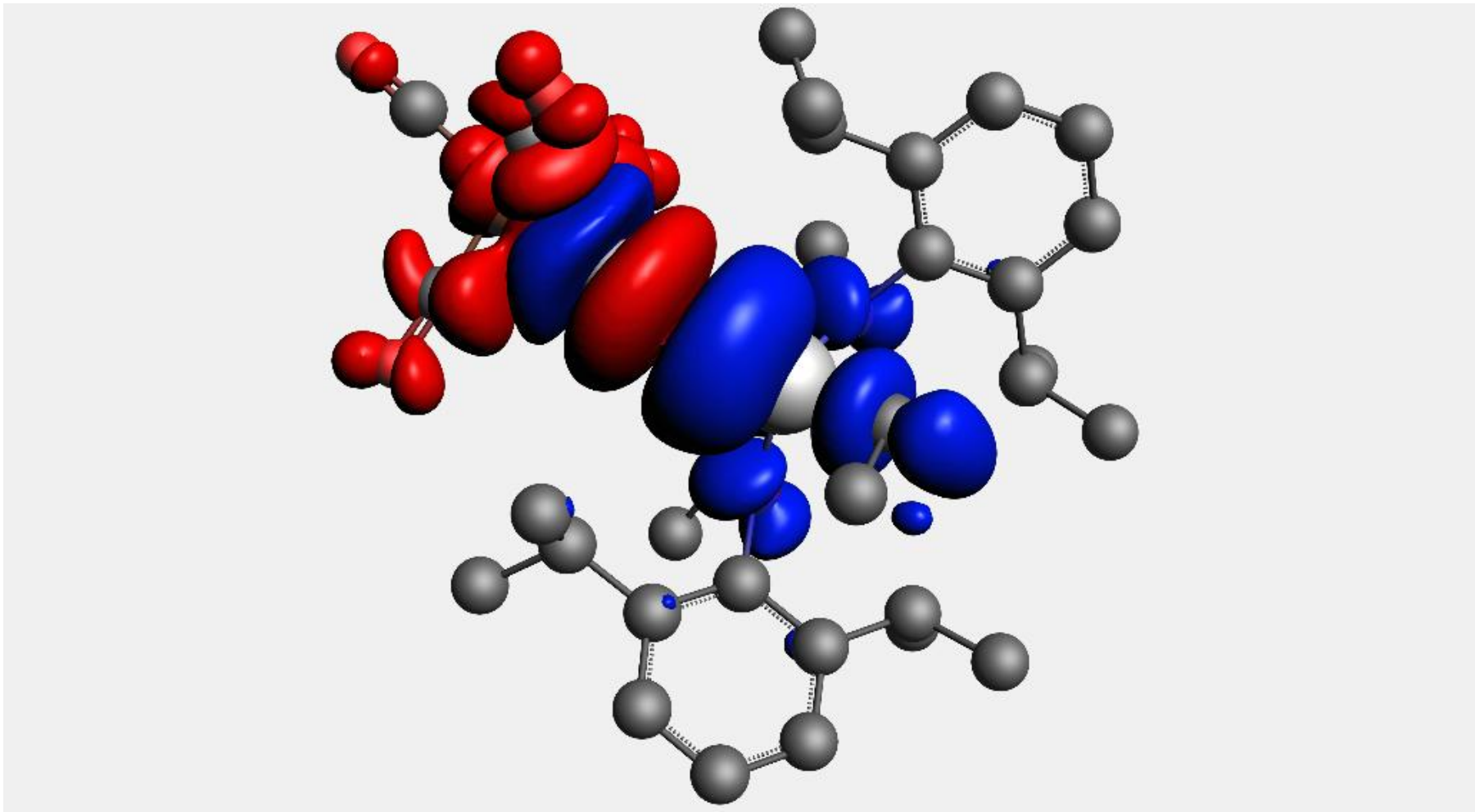

$v_1 = 1.03, \quad \Delta E_{orb,1} = -94.25$

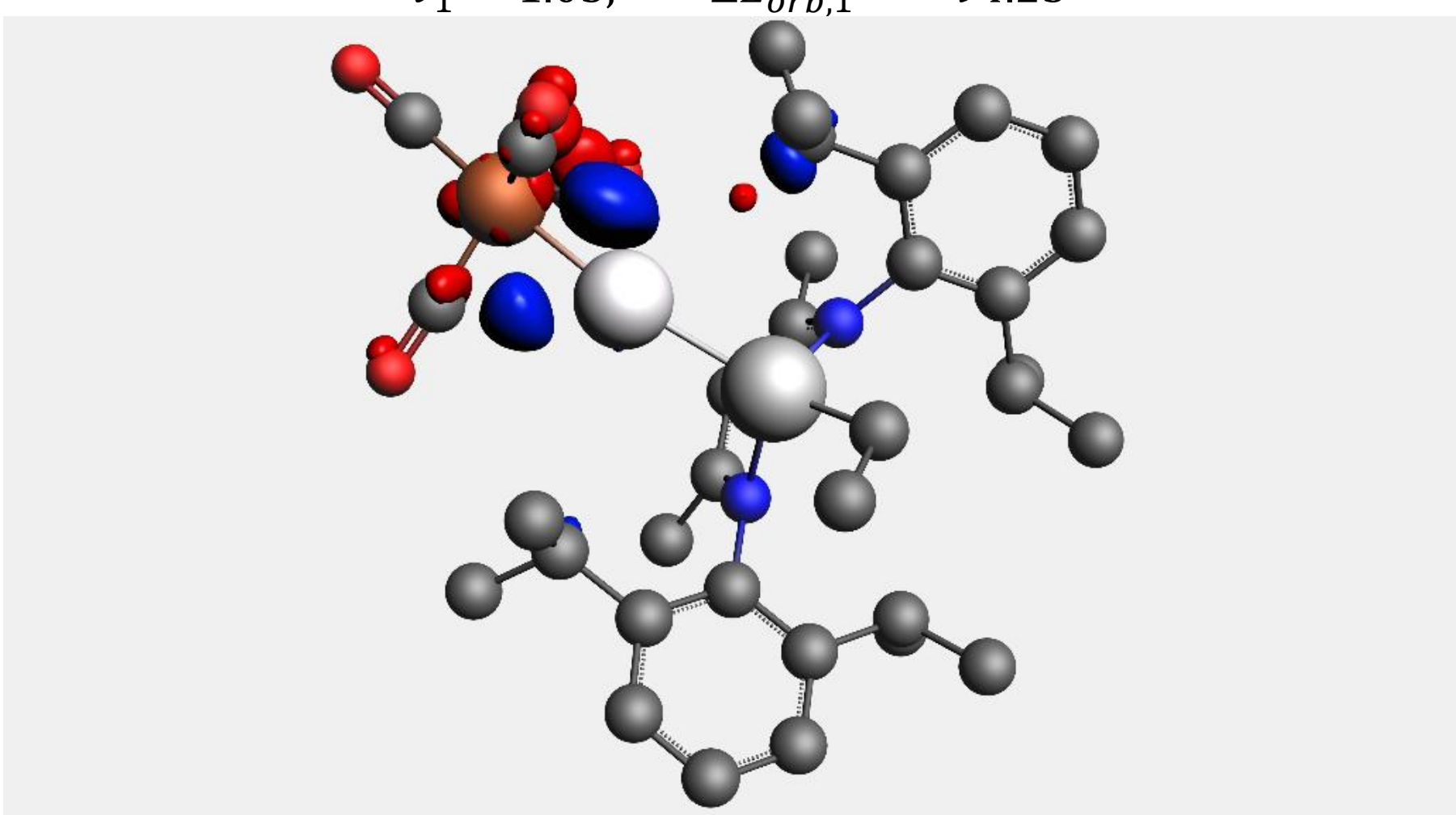

$v_2 = 0.24, \quad \Delta E_{orb,2} = -7.38$

Figure S51. First two NOCV deformation densities (isovalue = 0.001 au) of the (LFe^{2-} + $^{+}ZnL'$) system (type **B** in Table S7), calculated at the B3LYP-D3BJ/TZ2P level of theory. The corresponding NOCV eigenvalues (ν) and orbital interaction energy contributions (ΔE_{orb}) are given below each panel. Red and blue isosurfaces indicate electron density depletion and accumulation, respectively. Orbital interaction energy contributions are given in kcal/mol. Hydrogen atoms are omitted for clarity.

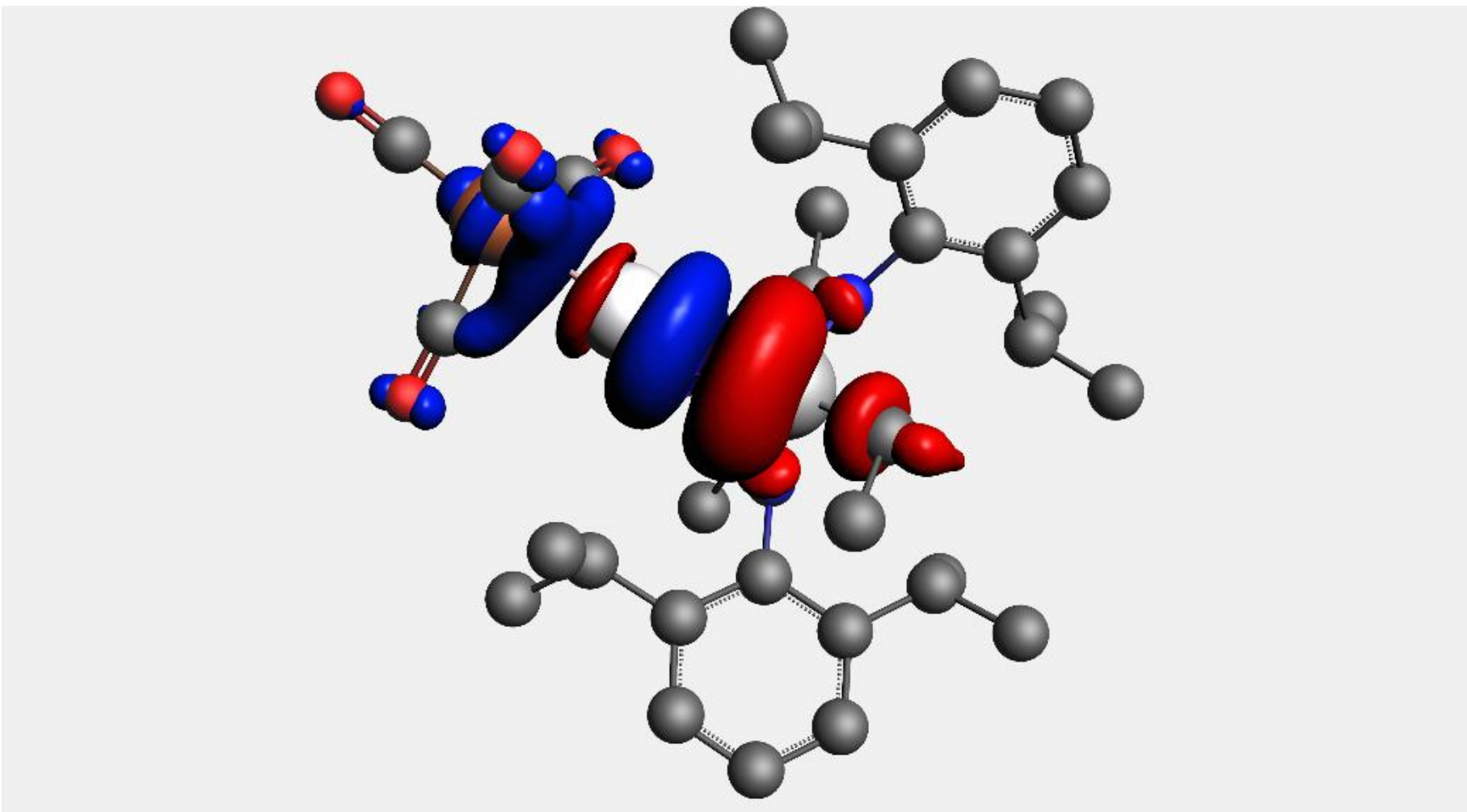

$v_1^{\alpha} = 0.42, \quad \Delta E_{orb,1}^{\alpha} = -18.15$

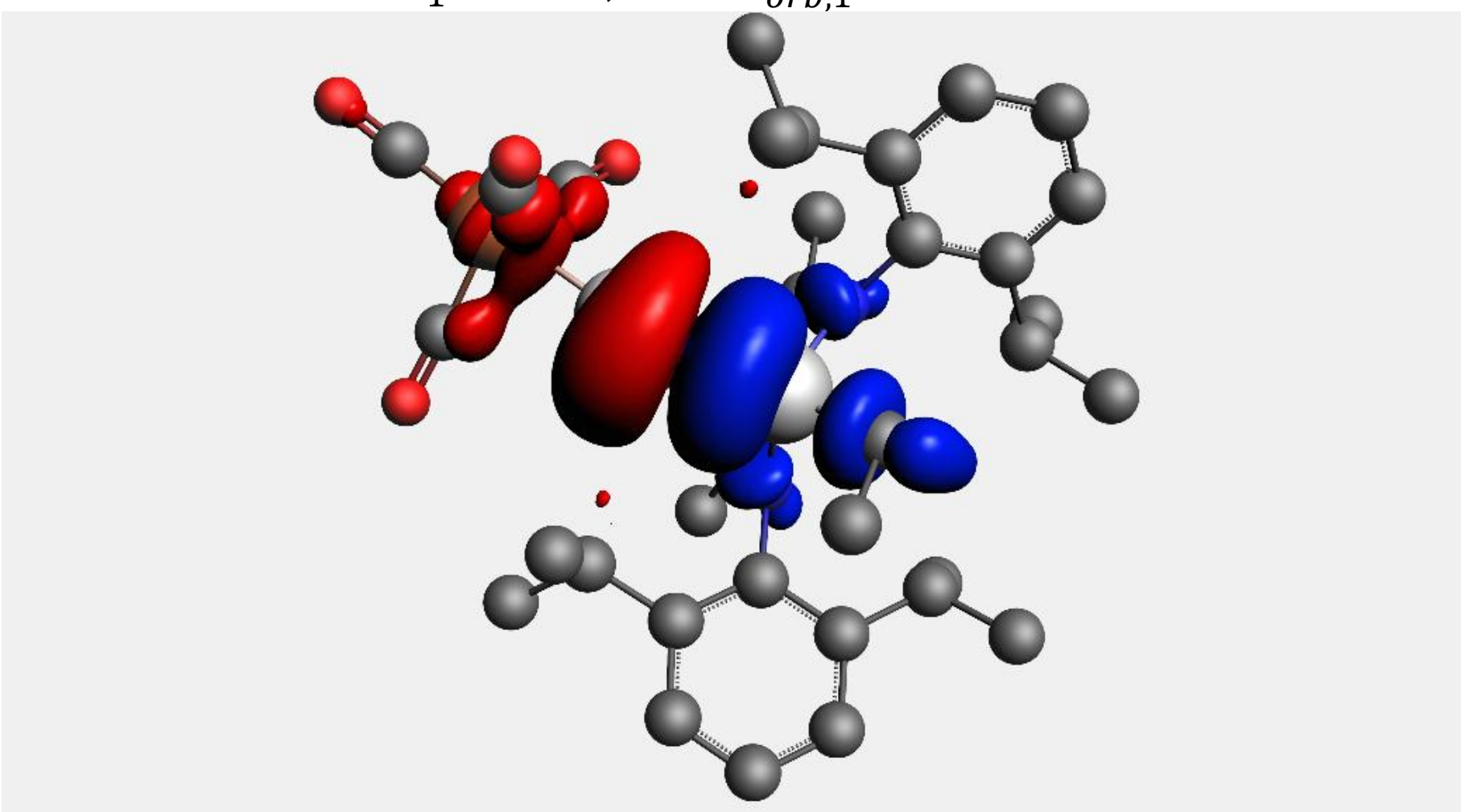

$v_1^{\beta} = 0.51, \quad \Delta E_{orb,1}^{\beta} = -34.72$

Figure S52. NOCV deformation densities (isovalue = 0.001 au) for the dominant α- and β-spin NOCV pair of the (L’’Zn$^{-\bullet}$ + $^{\bullet}$GaL’’’) system (type **D** in Table S7), calculated at the B3LYP-D3BJ/TZ2P level of theory. The corresponding NOCV eigenvalues (ν) and orbital interaction energy contributions (ΔE_{orb}) are given below each panel. Red and blue isosurfaces indicate electron density depletion and accumulation, respectively. Orbital interaction energy contributions are given in kcal/mol. Hydrogen atoms are omitted for clarity.

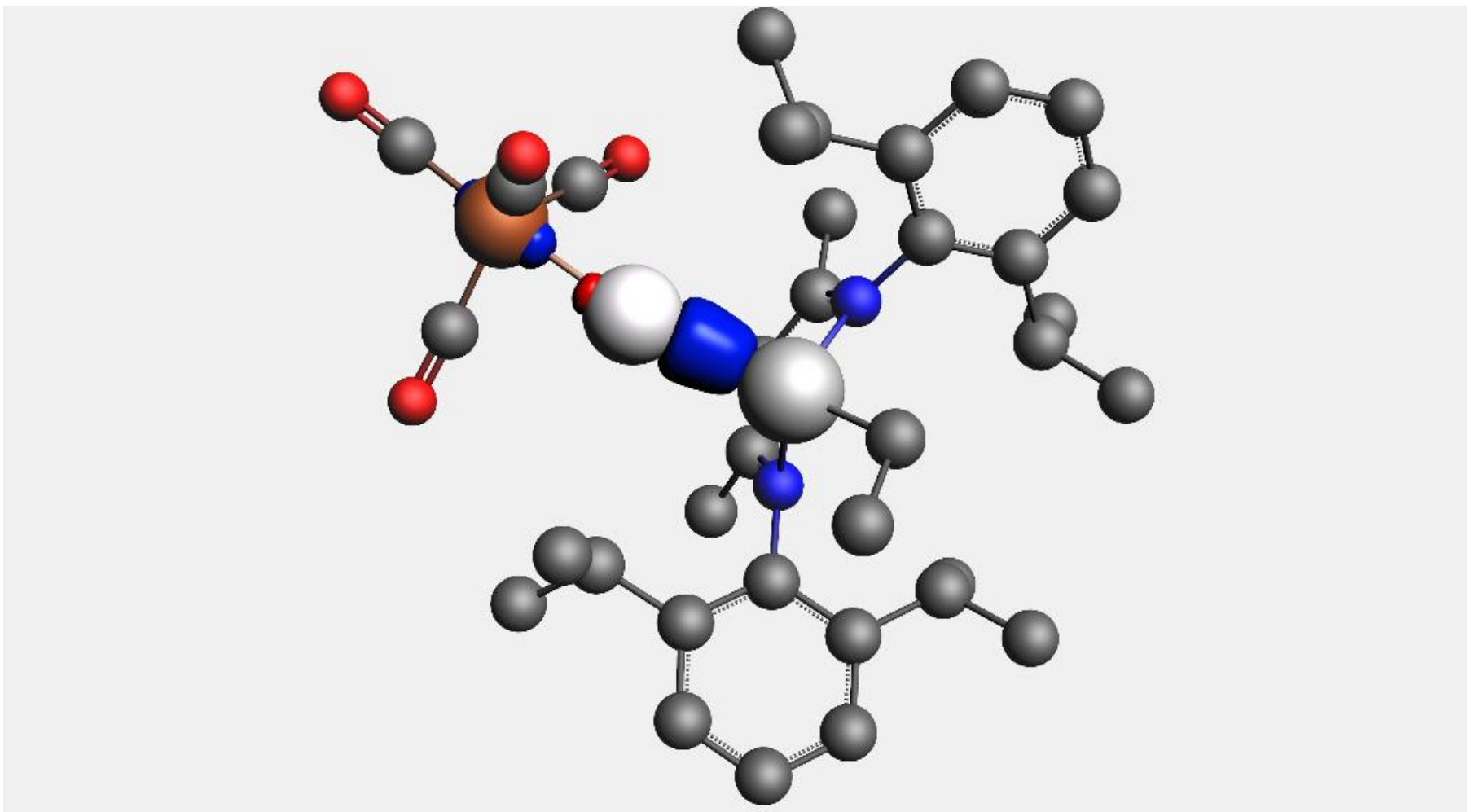

$v_2^{\alpha} = 0.08, \qquad \Delta E^{\alpha}_{orb,1} = -1.54$

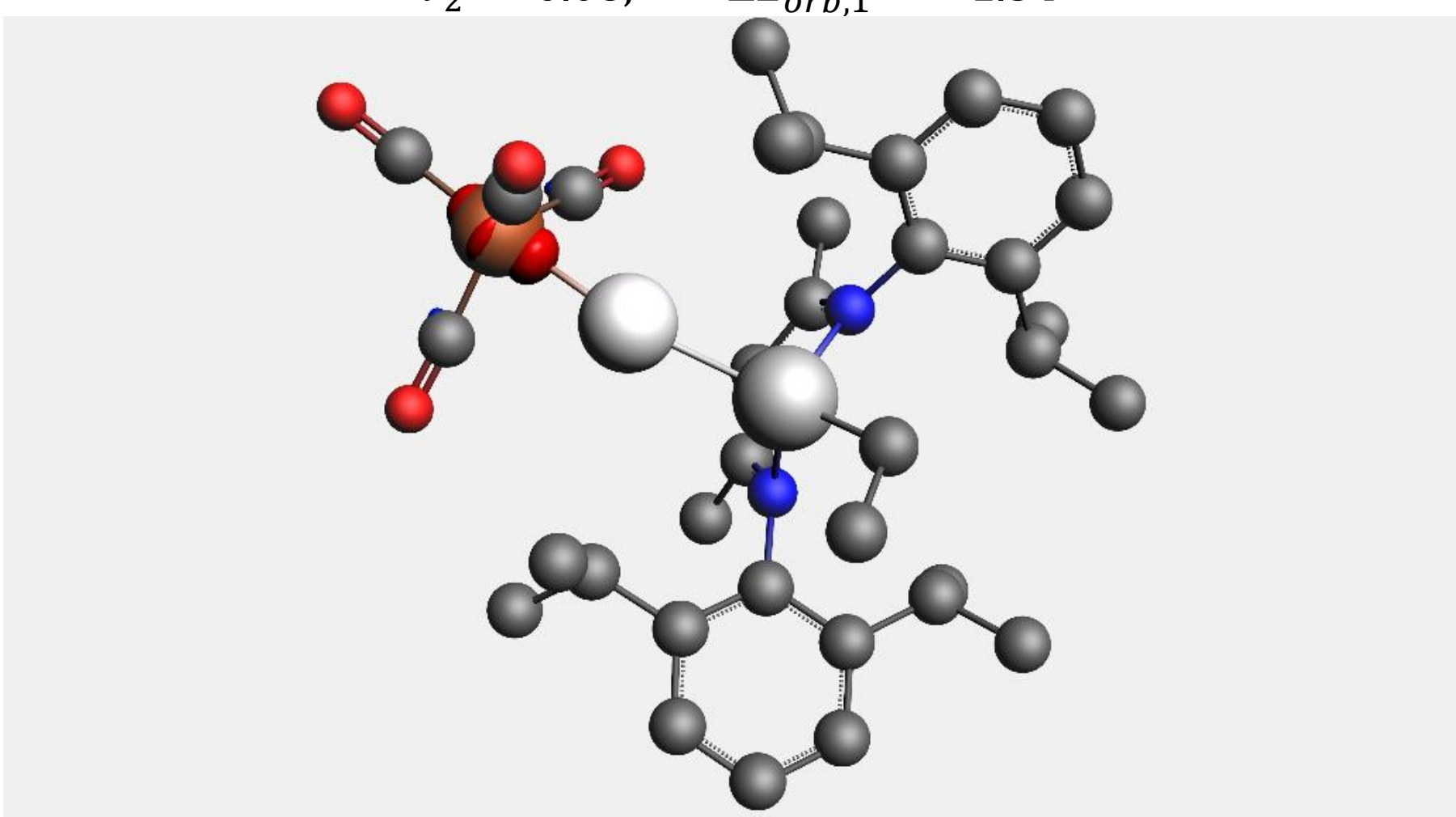

$v_2^{\beta} = 0.07, \qquad \Delta E^{\beta}_{orb,1} = -0.88$

Figure S53. NOCV deformation densities (isovalue = 0.001 au) for the second most important α- and β-spin NOCV pair of the (L’’Zn$^{-\bullet}$ + $^{\bullet}$GaL’’’) system (type **D** in Table S7), calculated at the B3LYP-D3BJ/TZ2P level of theory. The corresponding NOCV eigenvalues (ν) and orbital interaction energy contributions (ΔE_{orb}) are given below each panel. Red and blue isosurfaces indicate electron density depletion and accumulation, respectively. Orbital interaction energy contributions are given in kcal/mol. Hydrogen atoms are omitted for clarity.

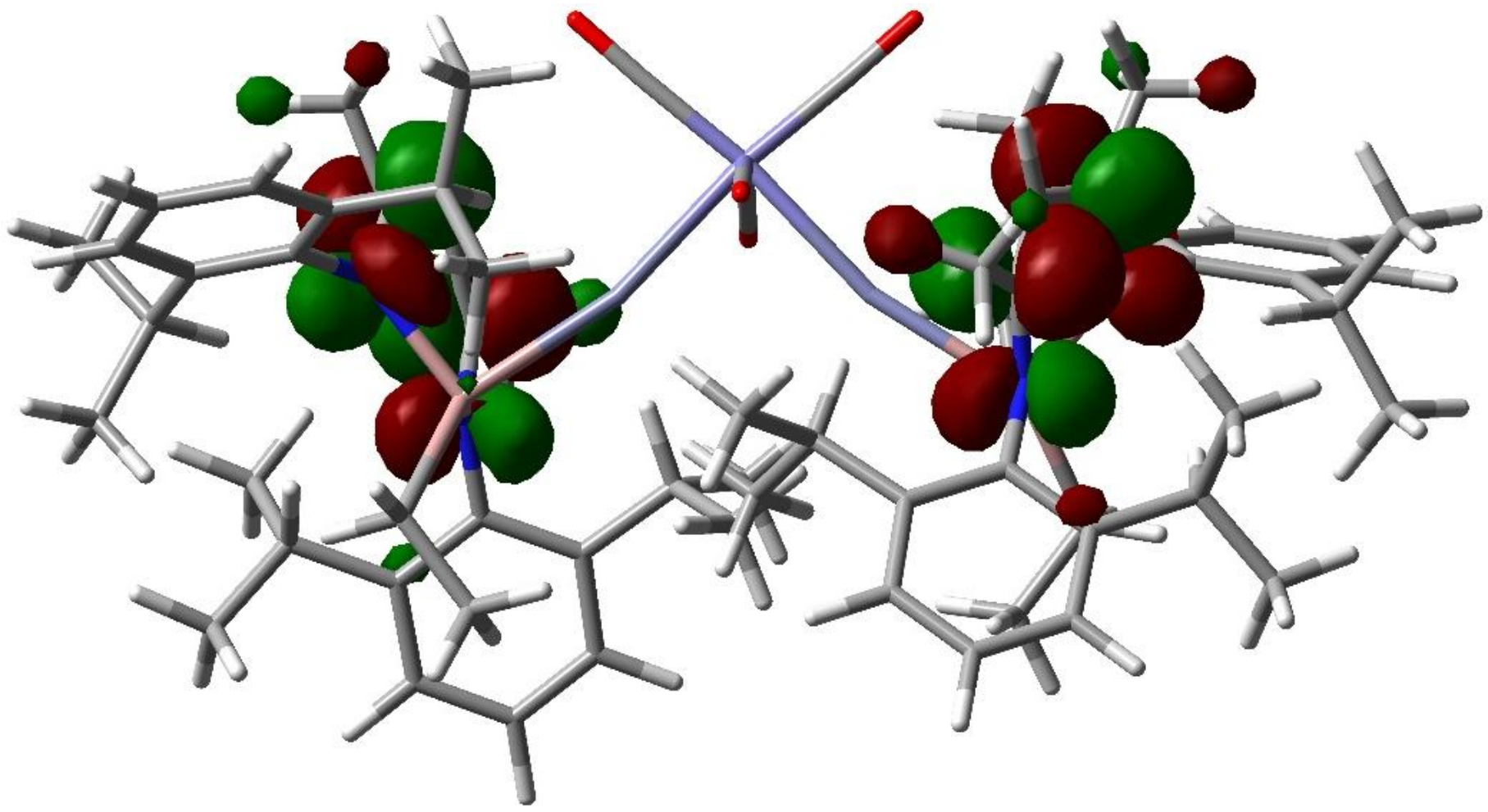

-0.90 eV

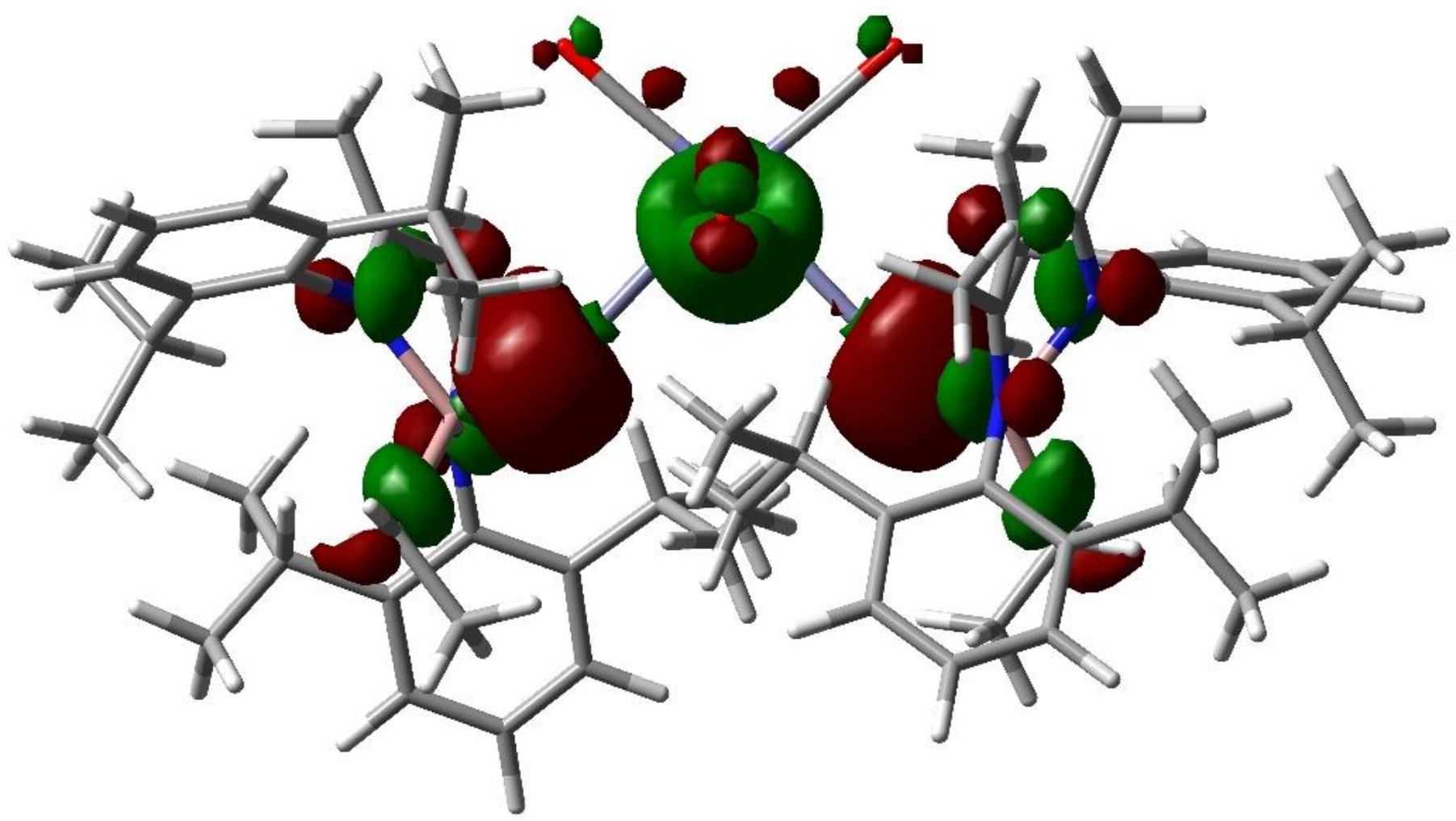

-4.95 eV

Figure S54. HOMO (below) and LUMO (above) of **2** calculated by means of PBE0-D3BJ(SMD,C_6H_6)/def2-TZVP level of theory (isovalue = 0.030 au). Hydrogen atoms are omitted for clarity.

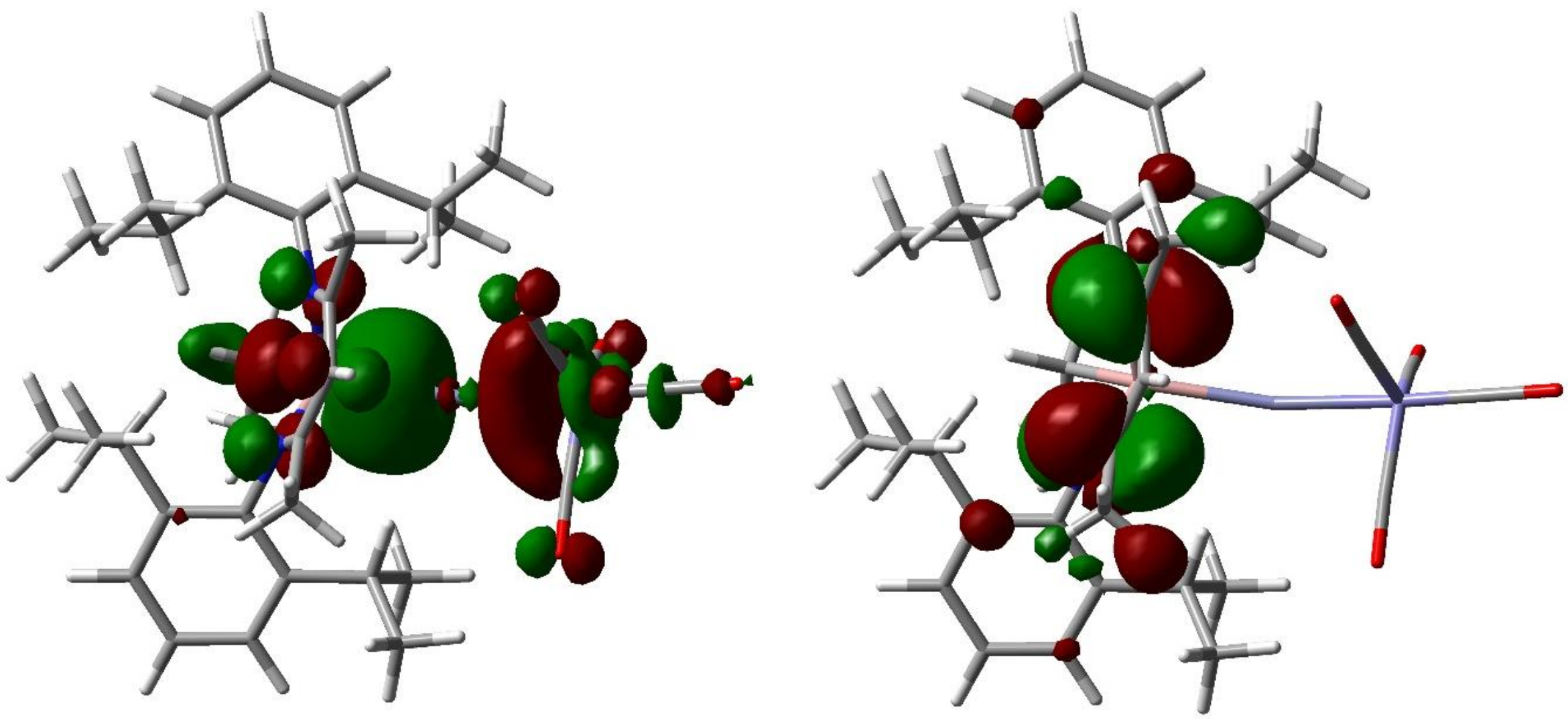

-3.67 eV 0.01 eV

Figure S55. HOMO (left) and LUMO (right) of the anionic part of **3** calculated by means of PBE0-D3BJ(SMD,C_6H_6)/def2-TZVP level of theory (isovalue = 0.030 au). Hydrogen atoms are omitted for clarity.

Cartesian coordinates of the optimized ground state geometry of **2** at PBE0-D3BJ/def2-SVP level of theory:

E(PBE0-D3BJ/def2-SVP) = -11754.914200

E(PBE0-D3BJ(SMD,C6H6)/def2-TZVP//PBE0-D3BJ/def2-SVP) = -11759.380805

Ga	-3.70108900	-0.25058700	-0.65856400
Zn	-1.64576200	-0.19791600	0.64827600
Fe	0.00003200	-0.00049800	2.39577300
O	0.46629200	-2.78818500	1.68685400
O	2.19290400	0.31810900	4.27713000
N	-4.92361200	-0.50948600	0.91677900
N	-4.06271800	1.73465500	-0.70976700
C	-4.78787000	0.38600000	1.89056900
C	-4.21437500	1.65815700	1.68912900
H	-4.11873000	2.27506900	2.58260400
C	-4.06260000	2.34909700	0.46055900
C	-5.28169600	0.05325800	3.26880100
H	-5.01376800	0.83688200	3.98614000
H	-4.86055800	-0.90225500	3.61103100
H	-6.37462000	-0.06894100	3.26342000
C	-3.88419600	3.83838100	0.55250100
H	-4.00454900	4.33073100	-0.42010300
H	-2.86723400	4.04653200	0.92341800
H	-4.59168200	4.27083300	1.27277400
C	-5.65584400	-1.70783500	1.13975400
C	-7.04635000	-1.72475600	0.90036400
C	-7.75256900	-2.90950100	1.12588000
H	-8.83128100	-2.92995400	0.95049600
C	-7.10957300	-4.06042100	1.56427900
H	-7.67780700	-4.97788100	1.73424200
C	-5.73698100	-4.03749200	1.77959000
H	-5.22969300	-4.94476800	2.11660400
C	-4.98992600	-2.87411700	1.57670300
C	-7.78615200	-0.50016400	0.40016400
H	-7.03529700	0.28465700	0.22452200
C	-8.78260300	0.03047500	1.43113400
H	-8.29412100	0.26836700	2.38733600
H	-9.57605500	-0.70546400	1.63780500
H	-9.26686700	0.94946300	1.06488300
C	-8.48264700	-0.78724400	-0.92840300
H	-9.00079400	0.11149400	-1.29518300
H	-9.23856300	-1.58122600	-0.82419900
H	-7.76362000	-1.10901800	-1.69624100
C	-3.49107400	-2.90897300	1.79595800
H	-3.13509300	-1.86563500	1.75672600
C	-3.09381200	-3.46880900	3.15918300
H	-2.00439600	-3.39512100	3.29710300
H	-3.36557100	-4.53131000	3.25778300
H	-3.57587200	-2.91800900	3.97996400
C	-2.80145300	-3.66901200	0.66276400
H	-3.02252200	-3.21957800	-0.31736200
H	-3.14086100	-4.71639600	0.63067200
H	-1.71078100	-3.67133400	0.80449200
C	-3.94753200	2.41664100	-1.95103500
C	-5.09030500	2.52094000	-2.77613100
C	-4.95424500	3.11345200	-4.03299500
H	-5.82627300	3.20307200	-4.68201200
C	-3.72456800	3.58671500	-4.47950700
H	-3.63821000	4.04177500	-5.46888000
C	-2.60901200	3.47234000	-3.66274500
H	-1.64215700	3.83863100	-4.01645100
C	-2.69419900	2.89034700	-2.39360900
C	-6.43720900	2.02412300	-2.29027400
H	-6.23906700	1.12410900	-1.68670500

C	-7.10096900	3.04665100	-1.36585200
H	-7.30079000	3.98742000	-1.90327500
H	-6.46745500	3.28133100	-0.49901000
H	-8.06032200	2.66212800	-0.98423800
C	-7.37355100	1.63100700	-3.42667500
H	-7.68879500	2.50386500	-4.01964500
H	-8.28723400	1.16985500	-3.02795500
H	-6.89943200	0.90947900	-4.10928700
C	-1.43527400	2.76918900	-1.55939000
H	-1.72467700	2.38309400	-0.57100200
C	-0.74902600	4.11743200	-1.35227900
H	-0.37509400	4.52753000	-2.30329200
H	0.11085300	4.01012500	-0.67668600
H	-1.43393600	4.85824900	-0.91518400
C	-0.47342700	1.75238700	-2.16902500
H	-0.12175100	2.07266900	-3.16261500
H	-0.95616200	0.77199500	-2.28792400
H	0.41915000	1.62438200	-1.53278400
C	-4.35648900	-1.14358200	-2.30313400
H	-4.46045200	-2.22004700	-2.08342200
H	-5.37851700	-0.78354100	-2.51028000
C	-3.45723800	-0.92595800	-3.51675600
H	-3.37485700	0.14178100	-3.77730000
H	-3.82816600	-1.44937200	-4.41457400
H	-2.43277000	-1.28990000	-3.33340800
C	0.27131900	-1.66814800	1.91542200
C	1.34025600	0.19748200	3.51499300
Ga	3.70093600	0.25093500	-0.65856000
Zn	1.64570600	0.19737600	0.64831700
O	-0.46617700	2.78731700	1.68734100
O	-2.19304800	-0.31944200	4.27682400
N	4.92353900	0.50918400	0.91688300
N	4.06269000	-1.73433200	-0.71038400
C	4.78758300	-0.38655900	1.89040800
C	4.21401500	-1.65861200	1.68853100
H	4.11817900	-2.27577900	2.58181100
C	4.06235500	-2.34916900	0.45972400
C	5.28124200	-0.05424600	3.26880100
H	5.01273500	-0.83782600	3.98597400
H	4.86057600	0.90145700	3.61107500
H	6.37424000	0.06732800	3.26367100
C	3.88374200	-3.83845900	0.55115800
H	4.00421100	-4.33051500	-0.42158000
H	2.86666900	-4.04654700	0.92181000
H	4.59100800	-4.27125000	1.27144100
C	5.65574600	1.70746300	1.14027200
C	7.04630700	1.72442900	0.90112700
C	7.75249600	2.90911900	1.12698900
H	8.83123500	2.92960300	0.95177300
C	7.10944000	4.05996700	1.56549900
H	7.67766000	4.97738900	1.73571300
C	5.73681600	4.03701300	1.78057000
H	5.22946900	4.94422400	2.11766900
C	4.98978300	2.87368200	1.57733900
C	7.78617900	0.49996700	0.40070700
H	7.03538300	-0.28488200	0.22493700
C	8.78272300	-0.03077600	1.43153400
H	8.29431700	-0.26879400	2.38774400
H	9.57617900	0.70514700	1.63824600
H	9.26698700	-0.94970100	1.06512700
C	8.48260100	0.78736000	-0.92782900
H	9.00081800	-0.11125900	-1.29480500
H	9.23843700	1.58140300	-0.82348600

H	7.76351800	1.10922000	-1.69557800
C	3.49090200	2.90854900	1.79634800
H	3.13487300	1.86524000	1.75674300
C	3.09345900	3.46796500	3.15969900
H	2.00399700	3.39448100	3.29735000
H	3.36545000	4.53036800	3.25875600
H	3.57521600	2.91674600	3.98037600
C	2.80148500	3.66895700	0.66328400
H	3.02277900	3.21988100	-0.31695600
H	3.14083400	4.71637600	0.63162700
H	1.71078500	3.67115700	0.80478400
C	3.94764600	-2.41589800	-1.95189400
C	5.09065600	-2.52044600	-2.77663600
C	4.95477100	-3.11259800	-4.03369300
H	5.82697400	-3.20241700	-4.68245000
C	3.72505300	-3.58524900	-4.48072300
H	3.63884800	-4.04004000	-5.47023400
C	2.60926900	-3.47064300	-3.66429800
H	1.64239200	-3.83647700	-4.01841700
C	2.69427200	-2.88901900	-2.39498700
C	6.43761300	-2.02434500	-2.29018900
H	6.23963000	-1.12450100	-1.68630700
C	7.10075400	-3.04748000	-1.36599600
H	7.30043400	-3.98808700	-1.90375700
H	6.46690800	-3.28232600	-0.49944400
H	8.06011400	-2.66342100	-0.98393300
C	7.37441200	-1.63116000	-3.42619300
H	7.68941100	-2.50391100	-4.01945100
H	8.28820200	-1.17058100	-3.02705500
H	6.90078300	-0.90914000	-4.10862500
C	1.43515500	-2.76761500	-1.56109900
H	1.72442700	-2.38170100	-0.57259100
C	0.74848500	-4.11567900	-1.35431500
H	0.37486100	-4.52566800	-2.30549500
H	-0.11166600	-4.00816500	-0.67910000
H	1.43300400	-4.85667400	-0.91691600
C	0.47371700	-1.75050600	-2.17087400
H	0.12216300	-2.07064800	-3.16455100
H	0.95669300	-0.77020800	-2.28959700
H	-0.41895600	-1.62236800	-1.53478900
C	4.35702900	1.14408500	-2.30277600
H	4.46187900	2.22038400	-2.08267700
H	5.37877800	0.78322200	-2.50987600
C	3.45778700	0.92759400	-3.51659900
H	3.37466800	-0.13999400	-3.77753300
H	3.82922500	1.45105200	-4.41418100
H	2.43355100	1.29221500	-3.33327600
C	-0.27112200	1.66725800	1.91575700
C	-1.34033100	-0.19867300	3.51478200

Cartesian coordinates of the optimized ground state geometry of anionic part of **3** at PBE0-D3BJ/def2-SVP level of theory:

E(PBE0-D3BJ/def2-SVP) = -6735.459152

E(PBE0-D3BJ(SMD,C6H6)/def2-TZVP//PBE0-D3BJ/def2-SVP) = -6738.075474

Ga	-0.54939800	-0.02261000	-0.77496400
Zn	1.84645300	-0.38959600	-0.43072100
Fe	4.05348600	-0.34581400	0.24265700
N	-0.82746500	1.54950000	0.50499200
N	-1.40956700	-1.28298500	0.58118900
C	-0.50963500	1.33843900	1.77396300
C	-0.48168300	0.05212800	2.35485700

H	-0.14122100	0.01346600	3.38926600
C	-1.03814100	-1.13635700	1.84637900
C	-0.11677800	2.48337100	2.66460500
H	0.98516300	2.48297700	2.71481700
H	-0.49875100	2.34415400	3.68481000
H	-0.44903500	3.45227500	2.27290400
C	-1.19044400	-2.28532700	2.80703500
H	-0.33771100	-2.97300100	2.69181900
H	-2.10065100	-2.86558800	2.60194100
H	-1.20530700	-1.93299300	3.84571200
C	-1.06520500	2.85556900	0.01067400
C	-0.03095500	3.59191400	-0.60549700
C	-0.31706200	4.87583500	-1.08033800
H	0.48215600	5.45453800	-1.55107800
C	-1.58815900	5.42515900	-0.96442000
H	-1.78875200	6.43301100	-1.33710500
C	-2.60670800	4.67900500	-0.38033300
H	-3.60962800	5.10636400	-0.30021300
C	-2.36994700	3.39048200	0.10381900
C	1.35958400	3.02186400	-0.78735800
H	1.37915500	2.04001000	-0.29034500
C	2.44692600	3.86493500	-0.12852000
H	3.41154300	3.34018900	-0.20291800
H	2.23679700	4.02531900	0.93872900
H	2.54972500	4.84977900	-0.61380600
C	1.65363500	2.78311400	-2.26839000
H	2.63623800	2.30766800	-2.40234900
H	1.65058200	3.73162300	-2.83060700
H	0.89499300	2.12552700	-2.72067700
C	-3.48953200	2.59312900	0.74289200
H	-3.14247400	1.55019400	0.78840600
C	-3.75321900	3.05027400	2.17798300
H	-2.84705100	2.97727200	2.79540600
H	-4.53398900	2.43101800	2.64879400
H	-4.09077300	4.09979000	2.20127000
C	-4.77079200	2.62203800	-0.08475300
H	-4.59052700	2.26386900	-1.10938100
H	-5.19661000	3.63609600	-0.15037500
H	-5.53753300	1.97871200	0.37431300
C	-2.03204700	-2.46506600	0.11500000
C	-1.26528400	-3.60571500	-0.20931200
C	-1.92639600	-4.73486300	-0.70534000
H	-1.34012800	-5.62245100	-0.95679200
C	-3.30201100	-4.74578000	-0.89250700
H	-3.79792400	-5.63888400	-1.28136300
C	-4.04391100	-3.60528900	-0.59894300
H	-5.12262400	-3.60941400	-0.76745100
C	-3.42928900	-2.45339700	-0.10492400
C	0.24504100	-3.63376100	-0.08273400
H	0.55996800	-2.70544000	0.42028000
C	0.74539100	-4.81135700	0.75303400
H	1.82656200	-4.70849900	0.92146600
H	0.56721500	-5.77194000	0.24253600
H	0.24073500	-4.85979700	1.73041700
C	0.90667700	-3.62941500	-1.46173300
H	2.00147500	-3.60237600	-1.35469300
H	0.59408000	-2.75132200	-2.04729300
H	0.63549700	-4.53117600	-2.03497700
C	-4.23360900	-1.20507200	0.19687300
H	-3.55390800	-0.35873100	0.01593900
C	-4.64532000	-1.14091600	1.66799100
H	-3.76866200	-1.14849900	2.33077800
H	-5.28402500	-1.99870400	1.93575700

H	-5.20999300	-0.21720300	1.87504900
C	-5.44053900	-1.03776200	-0.71922800
H	-5.14951600	-1.10789400	-1.77831800
H	-5.90703100	-0.05484400	-0.56005300
H	-6.21496500	-1.79816100	-0.52731300
C	-1.84118800	0.24125200	-2.28260700
H	-2.83998500	0.46789100	-1.87036300
H	-1.52769300	1.15831300	-2.81227200
C	-1.91702800	-0.93136200	-3.25542100
H	-0.92682200	-1.16575600	-3.68174300
H	-2.59528600	-0.74135000	-4.10727600
H	-2.27115400	-1.85111400	-2.76101400
O	4.39365200	0.53894800	-2.51015000
C	4.22280000	0.18479600	-1.41058500
O	2.94109600	1.41817400	2.27270900
C	3.35494400	0.70904800	1.43922600
O	3.65702800	-3.19574700	0.64999300
C	3.78146600	-2.04634400	0.47705300
O	6.83530200	-0.15215400	1.04544800
C	5.72468300	-0.23028900	0.72465000

VI. References

1 (a) N. J. Hardman, B. E. Eichler and P. P. Power, *Chem. Commun.*, 2000, 1991-1992; (b) A. Guerrero, D. L. Hughes and M. Bochmann, *Organometallics*, 2006, **25**, 1525-1527; (c) J. P. Collman, *Acc. Chem. Res.*, 1975, **8**, 342-347; (d) H. B. Chin and R. Bau, *J. Am. Chem. Soc.*, 1976, **98**, 2434-2439; (e) G. Rabe, H. W. Roesky, D. Stalke, F. Pauer and G. M. Sheldrick, *J. Organomet. Chem.*, 1991, **403**, 11-19.

2 G. R. Fulmer, A. J. M. Miller, N. H. Sherden, H. E. Gottlieb, A. Nudelman, B. M. Stoltz, J. E. Bercaw and K. I. Goldberg, *Organometallics* 2010, **29**, 2176.

3 G. M. Sheldrick, *Acta Crystallogr., Sect. A.* 1990, **A46**, 467.

4 G. M. Sheldrick, SHELX 2017, Program for the Refinement of Crystal Structures, University of Göttingen: Göttingen, Germany, 2017.

5 G. M. Sheldrick, *Acta Crystallogr., Sect. A* 2008, **A64**, 112.

6 C. B. Hübschle, G. M. Sheldrick and B. Dittrich, *J. Appl. Crystallogr.* 2011, **44**, 1281.

7 M. J. Frisch, G. W. Trucks, H. B. Schlegel, G. E. Scuseria, M. A. Robb, J. R. Cheeseman, G. Scalmani, V. Barone, G. A. Petersson, H. Nakatsuji, X. Li, M. Caricato, A. V. Marenich, J. Bloino, B. G. Janesko, R. Gomperts, B. Mennucci, H. P. Hratchian, J. V. Ortiz, A. F. Izmaylov, J. L. Sonnenberg, D. Williams-Young, F. Ding, F. Lipparini, F. Egidi, J. Goings, B. Peng, A. Petrone, T. Henderson, D. Ranasinghe, V. G. Zakrzewski, J. Gao, N. Rega, G. Zheng, W. Liang, M. Hada, M. Ehara, K. Toyota, R. Fukuda, J. Hasegawa, M. Ishida, T. Nakajima, Y. Honda, O. Kitao, H. Nakai, T. Vreven, K. Throssell, J. A. Montgomery, Jr., J. E. Peralta, F. Ogliaro, M. J. Bearpark, J. J. Heyd, E. N. Brothers, K. N. Kudin, V. N. Staroverov, T. A. Keith, R. Kobayashi, J. Normand, K. Raghavachari, A. P. Rendell, J. C. Burant, S. S. Iyengar, J. Tomasi, M. Cossi, J. M. Millam, M. Klene, C. Adamo, R. Cammi, J. W. Ochterski, R. L. Martin, K. Morokuma, O. Farkas, J. B. Foresman and D. J. Fox, Gaussian, Inc., Wallingford CT, 2016.

8 G. te Velde, F. M. Bickelhaupt, E. J. Baerends, C. Fonseca Guerra, S. J. A. van Gisbergen, J. G. Snijders and T. Ziegler, *J. Comput. Chem.* 2001, **22**, 931-967.

9 E.J. Baerends, T. Ziegler, A. J. Atkins, J. Autschbach, O. Baseggio, D. Bashford, A. Bérces, F. M. Bickelhaupt, C. Bo, P. M. Boerrigter, C. Cappelli, L. Cavallo, C. Daul, D. P. Chong, D. V. Chulhai, L. Deng, R. M. Dickson, J.M. Dieterich, F. Egidi, D.E. Ellis, M. van Faassen, L. Fan, T.H. Fischer, A. Förster, C. Fonseca Guerra, M. Franchini, A. Ghysels, A. Giammona, S. J. A. van Gisbergen, A. Goez, A. W. Götz, J. A. Groeneveld, O. V. Gritsenko, M. Grüning, S. Gusarov, F. E. Harris, P. van den Hoek, Z. Hu, C. R. Jacob, H. Jacobsen, L. Jensen, L. Joubert,

J. W. Kaminski, G. van Kessel, C. König, F. Kootstra, A. Kovalenko, M. V. Krykunov, P. Lafiosca, E. van Lenthe, D. A. McCormack, M. Medves, A. Michalak, M. Mitoraj, S. M. Morton, J. Neugebauer, V. P. Nicu, L. Noodleman, V. P. Osinga, S. Patchkovskii, M. Pavanello, C. A. Peeples, P. H. T. Philipsen, D. Post, C. C. Pye, H. Ramanantoanina, P. Ramos, W. Ravenek, M. Reimann, J. I. Rodríguez, P. Ros, R. Rüger, P. R. T. Schipper, D. Schlüns, H. van Schoot, G. Schreckenbach, J. S. Seldenthuis, M. Seth, J. G. Snijders, M. Solà, M. Stener, M. Swart, D. Swerhone, V. Tognetti, G. te Velde, P. Vernooijs, L. Versluis, L. Visscher, O. Visser, F. Wang, T. A. Wesolowski, E. M. van Wezenbeek, G. Wiesenekker, S. K. Wolff, T. K. Woo, A. L. Yakovlev, 2020.

10 C. Adamo and V. Barone, *J. Chem. Phys.* 1999, **110**, 6158-6170.

11 S. Grimme, S. Ehrlich and L. Goerigk, *J. Comput. Chem.* 2011, **32**, 1456-1465.

12 F. Weigend and R. Ahlrichs, *Phys. Chem. Chem. Phys.* 2005, **7**, 3297-3305.

13 F. Weigend, *Phys. Chem. Chem. Phys.* 2006, **8**, 1057-1065.

14 F. Neese, *WIREs Comput. Mol. Sci.*, 2022, **12**, e1606.

15 A. V. Marenich, C. J. Cramer and D. G. Truhlar, *J. Phys. Chem. B* 2009, **113**, 6378-6396.

16 F. M. Bickelhaupt and E. J. Baerends, *Rev. Comput. Chem.* 2000, **15**, 1-86.

17 M. P. Mitoraj, A. Michalak and T. Ziegler, *J. Chem. Theory Comput.*, 2009, **5**, 962-975.

18 A. D. Becke, *Phys. Rev. A* 1988, **38**, 3098-3100.

19 C. Lee, W. Yang and R. G. Parr, *Phys. Rev. B* 1988, **37**, 785-789.

20 B. Miehlich, A. Savin, H. Stoll and H. Preuss, *Chem. Phys. Lett.* 1989, **157**, 200-206.

21 E. Van Lenthe and E. J. Baerends, *J. Comput. Chem.* 2003, **24**, 1142-1156.

22 E. v. Lenthe, A. Ehlers and E.-J. Baerends, *J. Chem. Phys.* 1999, **110**, 8943-8953.
